# Enriching Chemical Space of Bioactive Scaffolds by New Ring Systems: Benzazocines and Their Metal Complexes as Potential Anticancer Drugs

**DOI:** 10.1021/acs.inorgchem.2c03134

**Published:** 2022-12-06

**Authors:** Irina Kuznetcova, Marija Ostojić, Nevenka Gligorijević, Sandra Aranđelović, Vladimir B. Arion

**Affiliations:** † Institute of Inorganic Chemistry of the University of Vienna, Währinger Strasse 42, 1090 Vienna, Austria; ‡ Department of Experimental Oncology, Institute for Oncology and Radiology of Serbia, Pasterova 14, 11000 Belgrade, Serbia

## Abstract

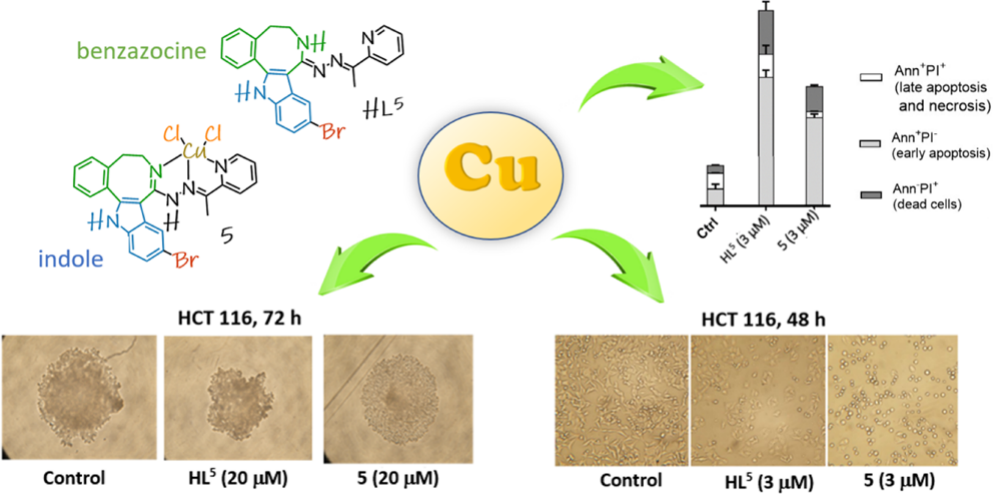

The search for new scaffolds of medicinal significance combined with molecular shape enhances their innovative potential and continues to attract the attention of researchers. Herein, we report the synthesis, spectroscopic characterization (^1^H and ^13^C NMR, UV–vis, IR), ESI-mass spectrometry, and single-crystal X-ray diffraction analysis of a new ring system of medicinal significance, 5,6,7,9-tetrahydro-8*H*-indolo[3,2-*e*]benzazocin-8-one, and a series of derived potential ligands (**HL**^**1**^**–HL**^**5**^), as well as ruthenium(II), osmium(II), and copper(II) complexes (**1a**, **1b**, and **2–5**). The stability of compounds in 1% DMSO aqueous solutions has been confirmed by ^1^H NMR and UV–vis spectroscopy measurements. The antiproliferative activity of **HL**^**1**^**–HL**^**5**^ and **1a**, **1b**, and **2–5** was evaluated by in vitro cytotoxicity tests against four cancer cell lines (LS-174, HCT116, MDA-MB-361, and A549) and one non-cancer cell line (MRC-5). The lead compounds **HL**^**5**^ and its copper(II) complex **5** were 15× and 17×, respectively, more cytotoxic than cisplatin against human colon cancer cell line HCT116. Annexin V-FITC apoptosis assay showed dominant apoptosis inducing potential of both compounds after prolonged treatment (48 h) in HCT116 cells. **HL**^**5**^ and **5** were found to induce a concentration- and time-dependent arrest of cell cycle in colon cancer cell lines. Antiproliferative activity of **5** in 3D multicellular tumor spheroid model of cancer cells (HCT116, LS-174) superior to that of cisplatin was found. Moreover, **HL**^**5**^ and **5** showed notable inhibition potency against glycogen synthase kinases (GSK-3α and GSK-3β), tyrosine-protein kinase (Src), lymphocyte-specific protein-tyrosine kinase (Lck), and cyclin-dependent kinases (Cdk2 and Cdk5) (IC_50_ = 1.4–6.1 μM), suggesting their multitargeted mode of action as potential anticancer drugs.

## Abstract 2

Incorporation of an 8-membered azocine ring into paullone scaffold enriches the available chemical space of bioactive scaffolds, and, in combination with substitutions at the lactam unit, Schiff base C=N bond and bromination at position 10 are effective tools for fine-tuning the anticancer potency of copper(II)-based drug candidates. Complex **5** showed higher antiproliferative activity in the 3D culture model of MCTS than clinical drug cisplatin, indicating its suitability for in vivo assays. The compound causes cell cycle perturbation and effectively inhibits protein kinases Cdk2, Cdk5, GSK-3α, GSK-3β, Src, and Lck.

## Introduction

Rings are favored building blocks of approved drugs and compounds in clinical trials^1−8^ but also used in compounds at the early stage of development.^9−11^ They determine the electronic distribution, shape, and scaffold flexibility. These electronic and geometric features are often key factors determining molecular properties such as lipophilicity or polarity, which may be responsible for the molecule’s reactivity and toxicity.^12^ Generally, the number of rings or ring systems (for definition, see ref (1)) in drugs is smaller when compared with those in clinical trials. Systematic changes of up to two atoms on existing drug and clinical trial ring systems are expected to lead to future clinical trial scaffolds, which are predicted to cover ∼50% of the novel ring systems entering clinical trials. An innovative drug was defined recently as a drug in which both the scaffold and the molecular shape had not been observed in a previous drug molecule.^3^ Recent analyses of all approved drugs over the last 80 years revealed that the number of new scaffolds combined with molecular shape has increased over time, even though diversity of topological shapes in the set of known drugs remains low.^3^

**Chart 1:**
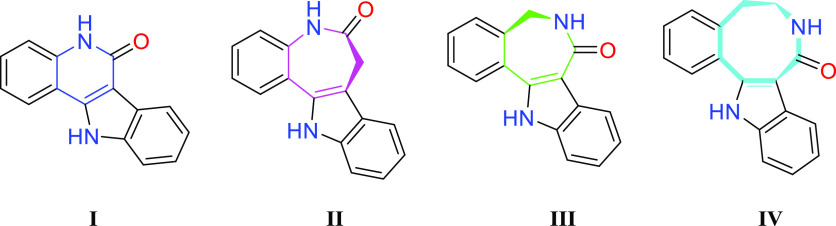
7*H*-indolo[3,2-*c*]quinoline-6(5*H*)-one (**I**), 7,12-dihydroindolo[3,2-*d*][1]benzazepine-6(5*H*)-one (**II**), 5,8-dihydroindolo[3,2-*d*][2]benzazepine-7(6*H*)-one (**III**), and 5,6,7,9-tetrahydro-8*H*-indolo[3,2-*e*]benzazocin-8-one (**IV**).

Inspection of the top 100 most frequently used rings and ring systems from small molecule drugs listed in the FDA Orange book before January 2020^1,3^ did not reveal indoloquinoline or indolobenzazepine scaffolds (I–III in Chart 1).

**Chart 2:**
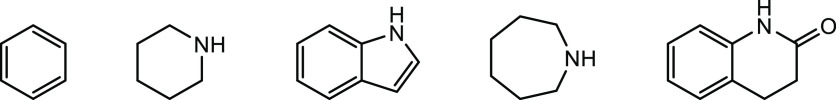
Ring and ring systems involved in indoloquinoline and indolobenzazepine scaffolds, which are among the top 100 most frequently used ring systems from small molecule drugs sorted by descending frequency.

However, rings and ring systems that the latter two classes of compounds are built upon (Chart 2) are among the top 100 rings and ring systems.

The lack of larger eight-membered azocine and nine-membered azonine rings among them is likely due to general conservatism in the design and preparation of new compounds, which are structurally similar to already approved drugs. Involvement of 3D metrics^13^ such as shape or molecular electrostatic potentials in new bioactive scaffolds should increase their diversity and innovative value. Only 5% of drugs do not contain any sp^3^ carbons, while 40% of drugs do not contain any sp^3^ carbons in a ring.^1^ At the same time, it is widely believed that introducing three dimensionality should have a positive impact on the clinical success of a potential drug.^14^ Escape from flatland has been realized by us via substitution of the six-membered N-containing ring in indoloquinolines by the seven-membered azepine ring in indolobenzazepines. The effect of this structural change on antiproliferative activity and underlying mechanism of cytotoxicity has been elucidated recently.^15−17^ Moreover, it was shown that the position of the sp^3^-hybridized carbon atom in a seven-membered N-containing ring has a marked effect on scaffold folding,^18^ which distinctly impacts the molecular shape of the drug molecule.

The most widely used chemotherapeutics continue to be cisplatin- and platinum-based complexes, such as carboplatin and oxaliplatin. However, the use of these complexes is limited by their toxicity and acquired drug resistance.^19^ Other third row transition metals, that is, osmium, iridium, and gold,^20−24^ as well as second row transition metals, that is, ruthenium and rhodium,^21−23,25,26^ were reported to be suitable for the development of potential anticancer drugs. The serious toxicity of the Pt-based drugs has stimulated extensive search for biologically essential metals and, in particular, of those of the first row, which play important biological roles in living organisms, in order to improve the pharmacological properties and reduce the general toxicity of the potential anticancer drugs. One of these metals, which attracted much attention over the last few years is copper.^23,27^

**Scheme 1:**
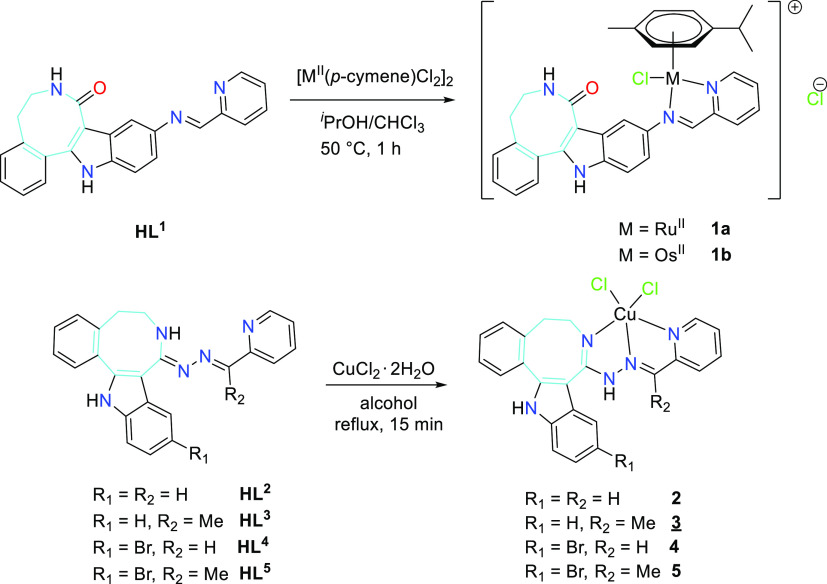
Synthesis of Ruthenium(II) and Osmium(II) Complexes **1a** and **1b** as Well as Copper(II) Complexes **2–5** from Indolobenzazocine-Derived Ligands **HL**^**1**^–**HL**^**5**^; The Underlined Number Indicates the Compound Studied by SC-XRD

Herein, we report on the synthesis and full characterization of a series of Schiff bases **HL**^**1**^**–HL**^**5**^ and their ruthenium(II), osmium(II), and copper(II) complexes **1a**, **1b**, and **2**–**5** in Scheme 1 based on new four ring fused systems, which are called indolo[3,2-*e*]benzazocines and contain an eight-membered azocine ring with two sp^3^ carbons in it, and evaluation of their anticancer potential. In particular, their cytotoxicity in a panel of human cancer cells including multidrug-resistant cell lines (LS-174 and A549) obtained from solid tumors in 2D and 3D culture cells, the ability to disturb cell cycle progression and apoptosis/necrosis induction, and kinase inhibition potential were investigated. Their performance was compared with that of previously reported ring systems featuring a six-membered flat and a seven-membered folded N-containing rings, namely, indolo[3,2-*c*]quinolines, indolo[3,2-*d*][1]benzazepines (paullones), indolo[3,2-*d*][2]benzazepines, and indolo[2,3-*d*]benzazepines.

## Results and Discussion

### Synthesis of Ligands, Ruthenium(II), Osmium(II), and Copper(II) Complexes

The multistep synthetic pathway to **HL**^**1**^–**HL**^**5**^ and complexes **1a**, **1b**, and **2–5** is depicted in Scheme S1. This route can be subdivided into three distinct segments: (i) preparation of benzazocines **f**_**1**_**–f**_**3**_, (ii) building up of a suitable metal binding site and generation of potential bi- and tridentate ligands **HL**^**1**^–**HL**^**5**^, and (iii) synthesis of ruthenium(II) and osmium(II) complexes **1a** and **1b** as well as copper(II) complexes **2–5**. Benzazocines **f**_**1**_**–f**_**3**_ were prepared in six steps. First, 5-nitro-indole-3-carboxaldehyde, indole-3-carboxaldehyde, and 5-bromo-indole-3-carboxaldehyde were tosylated to yield species **a**_**1**_–**a**_**3**_, which were further oxidized by sodium chlorite into carboxylic acids **b**_**1**_–**b**_**3**_. Then, by reaction of **b**_**1**_–**b**_**3**_ with 2-iodobenzeneethylamine,^28^ compounds **c**_**1**_–**c**_**3**_ were obtained in excellent yields (88–98%). Boc-protection of amide nitrogen in **c**_**1**_–**c**_**3**_ and isolation of **d**_**1**_–**d**_**3**_ were achieved in 83–95% yields. Palladium-catalyzed Heck-cyclization of **d**_**1**_–**d**_**3**_ afforded eight-membered-ring-containing compounds **e**_**1**_–**e**_**3**_ in 21, 54, and 44% yield, respectively. Finally, indolobenzazocinones **f**_**1**_**–f**_**3**_ were synthesized in 54–90% yield by deprotection of compounds **e**_**1**_–**e**_**3**_ with trifluoroacetic acid and tetra*-n*-butylammonium fluoride (TBAF). The formation of an eight-membered ring followed by deprotection was confirmed in addition to ^1^H and ^13^C NMR spectra by SC-XRD analysis of **f**_**2**_ (see Figure S1 in the Supporting Information). Creation of a suitable metal binding site was realized in two different ways. The first way was explored for the synthesis of **HL**^**1**^ and was realized in two steps. In the first step, by catalytic hydrogenation of **f**_**1**_, amine **g**_**1**_ was produced in 94% yield. Then, in the second step, **g**_**1**_ was allowed to enter the condensation reaction with 2-formylpyridine to give **HL**^**1**^ as a bright-yellow powder in 55% yield. The second way was followed upon the synthesis of chelating ligands **HL**^**2**^–**HL**^**5**^, which were assembled in three steps. First, indolobenzazocinones **f**_**2**_ and **f**_**3**_ were subjected to thionation by P_4_S_10_ to give **g**_**2**_ and **g**_**3**_ in 73 and 37% yields, respectively. Thionation of lactam group was confirmed by ESI mass spectra (see the Experimental Section) and by SC-XRD analysis of **g2** with the results shown in Figure S2 in the Supporting Information. Treatment of **g**_**2**_ and **g**_**3**_ with hydrazine hydrate in chloroform afforded hydrazine derivatives **h**_**2**_ and **h**_**3**_ in almost quantitative yields. It is worth noting that under analogous conditions, the reactions of seven-membered-ring-containing indolobenzazepines produced only 20–30% of the required thionated products.^18^ Even more disappointingly, the further reaction of the thionated indolobenzazepine with hydrazine hydrate failed. This reaction was realized only when absolute hydrazine was used. Upon use of hydrazine hydrate, sulfur atom was replaced by oxygen and starting lactams were recovered.^18^ Finally, Schiff base condensation reactions of **h**_**2**_ and **h**_**3**_ with 2-formylpyridine and 2-acetylpyridine afforded four potentially tridentate ligands **HL**^**2**^**–HL**^**5**^ in very good yields (72–86%).

^1^H and ^13^C NMR spectra of **HL**^**1**^**–HL**^**5**^ were in agreement with the suggested molecular structures of *C*_1_ symmetry (Figures S3–S12). Positive ion ESI mass spectra showed peaks with *m*/*z* 366.19 (calcd *m*/*z* for [C_23_H_19_N_5_]^+^ 366.17), 380.21 (calcd *m*/*z* for [C_24_H_21_N_5_]^+^), 446.13 (calcd *m*/*z* for [C_23_H_18_BrN_5_]^+^ 446.08), and 460.15 (calcd *m*/*z* for [C_24_H_20_BrN_5_]^+^ 460.10) attributed to the protonated molecular ions [M + H]^+^ (Figures S13–S16). The purity of **HL**^**1**^**–HL5** (≥95%) was confirmed by elemental analysis. The ruthenium(II) and osmium(II) complexes **1a** and **1b** were prepared in 68 and 72% yields by reactions of **HL**^**1**^ with [M^II^(*p*-cymene)Cl_2_]_2_, where M = Ru, Os, in 2-propanol and methanol, respectively. The synthesis of copper(II) complexes **2–5** in 70–80% yield was performed by reactions of the corresponding ligands **HL**^**2**^–**HL**^**5**^ with CuCl_2_·2H_2_O in 2-propanol, ethanol, or methanol as described in the Experimental Section. The positive ion ESI mass spectra of **1a** and **1b** showed peaks with *m*/*z* 637.18 (calcd *m*/*z* for [C_33_H_31_N_4_ORu]^+^ 637.14) and 727.21 (calcd *m*/*z* for [C_33_H_31_N_4_OOs]^+^ 727.19) due to [Ru^II^Cl(**HL**^**1**^)]^+^ and [Os^II^Cl(**HL**^**1**^)]^+^, respectively (Figures S17 and S18). The mass spectra of **2**–**5** contain peaks at *m*/*z* 463.11 (calcd *m*/*z* for [C_23_H_19_CuClN_5_]^+^ 463.06), 477.12 (calcd *m*/*z* for [C_24_H_21_CuClN_5_]^+^ 477.08), 543.05 (calcd *m*/*z* for [C_23_H_18_BrCuClN_5_]^+^ 542.96), and 557.04 (calcd *m*/*z* for [C_24_H_20_CuClN_5_]^+^ 556.99) attributed to [Cu^II^Cl(**HL**)]^+^, where **HL** = **HL**^**2**^ −**HL**^**5**^, respectively (Figures S19–S22). The purity (≥95%) of **1a** and **1b** was proven by elemental analysis and NMR spectroscopy (Figures S23–S26), while that of **2–5** was proven by elemental analysis. In addition, the purity of **HL**^**5**^ and **5** was confirmed by HPLC coupled with high-resolution ESI mass spectrometry (HR ESI MS) (Figures S27 and S28). Finally, the coordination geometry and the molecular shape of **3** were established by SC-XRD analysis.

### X-ray Crystallography

**Figure 1:**
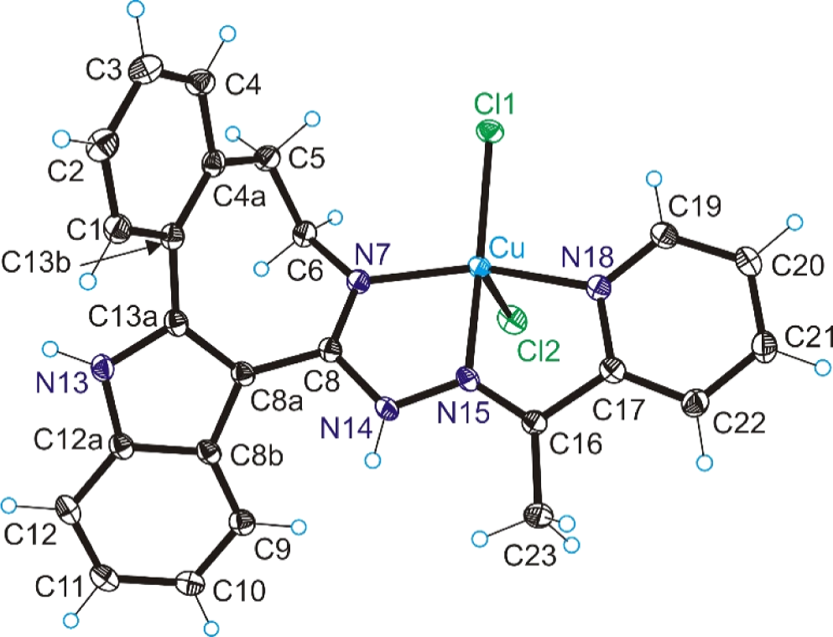
ORTEP view of complex **3** with thermal ellipsoids at 50% probability level. Selected bond distances (Å), bond angles (deg), and torsion angles (deg): Cu–N7 1.9903(14), Cu–N15 1.9775(14), Cu–N18 2.0458(15), Cu–Cl1 2.2633(5), Cu–Cl2 2.5138(5); N7–C8 1.297(2), C8–N14 1.388(2), N14–N15 1.3677(19), N15–C16 1.288(2), C16–C17 1.483(2), C17–N18 1.360(2); N7–Cu–N15 79.62(6), N15–Cu–N18 78.06(6), θ_C4a–C5–C6–N7_–55.9(2).

The result of SC-XRD study of complex **[CuCl**_**2**_**(HL**^**3**^**)]·3MeOH** (**3**) is shown in Figure 1, with pertinent bond distances (Å), bond angles, and torsion angles (deg) quoted in the legend. Details of data collection and refinement are collected in Table S1. The complex crystallized in the monoclinic space group *P*2_1_/*c* with one molecule of the complex and three molecules of methanol in the asymmetric unit. The copper(II) complex is five-coordinate square-pyramidal (τ_5_ = 0.14)^29^ with nitrogen atoms N7, N15, and N18 and chlorido coligand Cl1 occupying the four sites of pyramide basis and another chlorido coligand Cl2 in the apical position. The bond lengths Cu–N7, Cu–N15, Cu–N18, and Cu–Cl1 (see legend to Figure 1) are by 0.024–0.062 Å longer than similar bonds in a square-planar copper(II) complex with a structurally related indolobenzazepine [Cu–N6 = 1.965(3), Cu–N14 = 1.950(3), Cu–N17 = 2.022(3), Cu–Cl = 2.2016(8), Cu–Cl = 2.2016(8)], likely due to higher electrostatic repulsions in a five-coordinate copper(II) complex than in a four-coordinate one (**[CuCl(HL**^**3**^**)]Cl** in ref (18)).

The dihedral angle between mean planes through C1–C2–C3–C4–C4a–C13b and N7–N15–N18–Cu is 100.75(5)° compared to 113.69(9)° in the reference indolobenzazepine-derived copper(II) complex (**[CuCl(HL**^**3**^**)]Cl** in ref (18)). The overlay of the structures of **3** and **[CuCl(HL**^**3**^**)]Cl**(18) illustrating the difference in molecular shapes of the two complexes is presented in Figure S29 in the Supporting Information.

### Stability of Potential Ligands and Metal Complexes in Aqueous Solutions

The stability of ligands **HL**^**2**^ and **HL**^**3**^ in aqueous solution containing 1% DMSO over 24 and 48 h was monitored by UV–vis spectroscopy (Figure S30 in the Supporting Information). Similarly, the behavior of complexes **1a** and **1b** as well as **2** and **3** was investigated in aqueous solutions containing 1% DMSO over 12 and 48 h by optical spectroscopy (Figures S31 and S32). All compounds studied remained intact as no changes in the UV–vis absorption spectra over 48 h were observed. High thermodynamic and kinetic stability of five-coordinate complexes reported herein can presumably be explained in terms of hard and soft acids and bases theory. Cu^2+^ as a borderline metal ion is expected to bind to a soft base, for example, S atom instead of terminal N atom, as is the case for thiosemicarbazides. However, the hard–soft character of the metal ion might be altered by the other ligands attached due to symbiotic effects.^30^ This might be the case herein, where bonding of copper(II) to four hard base ligands, namely, to two N atoms (N7 and N15 in Figure 1) and two Cl^–^ co-ligands, as well as to one borderline base N(pyridine) atom, can reduce the softness of Cu^2+^, leading to increased stability of the complexes.

In addition to UV–vis data, the stability and purity of ligand **HL**^**5**^ and complex **5** were tested by analytical HPLC-HR ESI MS using methanol or acetonitrile with 0.1% formic acid as the eluent over 10 min. A single peak at around 1 min corresponding to [**HL**^**5**^ + H]^+^ (found *m*/*z* = 460.0966 (Figure S27), calcd *m*/*z* for C_24_H_20_BrN_5_ 460.0975) as well as to [Cu^II^(**HL**^**5**^)]^+^ (found *m*/*z* = 521.0084 (Figure S28), calcd *m*/*z* for C_24_H_19_BrCuN_5_ 521.0097) was registered in agreement with other experiments. Moreover, the ^1^H NMR spectra of **HL**^**5**^ in 2:1 DMSO-*d*_6_/D_2_O at 25 °C measured immediately after dissolution, 2 h later, and after 20 h did not show any changes attesting their stability in aqueous DMSO solution (Figure S33).

### Antiproliferative Activity

The cytotoxicity of metal-free indolobenzazocines **HL**^**1**^–**HL**^**5**^ and their metal complexes **1a**, **1b**, and **2**–**5** was investigated by the colorimetric MTT assay in a panel of four human cancer cell lines, namely, human colon carcinoma HCT116 and LS-174, breast adenocarcinoma MDA-MB-361, and lung adenocarcinoma A549, as well as in human non-malignant cell line MRC-5 maintained as monolayer culture. Cisplatin or *cis*-diamminedichloridoplatinum(II) (CDDP), a well-known chemotherapeutic agent, was used as a positive control. The results obtained after 72 h of continuous drug action are presented as IC_50_ values (μM) in Table 1.

**Table 1:** IC_50_ Concentrations (μM) for Tested Compounds Obtained after 72 h of Continuous Drug Action^a^

	IC_50_ (μM) ± S.D.				
compound	HCT116	LS-174	MDA-MB-361	A549	MRC-5
**HL**^**1**^	149.1 ± 0.4	190.8 ± 5.8	161.6 ± 4.1	>200	168.1 ± 15.8
**HL**^**2**^	5.7 ± 0.4	8.9 ± 0.7	4.7 ± 0.3	7.6 ± 0.9	5.9 ± 0.8
**HL**^**3**^	3.2 ± 0.6	9.7 ± 2.2	2.5 ± 0.5	4.4 ± 0.6	2.2 ± 0.7
**HL**^**4**^	2.9 ± 0.6	4.0 ± 0.2	2.2 ± 0.0	4.9 ± 1.6	2.4 ± 0.3
**HL**^**5**^	0.9 ± 0.2	3.6 ± 0.5	1.4 ± 0.4	3.2 ± 1.5	2.2 ± 0.5
**1a**	32.9 ± 7.1	>200	91.2 ± 3.5	159.5 ± 18.2	89.8 ± 2.1
**1b**	40.2 ± 6.7	182.3 ± 2.1	71.9 ± 2.6	91.0 ± 9.3	84.8 ± 6.6
**2**	9.1 ± 0.4	19.6 ± 0.4	6.8 ± 2.7	33.2 ± 6.0	12.5 ± 0.4
**3**	0.8 ± 0.1	3.9 ± 0.9	0.9 ± 0.1	2.8 ± 0.1	2.6 ± 0.5
**4**	6.6 ± 0.9	6.9 ± 1.0	5.5 ± 0.9	20.2 ± 0.1	3.4 ± 0.6
**5**	0.8 ± 0.1	2.1 ± 0.9	0.9 ± 0.0	1.9 ± 0.3	1.7 ± 0.3
A (Ru^II^)				5.2 ± 0.9^b^	
A (Os^II^)				9.2 ± 1.6^b^	
**B**				>80^c^	
**C**				0.35 ± 0.04^d^	
**D**				0.20 ± 0.03^e^	
**CDDP**	13.6 ± 1.1	19.0 ± 0.5	26.4 ± 3.7	17.4 ± 3.5	8.0 ± 1.1

a The results are quoted as average values (±SD) of three independent experiments, each consisting of three replicates, and sample means were compared to corresponding non-treated controls. > indicates that the IC_50_ value was not obtained in the tested range of concentrations.

b Data taken from ref (31).

c Data taken from ref (32) (IC_50_ value is the same for both ruthenium and osmium complexes; a further increase of concentration was limited by low solubility of the compounds), with an exposure time of 96 h.

d Data taken from ref (17).

e Data taken from ref (16); S.D. = standard deviation.

**Chart 3:**
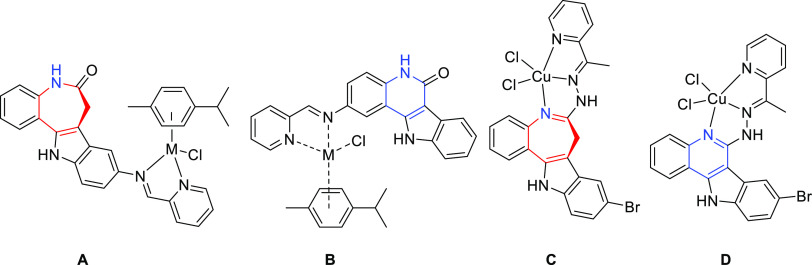
Ruthenium(II) and osmium(II) complexes with indolo[3,2-*d*]benzazepine ligand (**A**) and indolo[3,2-*c*]quinoline ligand (**B**) (where M = Ru, Os) as well as copper(II) complexes with a paullone ligand (**C**) and with indolo[3,2-*c*]quinoline ligand (**D**), reported previously.^16,17,31,32^

For comparison, the IC_50_ values for related complexes, in which the eight-membered azocine ring is replaced by a seven-membered azepine ring (**A** and **C**) or by a six-membered pyridine ring (**B** and **D**) (Chart 3) in A549 cells, are also included in Table 1.

**Figure 2:**
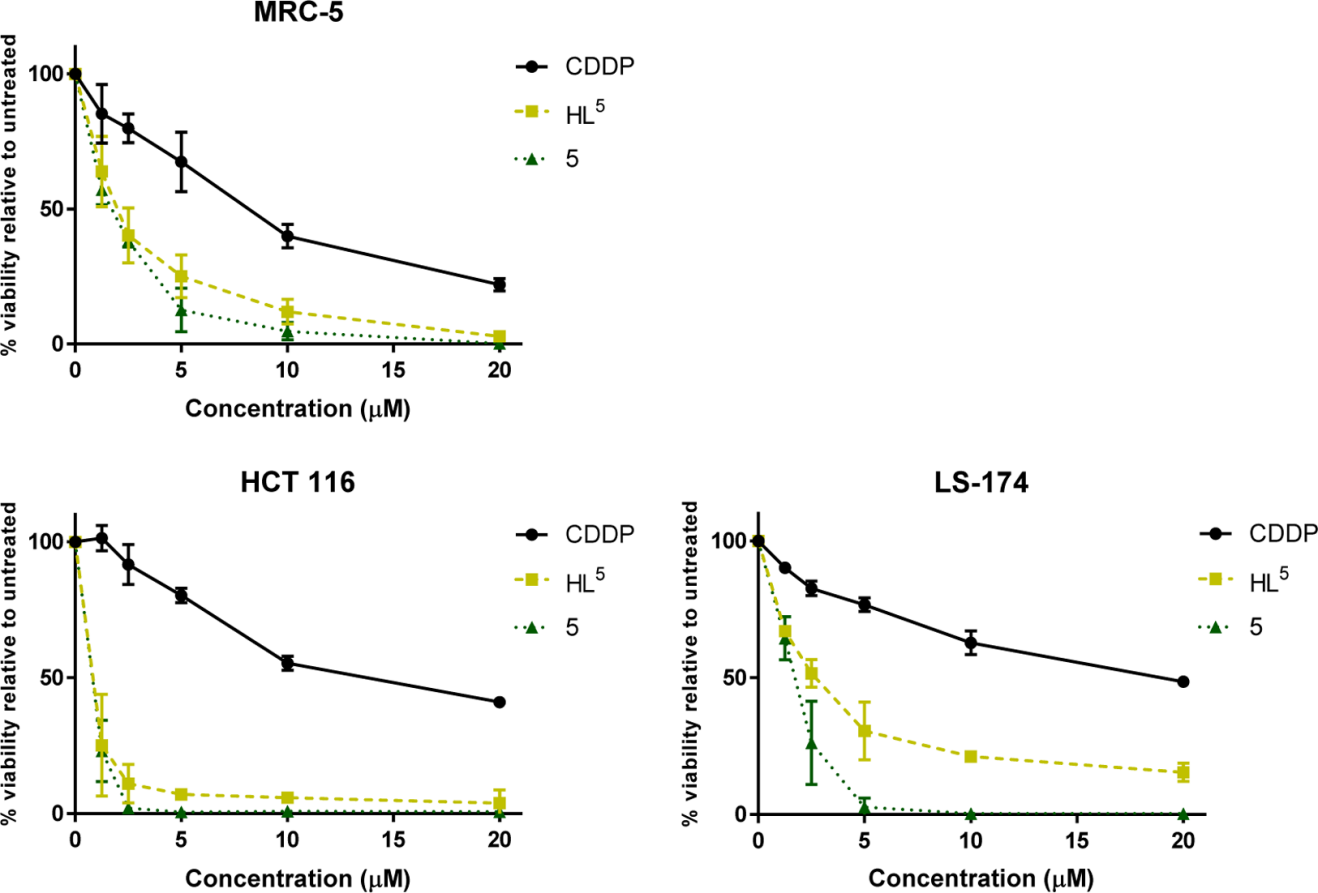
Representative cell survival curves obtained in cell lines MRC-5, HCT116, and LS-174 after incubation with CDDP, **HL**^**5**^, and **5** for 72 h.

The data collected in Table 1 indicate that indolobenzazocines **HL**^**2**^**–HL**^**5**^ show high cytotoxic activity with IC_50_ values in the low micromolar range and are significantly more efficient than **HL**^**1**^ and the reference chemotherapeutic drug cisplatin. Modification of the original indolobenzazocine scaffold at the lactam moiety has a huge favorable effect on antiproliferative activity compared to modification performed at position 10 of indole moiety, a structure–activity relationship also noticed for paullones.^33^ Coordination of **HL**^**1**^ to ruthenium(II)-arene and osmium(II)-arene as well as **HL**^**3**^ and **HL**^**5**^ to copper(II) enhanced their cytotoxicity, while binding of **HL**^**2**^ and **HL**^**4**^ to copper(II) did not result in an increase of their antiproliferative activity. As can be seen from Table 1, the most active were complexes **3** and **5**, both showing IC_50_ values in the low micromolar concentration range. Importantly, **5** was more selective for HCT116 cells than for non-tumor MRC-5 cells (selectivity index 2.1). Cell survival diagrams for the two lead drug candidates **3** and **5** are shown in Figure 2. Both exhibited significantly higher antiproliferative activity compared to cisplatin in all tested tumor cells. In addition, they showed a significant effect in two multidrug-resistant cell lines (LS-174 and A549).

In terms of selectivity for tumor cell lines, HCT116 (colorectal carcinoma) was the most chemosensitive line. The cytotoxic potential of ligands **HL**^**1**^**–HL**^**5**^ in HCT116 cells follows the order **HL**^**5**^ > **HL**^**4**^ > **HL**^**3**^ > **HL**^**2**^ > **HL**^**1**^, which closely correlates to the order of cytotoxic activity of the metal complexes: **5** ∼ **3** > **4** > **2** > **1b** > **1a**. Analysis of structure–activity relationships indicates that a methyl substituent at the Schiff base C=N group (R_2_ = CH_3_) highly contributes to enhancement of antiproliferative activity of ligands **HL**^**3**^ and **HL**^**5**^ and the corresponding complexes **3** and **5**, while the presence of bromide R_1_ = Br at indole moiety at position 10 (R_1_ = Br in Scheme 1, see also Figure 1) had a smaller but favorable effect on the biological activity of both **HL**^**5**^ and **5**.

In addition, comparison with ruthenium and osmium complexes with a related paullone (Chart 3, **A** and Table 1) shows that replacement of eight-membered azocine ring by seven-membered azepine has a strongly favorable effect on antiproliferative activity, irrespective of the metal ion.^31^ On the other hand, Ru(II) and Os(II) complexes with the indoloquinoline derivative bearing the binding site at quinoline moiety (Chart 3, **B**) exhibited weak antiproliferative activity comparable to that of **1a** and **1b**.^32^

According to the results of the MTT assay, complexes **3** and **5** were the most active. Taking into account slight superiority of **5** over **3** in cancer cell lines LS-174 and A549, the former was chosen for further biological studies, along with **HL**^**5**^, which was selected for comparison reasons.

### Cell Cycle Analysis by Flow Cytometry

To determine whether the suppression of cancer cell growth by investigated agents was associated with a cell cycle arrest, flow cytometry analysis of the DNA content was performed in HCT116 and LS-174 cells by propidium iodide (PI) staining. Cells were treated with IC_50_ and 3× IC_50_ concentrations of **HL**^**5**^ and **5** or 10 μM cisplatin for 24 and 48 h.

**Figure 3:**
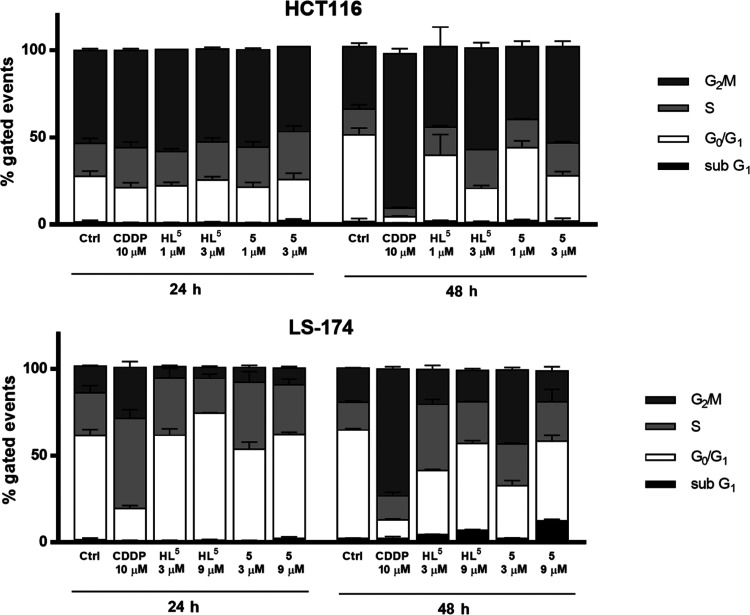
Diagrams presenting cell cycle phase distribution of treated HCT116 and LS-174 cells, obtained by flow-cytometric analysis of the DNA content in fixed cells, after staining with PI. Cells were collected following 24 and 48 h treatment with **HL**^**5**^ and **5** at concentrations corresponding to IC_50_ and 3× IC_50_. Bar graphs represent mean ± SD in at least two independent experiments. CDDP at 10 μM was used as a reference compound.

Both compounds **HL**^**5**^ and **5** showed dose- and time-dependent effects on cell cycle progression in HCT116 and LS-174 cells. As shown in Figure 3, upon exposure of the HCT116 cells to **HL**^**5**^ and **5**, cell cycle phase distribution has not considerably changed over the first 24 h, when compared to the non-treated cell population. However, after prolonged 48 h action, ligand **HL**^**5**^ and complex **5** showed a similar trend causing subtle dose-dependent arrest in both S and G2M phases, while sub-G1 peak, which is considered as hallmark of internucleosomal DNA cleavage,^34,35^ was not detected.

Analysis in colon carcinoma LS-174 cells revealed that both ligand **HL**^**5**^ and complex **5** showed time- and concentration-dependent perturbations of cell cycle, which at a lower time point (24 h) were characterized by transient arrest in the S phase, indicating stalled DNA replication. With prolonged treatment (48 h), ligand **HL**^**5**^ and complex **5** induced further perturbations of cell cycle with an increase of sub-G1 population, reaching up to 12.31% of all events. We may conclude that different cell cycle perturbations following action of **HL**^**5**^ and **5** in colon carcinoma HCT116 and LS-174 cells are induced due to the different kinetics and different modes of cell death induction.

Cisplatin as a reference compound and a typical DNA-damaging drug impaired progression in the S phase at 24 h time point, followed by G2M arrest at 48 h, as the result of the formation of cisplatin DNA adducts and blockage of DNA replication.^36−38^

### Annexin V-FITC Apoptosis Assay

**Figure 4:**
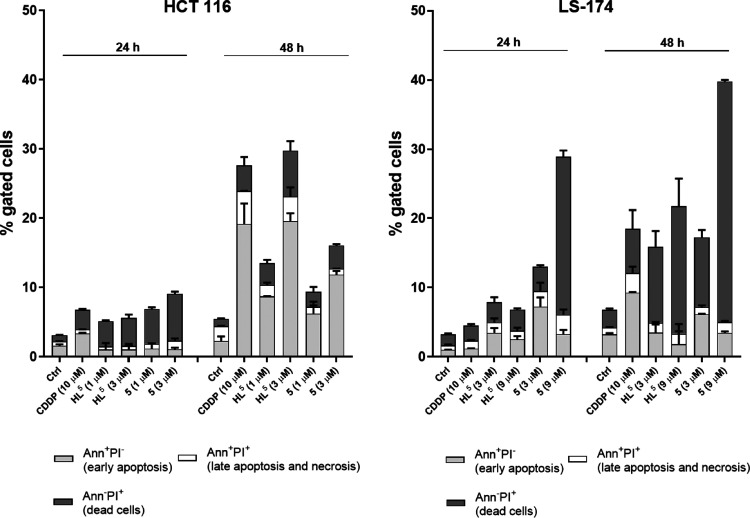
Apoptosis and necrosis were quantified by FACS after Annexin V-FITC and PI labeling after 24 and 48 h of treatment; bar graphs represent mean ± SD in at least two independent experiments.

The potential of **HL**^**5**^ and **5** to induce apoptosis was analyzed after 24 or 48 h of treatment with IC_50_ and 3× IC_50_ concentrations by flow cytometry, following Annexin V-FITC/propidium iodide dual staining and compared to that of cisplatin. The obtained experimental data are presented in Figure 4 as percentages of early apoptotic cells (Annexin V-positive/PI-negative staining), late apoptotic and necrotic cells (Annexin V-positive/PI-positive staining), and dead cells (Annexin V-negative/PI-positive staining). By the current test (BD Pharmingen protocol)^39^ for the cells which are already dead (Annexin V-negative/PI-positive staining), we cannot distinguish between types of the occurred cell death. Dot plot diagrams are shown in Figure S34.

In HCT116 cells, after 24 h of treatment, only cisplatin induced a small increase in percentage of early apoptotic cells. Prolonged incubation (48 h) led to an exponential increase of the number of the early apoptotic cells (Annexin V-positive/PI-negative staining). Early apoptosis staining at the highest concentrations of agents (3× IC_50_) reached 19.14% in cisplatin-treated cells, 19.58% in **HL**^**5**^-treated cells, and 11.83% in cells treated with complex **5** versus 2.29% of the non-treated control. A lower number of late apoptotic/necrotic (Annexin V-positive/PI-positive staining) or dead cells (Annexin V-negative/PI-positive staining) was detected. This result was in line with the perturbations of cell cycle occurred after prolonged 48 h action of both complex **5** and **HL**^**5**^ in HCT116, where sub-G1 peak, which is a marker of DNA cleavage, an event characteristic for late apoptotic (or even necrotic) changes,^35,38,40^ was not detected. Apoptosis is a dynamic and kinetic event that can be affected by the cell type, apoptotic inducer, and cell cycle. It is also of note that the Annexin V-positive/PI-negative staining occurs in the early phase of apoptosis, when DNA fragments are not yet detected.^35,38,40^

In LS-174 cells after 24 h of treatment, complex **5** showed dose-dependent behavior and induced apoptosis at IC_50_ (up to 7.26 vs 1.01% for control cells) as well as considerable cell death at a higher dose, 3× IC_50_ (up to 22.86 vs 1.63% in control cells). After prolonged treatment (48 h) with **HL**^**5**^ or **5**, as presented in Figure 4, a substantial increase of the number of dead cells with a disturbed cell membrane (Annexin V-negative/PI-positive) was observed. However, we cannot distinguish dead cells that have undergone apoptotic death from those that have died as a result of a necrotic pathway.^39^ At the highest concentrations of agents (3× IC_50_), both **HL**^**5**^ and **5** increased the number of dead cells up to 18.46 and 34.79%, respectively, when compared with 2.5% in control cells. The data obtained are in agreement with the cell cycle changes in Figure 3, which show occurrence of sub-G1 DNA fragmentation after 48 h as an event in the late apoptotic (or even necrotic) pathway.^35,38,40^

### Cell Morphological Study

**Figure 5:**
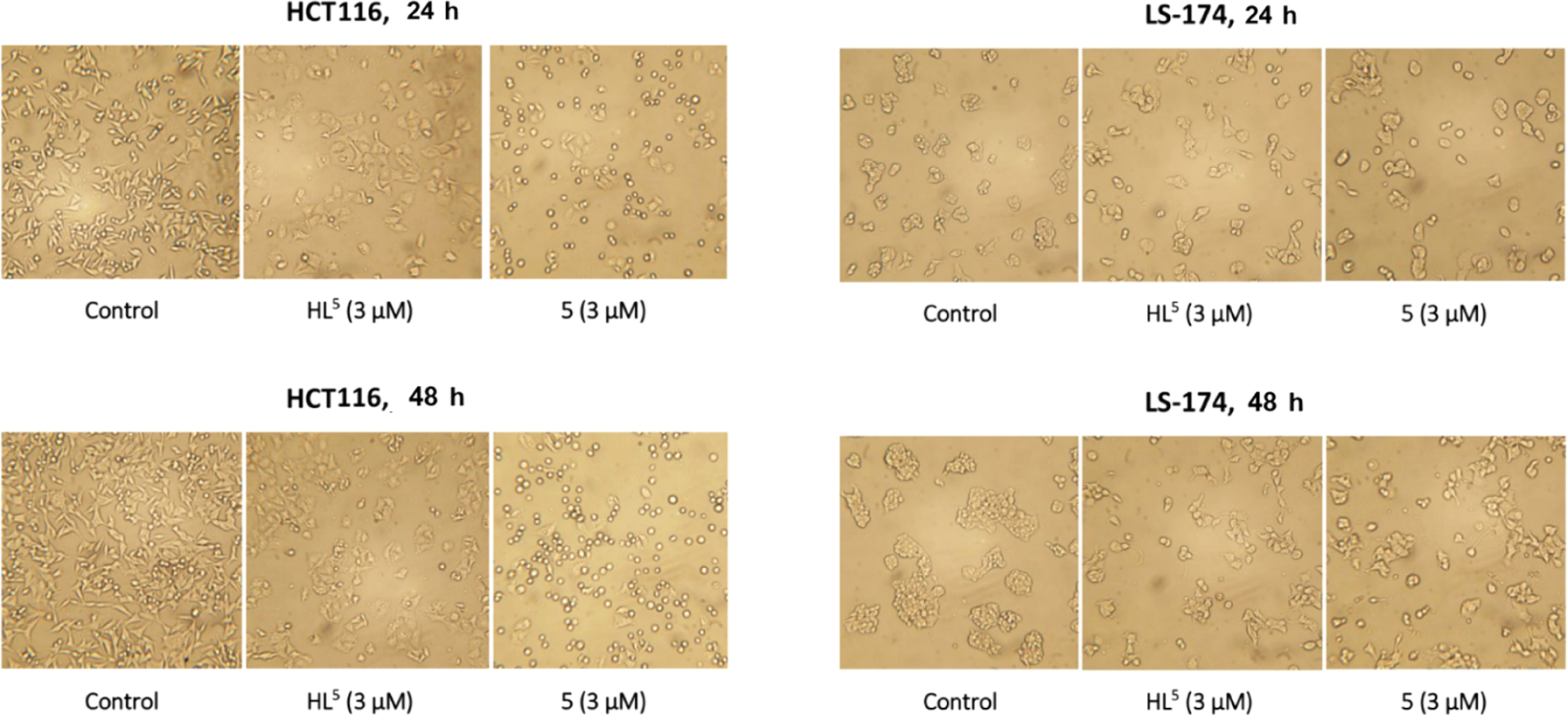
Micrographs presenting HCT116 and LS-174 cells treated with ligand **HL**^**5**^ and complex **5** after 24 and 48 h of treatment. Untreated cells were used as control cells. Bright-field images were obtained using an inverted microscope.

Morphological changes in cell size and shape were investigated under the bright field microscope and presented in Figure 5. HCT116 cells started to lose their normal morphology after 24 h of treatment. Both cells treated with ligand **HL**^**5**^ and complex **5** were reduced in number and became larger in size, while a majority of cells treated with complex **5** became rounded. These alterations were even more pronounced following 48 h of treatment. Similar changes have been observed in LS-174 cells. After 48 h of treatment, a mixed cell population was present with the appearance of enlarged individual cells with long pseudopods, compared with control LS-174 cells that normally grow in islands. These observations are compatible with our results of the cell cycle analysis which showed the potential of tested compounds to affect cell division and induce cell cycle arrest, resulting in enlarged cells. Moreover, the presence of floating rounded and irregularly shaped cells indicates that ligand **HL**^**5**^ and complex **5** caused disruption of molecular mechanisms leading to cell death, which were demonstrated in apoptosis study by Annexin V-FITC binding.

### Antiproliferative Activity of the Investigated Agents in 3D Multicellular Tumor Spheroid Model

Due to the complex tissue environment, 3D multicellular tumor spheroid (MCTS) models mimic the in vivo architecture closer than 2D cell cultures, thereby allowing more precise prediction of effectiveness of organic drug candidates as well as metal-based ones in animal models.^41−44^ The efficacy of **HL**^**5**^, **5**, and cisplatin in the 3D MCTS model was investigated in two human colorectal cancer cell lines (CRC) LS-174 and HCT116. We used ultra-low attachment (ULA) plates for the growth of spheroids. Spheroids were incubated for 72 h and then photographed, and IC_50_ values were determined using MTT assay. Tumor spheroids of diameter >500 μm selected for treatment reflect to a certain extent the tumor complexity as they are composed of several specialized areas and layers where cells show different phenotypic, functional, and metabolic behaviors.^45^ They display an organized architecture with an external layer composed of proliferating cells, an intermediate zone composed of quiescent and senescent cells, and an inner apoptotic and necrotic core which is the result of the reduced distribution of nutrients and oxygen in these areas.^46,47^

Analysis of growth inhibition images after 72 h of drug treatment of HCT116 cells with different drug concentrations showed that **HL**^**5**^ induced growth inhibition of spheroids in a concentration-dependent manner. Complex **5** also induced growth inhibition with its apparent effect at 5 μM concentration, while higher concentrations of 10 and 20 μM seem to induce weakening of contacts between cells and loss of compactness of spheroids. The architecture of spheroids is lost as the external proliferative layer of cells (rim) disappears and the number of dying cells increases. Treatment with CDDP induced growth inhibition of spheroids in a concentration-dependent manner with an effect similar to complex **5**, characterized by losing the spheroid architecture and decay of an external proliferative layer (rim) at higher concentrations up to 20 μM. Analysis of LS-174 spheroids after treatment did not show evident inhibition of growth/size of spheroids as was the case with HCT116 cells but showed loss of architecture of spheroids in terms of compactness decay of the external proliferative layer at higher drug concentrations.

**Figure 6:**
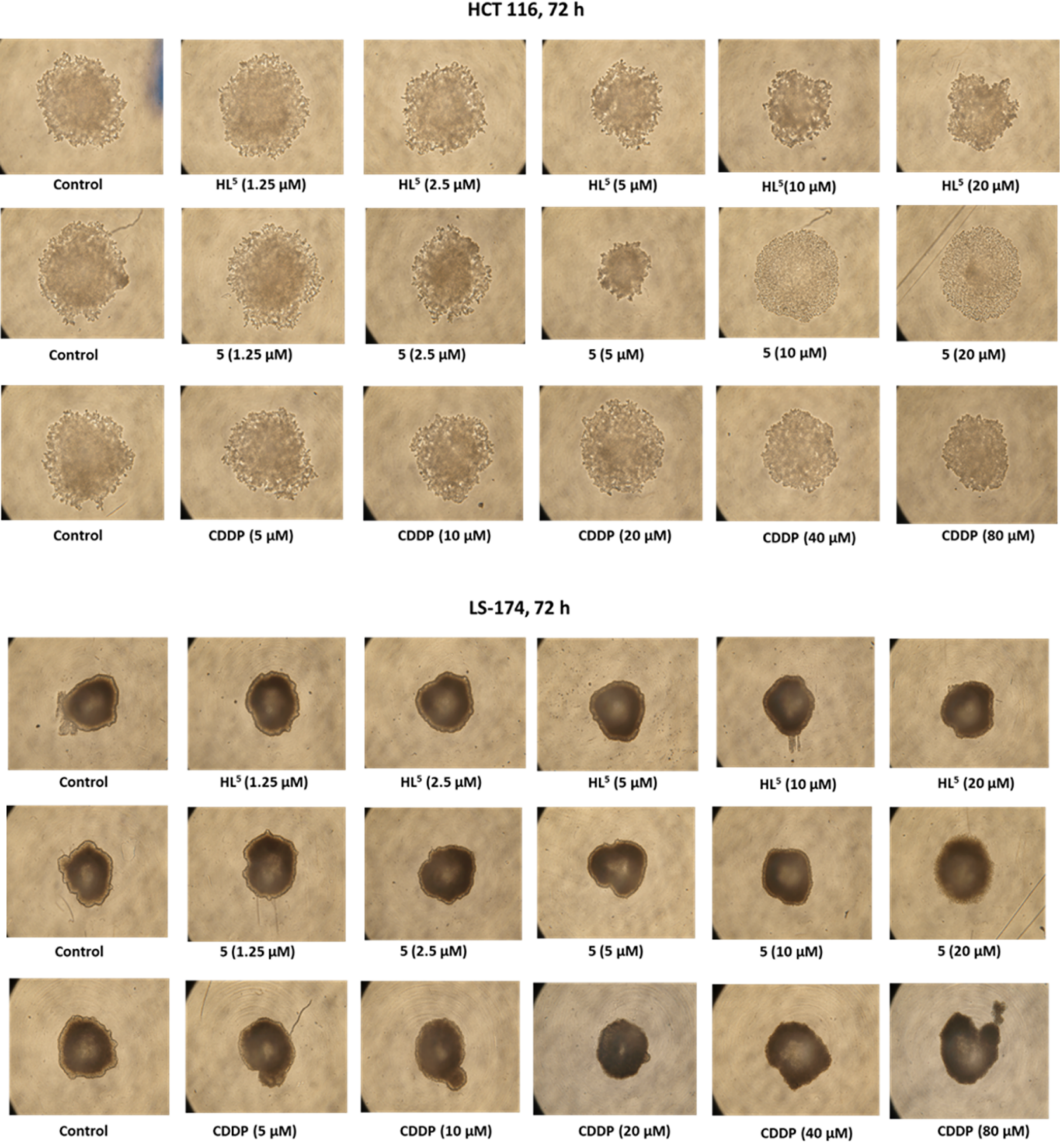
Growth inhibition images obtained after 72 h of drug treatment in HCT116 and LS-174 MCTS. The HCT116 (500 c/w) and LS-174 (1500 c/w) cells were seeded into a low-attachment U96-well plate Thermo Scientific Nunclon Sphera. After 4 days of culture (spheroidization time), the MCTSs pre-selected for a homogeneous volume and shape were treated with **HL**^**5**^, **5**, and CDDP. Bright-field images were obtained using an inverted microscope.

In agreement with the images obtained (Figure 6), IC_50_ values determined by MTT assay in the 3D culture model of MCTS presented in Table 2 revealed a lower cytotoxic effect of **HL**^**5**^ and **5** than obtained in the 2D model. However, the important level of activity, higher than the activity of cisplatin, being below 10 μM for **5** was determined on both cell lines. In HCT116 cells, **HL**^**5**^ showed approximately 10× higher IC_50_ value in 3D versus 2D model, with IC_50_ values being 8.80 and 0.9 μM, respectively. Complex **5** showed approximately 3× higher IC_50_ value in 3D versus 2D model in HCT116 cells, with IC_50_ being 2.28 μM and 0.8 μM, respectively. These results indicate that complex **5** retained its cytotoxic potential in the 3D models of colorectal cancer more than its corresponding ligand **HL**^**5**^. In LS-174 cells, ligand **HL**^**5**^ showed an IC_50_ value over 20 μM in the 3D model and IC_50_ = 3.6 μM in the 2D model. In turn, complex **5** showed 4× higher IC_50_ value in 3D versus 2D model, with IC_50_ being 9.3 μM and 2.1 μM, respectively. Cisplatin maintained nearly the same cytotoxic potential in the monolayer or MCTS model in both cell lines, with IC_50_ values being in accordance with the literature data.^34,48^ Complex **5** exhibited superior cytotoxic activity compared to cisplatin in both cell models. We may conclude that HCT116 cells were particularly sensitive to the action of complex **5** in both cell models. MCTS represents a valuable tool for in vitro drug investigation as an extrapolation to conditions in vivo (such as gradient of nutrients and oxygen, cell–cell and cell–extracellular matrix interactions, etc.), which affect drug efficiency and determine tumor cell susceptibility/resistance to drug action.^45^

**Table 2:** IC_50_ (μM) Values^a^ for **HL**^**5**^, Its Corresponding Copper(II) Complex **5**, and **CDDP** in CRC Cells Determined in 3D Cell Culture Models by MTT Assay for 72 h Continuous Drug Action

	IC_50_ (μM) ± S.D.	
compound	HCT116	LS-174
**HL**^**5**^	8.8 ± 0.7	>20
**5**	2.3 ± 0.2	9.3 ± 2.2
**CDDP**	19.6 ± 2.2	17.8 ± 1.2

a Results are presented as IC_50_ values and represent average values obtained from two to three independent experiments with their standard deviations. S.D. = standard deviation.

### Enzyme Inhibition

Paullones were first reported as ATP-competitive CDK1, CDK2, CDK5, and GSK-3β inhibitors.^49,50^ In vitro assays in a panel of 28 enzymes performed several years later^51^ confirmed the selectivity of Kenpaullone to GSK-3β and CDK2 with IC_50_ values 0.23 and 0.67 μM, respectively, but, in addition, Lck, a member of Src family of protein kinases, was inhibited with similar potency (IC_50_ 0.47 μM for both Kenpaullone and Alsterpaullone). A further update reported in 2007 for a panel of 80 kinases provided further evidence of high selectivity of Alsterpaullone and Kenpaullone for GSK-3β and CDK2.^52^ Taking into account all these data, the ability of **HL**^**5**^ and **5** to disturb cell cycle progression, and the close similarity of core structures in paullones and in our current lead species **HL**^**5**^ and **5**, we decided to test the inhibitory potency of the latter two compounds against 7 particular enzymes from 50 currently available at the Kinase Centre of the University of Dundee, namely, CDK2, CDK5 and CDK9, GSK-3α, GSK-3β, Lck, and Src. It is also worth noting that the recently reported indolo[2,3-*c*]quinoline-derived compound and its copper(II) complex,^53^ whose main organic scaffold differs from that in **HL**^**5**^ and **5** more significantly, namely, by the flip of indole moiety and planarity of the core structure, showed a quite different kinase inhibitory pattern. The lead organic compound revealed good potency against PIM-1, while the copper(II) complex showed significant inhibition of the activity of SGK-1, PKA, CaMK-1, GSK-3β, and MSK1 from a panel of 50 kinases.

By using a cell-free radioactive filter binding assay, the inhibitory activity of the two lead drug candidates was assessed, and the data are summarized in Table 3 and Figure S35. Both **HL**^**5**^ and **5** effectively inhibited all tested kinases, but Cdk9, with IC_50_ values in the low micromolar concentration range. Generally, complex **5** revealed 2 to 4× higher efficacy of inhibition than metal-free ligand **HL**^**5**^. Complex **5** demonstrated the most effective inhibition against GSK-3α and Lck. The activity of novel agents against several kinases can be both an advantage and a disadvantage, as discussed in more details in the review articles.^54−56^ Briefly, to avoid unpredictable toxic effects, research efforts are focused on design of highly selective inhibitors, but tumor cell survival and progression is a multifactorial process, sustained by a complex network of protein kinases and cross-talk among different signaling pathways, so it seems reasonable to establish anticancer therapies that target several kinases associated with tumor growth. Overall, the collected data suggest that **HL**^**5**^ and **5** are potentially multi-kinase inhibitors.

**Table 3:** Inhibition Activity of **HL**^**5**^ and **5** against a Panel of the Protein Kinases

	Mean IC_50_ ± SD (μM)	
protein kinase	**HL**^**5**^	**5**
GSK-3α	3.5 ± 0.7	1.4 ± 0.1
GSK-3β	5.0 ± 1.2	5.4 ± 0.2
Src	5.9 ± 0.1	2.5 ± 0.4
Lck	4.8 ± 1.9	1.2 ± 0.3
Cdk2	6.1 ± 1.3	3.9 ± 0.2
Cdk5	4.5 ± 0.2	2.7 ± 0.4
Cdk9	>1000	>1000

To find out the physiological role(s) of GSK-3α and Lck in cell-based assays, the same effect should be observed with at least two structurally unrelated inhibitors of these protein kinases.^57^ In accord with the results reported previously,^51^ the combined use of **5** and LiCl may help in identifying the substrates and physiological roles of GSK-3α in cells. This kind of investigations is imperative and will be performed in the future.

## Conclusions

In summary, in addition to bioactive ring systems documented in the literature, such as indolo[3,2-*c*]quinolines and indolo[3,2-*d*][1]benzazepines and indolo[3,2-*d*][2]benzazepines incorporating either a six- or seven-membered N-containing ring, respectively, an entry to indolo[3,2-*e*]benzazocine scaffold containing an eight-membered azocine ring has been realized. Five bi- and tridentate ligands (**HL**^**1**^**–HL**^**5**^) and ruthenium(II) (**1a**), osmium(II) (**1b**), and copper(II) complexes (**2–5**) have been synthesized and characterized. In vitro cytotoxicity tests against four cancer cell lines (LS-174, HCT116, MDA-MB-361, and A549) revealed ligand **HL**^**5**^ and copper(II) complex **5** as series leaders, with a strong cytostatic effect higher than that of cisplatin (particularly against human colorectal cancer cell lines HCT116 and LS-174). These compounds were also more selective for HCT116 cells than for normal human lung fibroblasts (MRC-5). Morphological studies in the presence of **HL**^**5**^ and **5** showed disturbance of cell growth over time. In addition, cell cycle analysis revealed that both **HL**^**5**^ and **5** induce concentration- and time-dependent arrest of cell cycle phases, which differs from that for cisplatin. Annexin V-FITC apoptosis assay showed dominant apoptosis inducing potential after prolonged treatment (48 h) in HCT116 cells.

**HL**^**5**^ and **5** can inhibit Cdks, as further supported by cell-free radioactive filter binding assay against Cdk2 and Cdk5. However, human cells integrate mitogen and stress stimuli before committing to the cell cycle by regulating the levels of different Cdks, which also play roles in transcriptional processes and apoptosis programs. Thus, due to the complex and multiple roles of Cdks in cell signaling, additional studies are needed to precisely address the molecular mechanism underlying the Cdk-inhibitory and cytotoxic action of the tested compounds. In general, Cdk protein kinases share structural and functional similarities, and development of the small molecule Cdk inhibitors with distinct specificity is an extremely challenging task.^58,59^

As for paullones, GSK-3α, GSK-3β, Src, and Lck are among other possible targets for lead drug candidates **HL**^**5**^ and **5**. Complex **5** showed higher antiproliferative activity in the 3D culture model of MCTS than clinical drug cisplatin, attesting its suitability for in vivo assays. Thus, these findings indicate that incorporation of an eight-membered azocine ring into related paullone structures enriches the available chemical space of bioactive scaffolds, and, in combination with substitution at the lactam unit, Schiff base C=N bond and bromination at position 10 are effective tools for fine-tuning the biological potency of copper(II)-based anticancer drugs.

## Experimental Section

### General Information

NMR spectra were recorded on Bruker AV700, AV600, or AV500 spectrometers. ^1^H and ^13^C NMR chemical shifts (δ) are given in ppm relative to TMS using the residual solvent signals as references and converting the chemical shifts to the TMS scale. ESI-MS spectra were recorded on a Bruker amaZon speed ETD spectrometer (3D-ion trap). High-resolution ESI mass spectra were recorded on a Bruker maXis UHR-TOF spectrometer.

### Materials

5-Nitro-1*H*-indole-3-carboxaldehyde, 2-iodobenzonitrile, and silver(I) carbonate were purchased from abcr. 1-Ethyl-3-(3-dimethylaminopropyl)carbodiimide hydrochloride (EDCI·HCl) and trifluoroacetic acid were purchased from IRIS biotech, while palladium (10%) on activated charcoal, *tert*-butyl dicarbonate (Boc_2_O), palladium(II) acetate, 4-(dimethylamino)pyridine (DMAP), sodium chlorite, and sulfamic acid were obtained from Sigma-Aldrich. 2-Formylpyridine, absolute dimethylformamide (DMF), tetra*-n*-butylammonium fluoride (TBAF), 4-toluenesulfonyl chloride, triethylamine, sodium bicarbonate, magnesium sulfate, and borane solution (1 M in THF) were bought from Fisher/Acros Organics. Triphenylphosphine, sodium bicarbonate, and celite were purchased from Alfa Aesar. 2-Iodobenzeneethylamine was prepared by using a literature protolol.^28^

Synthesis of the main organic scaffolds **b**_**1**_–**h**_**3**_ is described in detail in the Supporting Information. ^1^H NMR spectra of intermediate species **b**_**1**_, **b**_**2**_, **c**_**1**_**–c**_**3**_, **d**_**1**_**–d**_**3**_, **e**_**1**_**–e**_**3**_, **f**_**1**_**–f**_**3**_, **g**_**1**_**–g**_**3**_, **h**_**1**_, and **h**_**2**_ are shown in Figures S36–S54.

### Synthesis of Ligands **HL**^**1**^**–HL**^**5**^

**HL**^**1**^**·0.5CH**_**3**_**OH**: To a solution of **g**_**1**_ (520 mg, 1.88 mmol) in anoxic ethanol (80 mL) in a 250 mL Schlenk tube, 2-formylpyridine (196 μL, 2.06 mmol) was added, and the mixture was stirred at 85 °C for 20 h. On the next day, the reaction mixture was cooled to room temperature and the solvent was evaporated under reduced pressure. The product was crystallized in methanol and isolated as a yellow powder. Yield: 380 mg, 55%. ^1^H NMR (600 MHz, DMSO-*d*_6_): δ, ppm: 11.84 (s, 1H, H^13^), 8.72 (dd, *J* = 4.8, 0.6 Hz, 1H H^18^), 8.68 (s, 1H H^15^), 8.20 (d, *J* = 7.9 Hz, 1H, H^21^), 7.96 (td, *J* = 7.5, 1.3 Hz, 1H, H^20^), 7.69 (d, *J* = 2.0 Hz, 1H, H^9^), 7.51 (ddd, *J* = 7.5, 4.8, 1.1 Hz, 1H, H^19^), 7.48 (d, *J* = 8.6 Hz, 1H, H^12^), 7.46–7.41 (m, 3H, H^2,3,4^), 7.41–7.37 (m, 1H, H^1^), 7.35 (t, *J* = 4.8 Hz, 1H, H^7^), 7.32 (dd, *J* = 8.6, 2.1 Hz, 1H, H^11^), 3.52–3.47 (m, 2H, H^6^), 3.06 (t, *J* = 6.8 Hz, 2H, H^5^). ^13^C{H} NMR (176 MHz, DMSO-*d*_6_): δ, ppm: 169.09 (Cq, C^8^), 157.81 (CH, C^15^), 154.52 (Cq, C^16^), 149.64, (CH, C^18^) 143.43 (Cq, C^10^), 138.11 (Cq, C^4a^), 137.71 (Cq, C^13b^), 136.99 (CH, C^20^), 135.70 (Cq, C^12a^), 131.66 (Cq, C^13a^), 130.55 (CH, C^4^), 130.41 (CH, C^2^), 128.89 (CH, C^3^), 128.18 (Cq, C^8b^), 126.30 (CH, C^1^), 125.20 (CH, C^19^), 120.84 (CH, C^21^), 116.68 (CH, C^11^), 112.90 (CH, C^9^), 112.11 (CH, C^12^), 110.36 (Cq, C^8a^), 43.65 (CH_2_, C^6^), 34.46 (CH_2_, C^5^). Solubility in water/1% DMSO ≥ 1.0 mg mL^–1^. Anal. Calcd for C_23_H_18_N_4_O·0.5CH_3_OH (*M*_r_ 382.44), %: C, 73.80; H, 5.27, N, 14.65. Found, %: C, 73,79; H, 5.27; N, 14.66. IR spectrum (selected bands, ATR, ν_max_, cm^–1^): 3344.44 (w), 1595.89 (m), 1484.18 (s), 1438.50 (s), 1397.89 (m), 1334.86 (m), 1269.23 (w), 1223.41 (w), 1166.81 (w), 1107.81 (w), 1045.17 (w), 993.24 (w), 957.13 (w), 888.75 (w), 847.72 (w), 772.14 (m), 742.56 (s), 671.73 (w), 623.29 (w). HR (+)ESI-MS (acetonitrile/methanol + 1% water): *m*/*z* 389.1375 (see Figure S55); calcd *m*/*z* for [C_23_H_18_N_4_ONa]^+^ or [M + Na]^+^ 389.1373.

**HL**^**2**^: To a solution of **h**_**2**_ (379 mg, 1.31 mmol) in anoxic ethanol (13 mL) in a 25 mL Schlenk tube was added 2-formylpyridine (124 μL, 1.31 mmol), and the solution was stirred at 75 °C overnight. On the next day, the reaction mixture was cooled to room temperature and the yellow precipitate was filtered off. Yield: 345 mg, 72%. ^1^H NMR (600 MHz, DMSO-*d*_6_): δ, ppm: 11.79 (s, 1H, H^13^), 8.56 (d, *J* = 4.3 Hz, 1H, H^19^), 8.28 (d, *J* = 8.0 Hz, 1H, H^22^), 8.24 (s, 1H, H^16^), 7.86 (d, *J* = 7.9 Hz, 1H, H^9^), 7.81 (t, *J* = 7.1 Hz, 1H, H^21^), 7.51 (m, 1H, H^7^), 7.44 (d, *J* = 8.1 Hz, 1H, H^12^), 7.39 (td, *J* = 5.8, 1.7 Hz, 4H, H^1,2,3,4^), 7.34 (dd, *J* = 6.5, 5.1 Hz, 1H, H^20^), 7.18 (t, *J* = 7.2 Hz, 1H, H^11^), 7.10 (t, *J* = 7.4 Hz, 1H, H^10^), 3.59 (dd, *J* = 11.7, 6.5 Hz, 2H, H^6^), 3.07 (t, *J* = 7.0 Hz, 2H, H^5^). ^13^C{H} NMR (176 MHz, DMSO-*d*_6_): δ, ppm: 160.73 (Cq, C^8^), 154.63 (Cq, C^17^), 151.72 (Cq, C^16^), 149.24 (CH, C^19^), 138.49 (Cq, C^13a^), 137.85 (Cq, C^4a^), 136.45 (Cq, C^12a^), 136.21 (CH, C^21^), 132.26 (Cq, C^13b^), 130.51 (CH, C^3,4^), 128.72 (CH, C^2^), 127.06 (Cq, C^8b^), 126.13 (CH, C^1^), 123.64 (CH, C^20^), 121.99 (CH, C^11^), 121.28 (CH, C^9^), 120.67 (CH, C^22^), 119.96 (CH, C^10^), 111.37 (CH, C^12^), 107.67 (Cq, C^8a^), 43.87 (CH_2_, C_6_), 35.06 (CH_2_, C^5^). Solubility in water/1% DMSO ≥ 1.0 mg mL^–1^. Anal. Calcd for C_23_H_19_N_5_ (*M*_r_ 365.43), %: C, 75.58; H, 5.24, N, 19.17. Found, %: C, 75.22; H, 4.80; N, 18.66. IR spectrum (selected bands, ATR, ν_max_, cm^–1^): 3313.03 (w), 1594.76 (w), 1540.90 (m), 1500.07 (w), 1466.18 (w), 1446.73 (w), 1389.37 (s), 1314.43 (m), 1284.17 (m), 1247.74 (w), 1225.83 (w), 1149.32 (w), 1109.17 (w), 1025.60 (m), 994.20 (w), 937.16 (w), 917.96 (w), 841.14 (w), 769.25 (w), 741.87 (s), 662.72 (m). (+)ESI-MS (acetonitrile/methanol + 1% water): *m*/*z* 366.19; calcd *m*/*z* for [C_23_H_19_N_5_]^+^ or [M + H]^+^ 366.17.

**HL**^**3**^: To a solution of **h**_**2**_ (410 mg, 1.41 mmol) in anoxic ethanol (15 mL) in a 25 mL Schlenk tube was added 2-acetylpyridine (158 μL, 1.41 mmol), and the mixture was stirred at 75 °C overnight. On the next day, the reaction mixture was cooled to room temperature and concentrated under reduced pressure. The yellow product was precipitated by addition of 5 mL of diethyl ether and filtered off. Yield: 425 mg, 83%. ^1^H NMR (600 MHz, DMSO-*d*_6_): δ, ppm: 11.76 (s, 1H, H^13^), 8.56 (ddd, *J* = 4.8, 1.7, 0.9 Hz, 1H, H^19^), 8.40 (d, *J* = 8.1 Hz, 1H, H^22^), 7.96 (d, *J* = 7.9 Hz, 1H, H^9^), 7.80–7.73 (m, 1H, H^21^), 7.44 (d, *J* = 8.1 Hz, 1H, H^12^), 7.43–7.35 (m, 4H, H^1,2,3,4^), 7.33 (ddd, *J* = 7.4, 4.8, 1.1 Hz, 1H, H^20^), 7.28 (t, *J* = 4.7 Hz, 1H, H^7^), 7.19–7.15 (m, 1H, H^11^), 7.13–7.08 (m, 1H, H^10^), 3.58 (m, 2H, H^6^), 3.07 (t, *J* = 7.0 Hz, 2H, H^5^), 2.40 (s, 3H, H^23^). ^13^C{H} NMR (176 MHz, DMSO-*d*_6_): δ, ppm: 158.85 (Cq, C^8^), 157.44 (Cq, C^17^), 156.57 (Cq, C^16^), 148.39 (CH, C^19^), 138.22 (Cq, C^13a^), 137.97 (Cq, C^4a^), 136.49 (Cq, C^12a^), 135.86 (CH, C^21^), 132.46 (Cq, C^13b^), 130.39 (CH, C^3^),130.33 (CH, C^4^), 128.66 (CH, C^2^), 127.27 (Cq, C^8b^), 126.09 (CH, C^1^), 123.33 (CH, C^20^), 121.94 (CH, C^11^), 121.30 (CH, C^9^), 120.67 (CH, C^22^), 119.97 (CH, C^10^), 111.37 (CH, C^12^), 108.52 (Cq, C^8a^), 44.14 (CH_2_, C^6^), 34.94 (CH_2_, C^5^), 12.99 (CH_3_, C^23^). Solubility in water/1% DMSO ≥ 1.0 mg mL^–1^. Anal. Calcd for C_24_H_21_N_5_ (*M*_r_ 379.46), %: C, 75.97; H, 5.58, N, 18.46. Found, %: C, 75.52; H, 5.18; N, 17.98. IR spectrum (selected bands, ATR, ν_max_, cm^–1^): 1591.22 (w), 1553.04 (w), 1497.71 (w), 1462.99 (w), 1391.30 (m), 1361.60 (w), 1281.77 (w), 1251.59 (w), 1226.23 (w), 1153.79 (w), 1109.31 (w), 1038.24 (w), 994.58 (w), 958.55 (w), 910.33 (w), 840.81 (w), 809.72 (w), 778.16 (w), 741.35 (s), 672.65 (w), 623.24 (w). (+)ESI-MS (acetonitrile/methanol + 1% water): *m*/*z* 380.21; calcd *m*/*z* for [C_24_H_21_N_5_]^+^ or [M + H]^+^ 380.19.

**HL**^**4**^**·0.8H**_**2**_**O**: To a solution of **h**_**3**_ (160 mg, 0.45 mmol) in anoxic methanol (5 mL) in a 25 mL Schlenk tube was added 2-formylpyridine (43 μL, 0.45 mmol), and the mixture was stirred at 75 °C overnight. On the next day, the reaction mixture was cooled to room temperature and concentrated under reduced pressure. The yellow product was precipitated by addition of diethyl ether (5 mL) and filtered off. Yield: 171 mg, 85%. ^1^H NMR (600 MHz, DMSO-*d*_6_): δ, ppm: 12.03 (s, 1H, H^13^), 8.57 (m, 1H, H^19^), 8.28 (d, *J* = 8.0 Hz, 1H, H^22^), 8.26 (s, 1H, H^16^), 7.98 (d, *J* = 1.8 Hz, 1H, H^9^), 7.84–7.80 (m, 1H, H^21^), 7.51 (t, *J* = 4.7 Hz, 1H, H^7^), 7.43–7.40 (m, 4H, H^1,3,4,21^), 7.39–7.36 (m, 1H, H^2^), 7.35 (ddd, *J* = 7.5, 4.8, 1.2 Hz, 1H, H^20^), 7.31 (dd, *J* = 8.6, 2.0 Hz, 1H, H^11^), 3.58 (dd, *J* = 11.7, 6.9 Hz, 2H, H^6^), 3.10–3.01 (m, 2H, H^5^). ^13^C{H} NMR (176 MHz, DMSO-*d*_6_): δ, ppm: 160.16 (Cq, C^8^), 154.50 (Cq, C^17^), 152.14 (CH, C^16^), 149.26 (CH, C^19^), 139.90 (Cq, C^13a^), 137.92 (Cq, C^4a^), 136.22 (CH, C^21^), 135.14 (Cq, C^12a^), 131.69 (Cq, C^13b^), 130.49 (CH, C^3^), 130.45 (CH, C^4^), 129.03 (CH, C^1^), 128.73 (Cq, C^8b^), 126.21 (CH, C^2^), 124.52 (CH, C^11^), 123.73 (CH, C^20^), 123.29 (CH, C^9^), 120.75 (CH, C^22^), 113.48 (CH, C^12^), 112.57 (Cq, C^10^), 107.31 (Cq, C^8a^), 43.94 (CH_2_, C^6^), 34.87 (CH_2_, C^5^). Solubility in water/1% DMSO ≥ 1.0 mg mL^–1^. Anal. Calcd for C_23_H_18_BrN_5_·0.8H_2_O (*M*_r_ 458.74), %: C, 60.22; H, 4.31; N, 15.27. Found, %: C, 60.41; H, 4.26; N, 15.48. IR spectrum (selected bands, ATR, ν_max_, cm^–1^): 1590.56 (w), 1541.24 (s), 1497.13 (m), 1474.21 (m), 1441.48 (m), 1399.55 (s), 1318.83 (m), 1285.99 (w), 1247.26 (w), 1148.64 (w), 1113.87 (w), 1026.79 (w), 997.62 (w), 920.68 (w), 865.22 (w), 801.84 (w), 768.74 (s), 751.79 (s), 684.75 (w), 662.45 (w), 625.94 (w). (+)ESI-MS (acetonitrile/methanol + 1% water): *m*/*z* 444.14; calcd *m*/*z* for [C_23_H_18_BrN_5_]^+^ or [M + H]^+^ 444.08.

**HL**^**5**^**·0.5H**_**2**_**O**: To a solution of **h**_**3**_ (160 mg, 0.45 mmol) in anoxic methanol (5 mL) in a 25 mL Schlenk tube was added 2-acetylpyridine (51 μL, 0.45 mmol), and the mixture was stirred at 75 °C overnight. On the next day, the reaction mixture was cooled to room temperature and concentrated under reduced pressure. The yellow product was precipitated by addition of diethyl ether (5 mL) and filtered off. Yield: 178 mg, 86%. ^1^H NMR (600 MHz, CDCl_3_): δ, ppm: 8.62 (d, *J* = 4.1 Hz, 1H, H^19^), 8.46 (d, *J* = 1.8 Hz, 1H, H^9^), 8.33 (s, 1H), 8.10 (d, *J* = 8.0 Hz, 1H, H^22^), 7.68–7.64 (m, 1H, H^21^), 7.45–7.38 (m, 2H, H^2,3^), 7.39–7.34 (m, 2H, H^1,4,11^), 7.29 (d, *J* = 8.5 Hz, 1H, H^12^), 7.23 (dd, *J* = 6.3, 4.9 Hz, 1H, H^20^), 3.69–3.61 (m, 2H, H^6^), 3.10 (t, *J* = 7.0 Hz, 2H, H^5^), 2.62 (s, 3H, 3H^23^). ^13^C{H} NMR (176 MHz, CDCl_3_): δ, ppm: 160.79 (Cq, C^16^), 158.84 (Cq, C^8^), 157.31 (Cq, C^17^), 148.87 (CH, C^19^), 139.12 (Cq, C^13a^), 138.59 (Cq, C^4a^), 135.89 (CH, C^21^), 135.07 (Cq, C^12a^), 132.54 (Cq, C^13b^), 129.92 (CH, C^4^), 129.66 (CH, C^3^), 129.38 (Cq, C^8b^), 129.16 (CH, C^2^), 126.96 (CH, C^1^), 126.12 (CH, C^11^), 125.38 (CH, C^9^), 123.45 (CH, C^20^), 120.93 (CH, C^22^), 114.75 (Cq, C^10^), 112.27 (CH, C^12^), 109.91 (Cq, C^8a^), 45.96 (CH_2_, C^6^), 34.59 (CH_2_, C^5^), 14.00 (CH_3_, C^23^). Solubility in water/1% DMSO ≥ 1.0 mg mL^–1^. Anal. Calcd for C_24_H_20_BrN_5_·0.5H_2_O (*M*_r_ 467.36), %: C, 61.68; H, 4.53; N, 14.98. Found, %: C, 61.47; H, 4.27; N, 14.79. IR spectrum (selected bands, ATR, ν_max_, cm^–1^): 3055.33 (w), 1588.28 (m), 1542.01 (s), 1499.47 (w), 1462.83 (s), 1384.67 (m), 1361.24 (m), 1299.43 (m), 1250.58 (w), 1150.86 (w), 1103.03 (w), 1040.08 (s), 992.90 (w), 964.58 (w), 921.14 (w), 866.65 (w), 779.44 (s), 749.48 (s), 676.25 (s), 644.33 (w). ESI-MS (acetonitrile/methanol + 1% water): *m*/*z* 460.15; calcd *m*/*z* for [C_24_H_20_BrN_5_]^+^ or [M + H]^+^ 460.10.

### Synthesis of Complexes **1a** and **1b**

**1a·1.5H**_**2**_**O**: To a solution of **HL**^**1**^ (80 mg, 0.22 mmol) in 2-propanol (32 mL) at 60 °C [Ru(*p*-cymene)Cl_2_]_2_ (67 mg, 0.11 mmol), chloroform (1 mL) was added, and the mixture was stirred at 50 °C for 1 h, cooled to room temperature, and then placed in the fridge overnight. On the next day, the product was filtered off as an orange powder. Yield: 110 mg, 68%. ^1^H NMR (700 MHz, DMSO): δ 12.17 (s, 1H, H^13^), 9.56 (d, *J* = 4.5 Hz, 1H, H^18^), 8.99 (s, 1H, H^15^), 8.29 (m, 2H, H^4,20^), 8.26 (s, 1H, H^9^), 7.86 (m, 1H, H^19^), 7.70 (d, *J* = 7.6 Hz, 1H, H^11^), 7.63 (d, *J* = 8.4 Hz, 1H, H^12^), 7.53–7.42 (m, 5H, H^1,2,3,7,21^), 6.17 (d, *J* = 5.7 Hz, 1H, H^23^), 5.75 (d, *J* = 5.5 Hz, 1H, H^22^), 5.61 (dd, *J* = 22.8, 5.9 Hz, 2H, H^22,23^), 3.62 (dd, *J* = 13.4, 4.7 Hz, 1H, H^6^), 3.44 (d, *J* = 5.7 Hz, 1H, H^6^), 3.15 (dd, *J* = 16.7, 10.1 Hz, 1H, H^5^), 3.10–3.03 (m, 1H, H^5^), 2.25 (s, 3H, H^28^) 2.08 (s, 1H, H^25^), 0.99 (d, *J* = 6.8 Hz, 3H, H^26^), 0.96 (d, *J* = 6.8 Hz, 3H, H^26^). ^13^C NMR (176 MHz, DMSO): δ 168.59 (Cq, C^8^), 165.14 (CH, C^15^), 155.92 (CH, C^18^), 154.97 (Cq, C^16^), 145.12 (Cq, C^10^), 139.87 (CH, C^20^), 139.16 (Cq, C^4a^), 137.91 (Cq, C^13b^), 136.79 (Cq, C^12a^), 131.24 (Cq, C^13a^), 130.56 (CH, C^3^), 130.25 (CH, C^1^), 129.55 (CH, C^21^), 129.24 (CH, C^4^), 128.43 (CH, C^19^), 127.43 (Cq, C^8b^), 126.46 (CH, C^2^), 117.13 (CH, C^11^), 115.58 (CH, C^9^), 112.10 (CH, C^12^), 110.99 (Cq, C^8a^), 104.88 (Cq, C^27^), 104.06 (Cq, C^24^), 87.68 (CH, C^23^), 86.37 (CH, C^23^), 84.74 (CH, C^22^), 84.18 (CH, C^22^), 43.97 (CH_2_, C^6^), 34.08 (CH_2_, C^5^), 30.49 (CH, C^25^), 22.13 (CH_3_, C^26^), 21.30 (CH_3_, C^26^), 18.53 (CH_3_, C^28^). Anal. Calcd for C_33_H_32_Cl_2_N_4_ORu·1.5H_2_O (*M*_r_ 699.63), %: C, 56.65; H, 5.04; N, 8.01. Found, %: C, 56.35; H, 4.77; N, 7.77. IR spectrum (selected bands, ATR, ν_max_, cm^–1^): 3063.74 (w), 1621.64 (s), 1539.20 (w), 1471.26 (s), 1446.98 (s), 1371.19 (w), 1326.22 (w), 1232.75 (w), 1162.09 (w), 1108.94 (w), 1031.43 (w), 935.72 (w), 877.37 (m), 818.90 (m), 766.60 (m), 682.28 (w). (+)ESI-MS (acetonitrile/methanol + 1% water): *m*/*z* 637.19; calcd *m*/*z* for [C_33_H_31_ClN_4_Ru]^+^ or [Ru^II^Cl(**HL**^**1**^)]^+^ 637.14.

**1b·1.2H**_**2**_**O**: To a solution of **HL**^**1**^ (50 mg, 0.14 mmol) in methanol (2 mL) at 60 °C, a solution of [Ru(*p*-cymene)Cl_2_]_2_ (48 mg, 0.07 mmol) in chloroform (0.6 mL) was added. The resulting mixture was stirred at 50 °C for 1 h, cooled to room temperature, and then placed in the fridge overnight. On the next day, the product was filtered off and isolated as a red powder. Yield: 76 mg, 72%. ^1^H NMR (600 MHz, DMSO-*d*_6_): δ, ppm: 12.21 (s, 1H, H^13^), 9.51 (d, *J* = 5.6 Hz, 1H, H^18^), 9.39 (s, 1H, H^15^), 8.41 (d, *J* = 7.2 Hz, 1H, H^4^), 8.27 (td, *J* = 7.7, 1.3 Hz, 1H, H^20^), 8.18 (s, 1H, H^9^), 7.82 (ddd, *J* = 7.5, 5.7, 1.5 Hz, 1H, H^19^), 7.62 (d, *J* = 1.3 Hz, 2H; H^11;12^), 7.52–7.41 (m, 5H, H^1,2,3,7,21^), 6.47 (d, *J* = 5.8 Hz, 1H, 1H, H^23^), 5.97 (d, *J* = 5.8 Hz, 1H, 1H, H^22^), 5.83 (d, *J* = 5.7 Hz, 1H, 1H, H^23^), 5.78 (d, *J* = 5.7 Hz, 1H, 1H, H^22^), 3.61 (ddd, *J* = 13.6, 10.5, 6.2 Hz, 1H, H^6^), 3.43 (ddt, *J* = 10.9, 8.5, 5.4 Hz, 1H, H^6^), 3.15 (ddd, *J* = 14.7, 8.6, 6.0 Hz, 1H, H^5^), 3.06 (dt, *J* = 15.2, 6.4 Hz, 1H, H^5^), 2.38 (dd, *J* = 5.7, 4.3 Hz, 1H, H^25^), 2.32 (s, 3H, H^28^), 0.92 (d, *J* = 6.9 Hz, 3H, H^26^), 0.88 (d, *J* = 6.9 Hz, 3H, H^26^). ^13^C{H} NMR (176 MHz, DMSO-*d*_6_): δ, ppm: 168.56 (Cq, C^8^), 165.97 (CH, C^15^), 156.40 (Cq, C^16^), 155.54 (CH, C^18^), 145.09 (Cq, C^10^), 139.96 (CH, C^20^), 139.20 (Cq, C^4a^), 137.92 (Cq, C^13b^), 136.88 (Cq, C^12a^), 131.22 (Cq, C^13a^), 130.53 (CH, C^3^), 130.23 (CH, C^1^), 129.35 (CH, C^21^), 129.24 (CH, C^19^), 129.19 (CH, C^4^), 127.41 (Cq, C^8b^), 126.45 (CH, C^2^), 117.32 (CH, C^11^), 116.22 (Cq, C^9^), 112.14 (CH, C^12^), 110.97 (Cq, C^8a^), 98.91 (Cq, C^27^), 95.75 (Cq, C^24^), 79.77 (CH, C^23^), 78.17 (CH, C^23^), 75.28 (CH, C^22^), 74.26 (CH, C^22^), 43.99 (CH_2_, C^6^), 34.02 (CH_2_, C^5^), 30.76 (CH, C^25^), 22.29 (CH_3_, C^26^), 21.62 (CH_3_, C^26^), 18.45 (CH_3_, C^28^). Anal. Calcd for C_33_H_32_Cl_2_N_4_OOs·1.2H_2_O (*M*_r_ 783.39), %: C, 50.59; H, 4.43, N, 7.15. Found, %: C, 50.30; H, 4.36; N, 7.05. Solubility in water/1% DMSO ≥ 1.0 mg mL^–1^. IR spectrum (selected bands, ATR, ν_max_, cm^–1^): 3060.66 (w), 2967.48 (w), 1616.81 (s), 1540.10 (w), 1445.34 (s), 1371.34 (w), 1325.17 (w), 1231.54 (w), 1160.42 (w), 1108.91 (w), 1030.87 (w), 935.03 (w), 879.05 (m), 819.46 (w), 743.39 (w), 664.63 (w). (+)ESI-MS (acetonitrile/methanol + 1% water): *m*/*z* 727.21; calcd *m*/*z* for [C_33_H_31_ClN_4_Os]^+^ or [Os^II^Cl(**HL**^**1**^)]^+^ 727.19.

### Synthesis of Complexes **2–5**

**2**: To a solution of **HL**^**2**^ (90 mg, 0.25 mmol) in 2-propanol (40 mL), a solution of CuCl_2_·2H_2_O (42 mg, 0.25 mmol) in methanol (1 mL) was added. The reaction mixture was heated to reflux for 15 min, cooled down, and allowed to stand at 4 °C overnight. The product was filtered off and dried in vacuo to give a green powder. Yield: 66.5 mg, 77%. Anal. Calcd for C_23_H_19_Cl_2_CuN_5_ (*M*_r_ 498.03), %: C, 55.41; H, 3.84, N, 14.05. Found, %: C, 55.09; H, 3.54; N, 13.65. Solubility in water/1% DMSO ≥ 1.0 mg mL^–1^. IR spectrum (selected bands, ATR, ν_max_, cm^–1^): 3060.18 (w), 1607.79 (w), 1564.78 (m), 1501.60 (m), 1435.66 (m), 1316.81 (w), 1238.42 (w), 1162.49 (w), 968.14 (w), 846.95 (m), 744.14 (s), 697.44 (s), 650.05 (w). (+)ESI-MS (acetonitrile/methanol + 1% water): 463.11; calcd *m*/*z* for [C_23_H_19_ClCuN_5_]^+^ or [Cu^II^Cl(**HL**^**2**^)]^+^ 463.06.

**3·CH**_**3**_**OH·H**_**2**_**O**: To a solution of **HL**^**3**^ (90 mg, 0.24 mmol) in ethanol (12 mL), a solution of CuCl_2_·2H_2_O (41 mg, 0.24 mmol) in methanol (1 mL) was added. The reaction mixture was heated to reflux for 15 min, cooled down, and allowed to stand at room temperature overnight. The product was filtered off and dried in vacuo to give a green powder. Yield: 91 mg, 81%. Anal. Calcd for C_24_H_21_Cl_2_CuN_5_·CH_3_OH·H_2_O (*M*_r_ 563.97), %: C, 53.24; H, 4.83, N, 12.42. Found, %: C, 53.18; H, 4.33; N, 12.53. Solubility in water/1% DMSO ≥ 1.0 mg mL^–1^. IR spectrum (selected bands, ATR, ν_max_, cm^–1^): 3622.27 (w), 3064.74 (w), 2875.50 (w), 1603.86 (s), 1545.84 (w), 1503.81 (s), 1432.83 (s), 1381.62 (w), 1332.03 (w), 1289.57 (w), 1268.10 (w), 1237.76 (w), 1181.20 (s), 1157.40 (s), 1107.75 (s), 1045.34 (w), 1019.23 (w), 956.02 (w), 834.84 (w), 751.76 (s), 723.62 (s), 647.66 (s). (+)ESI-MS (acetonitrile/methanol + 1% water): *m*/*z* 477.12; calcd *m*/*z* for [C_24_H_21_ClCuN_5_]^+^ or [Cu^II^Cl(**HL**^**3**^)]^+^ 477.08. X-ray diffraction quality single crystals were obtained by slow evaporation of a methanolic solution of **3**.

**4·CH**_**3**_**OH**: To a solution of **HL**^**4**^ (20 mg, 0.05 mmol) in 2-propanol (6 mL), a solution of CuCl_2_·2H_2_O (8 mg, 0.05 mmol) in methanol (1 mL) was added. The reaction mixture was heated to reflux for 15 min, cooled down, and allowed to stand at room temperature overnight. The product was filtered off and dried in vacuo to give a green powder. Yield: 20 mg, 78%. Anal. Calcd for C_23_H_18_BrCl_2_CuN_5_·CH_3_OH (*M*_r_ 607.97), %: C, 47.37; H, 3.65; N, 11.52. Found, %: C, 47.58; H, 3.20; N, 11.34. Solubility in water/1% DMSO ≥ 1.0 mg mL^–1^. IR spectrum (selected bands, ATR, ν_max_, cm^–1^): 3062.49 (w), 1606.34 (m), 1568.41 (w), 1502.78 (m), 1463.46 (s), 1433.39 (s), 1302.00 (m), 1220.88 (m), 1159.34 (m), 1099.86 (w), 1051.64 (w), 1022.70 (w), 959.19 (w), 922.26 (w), 872.23 (w), 773.13 (s), 696.07 (m), 682.11 (m), 652.26 (m). (+)ESI-MS (acetonitrile/methanol + 1% water): *m*/*z* 543.05; calcd *m*/*z* for [C_23_H_18_BrClCuN_5_]^+^ or [Cu^II^Cl(**HL**^**4**^)]^+^ 542.98.

**5·H**_**2**_**O**: To a solution of **HL**^**5**^ (41 mg, 0.09 mmol) in methanol (5 mL), a solution of CuCl_2_·2H_2_O (15 mg, 0.09 mmol) in methanol (1 mL) was added. The reaction mixture was heated to reflux for 15 min, cooled down, and allowed to stand at room temperature for 2 days. The product was filtered off and dried in vacuo to give a green powder. Yield: 28 mg, 70%. Anal. Calcd for C_24_H_20_BrCl_2_CuN_5_·H_2_O (*M*_r_ 607.97): C, 47.37; H, 3.65; N, 11.52. Found, %: C, 47.08; H, 3.86; N, 11.20. Solubility in water/1% DMSO ≥ 1.0 mg mL^–1^. IR spectrum (selected bands, ATR, ν_max_, cm^–1^): 3148.11 (w), 2926.56 (w), 1603.60 (w), 1538.31 (w), 1497.60 (w), 1461.29 (s), 1427.77 (s), 1371.99 (w), 1297.84 (m), 1266.14 (w), 1185.50 (s), 1157.80 (m), 1104.68 (s), 1051.44 (w), 1020.17 (w), 955.51 (w) 922.62 (w), 837.61 (w), 808.32 (w), 772.84 (m), 748.30 (w), 722.09 (m), 647.07 (w). (+)ESI-MS (acetonitrile/methanol + 1% water): *m*/*z* 557.04; calcd *m*/*z* for [C_24_H_20_BrClCuN_5_]^+^ or [Cu^II^Cl(**HL**^**5**^)]^+^ 556.99.

### Crystallographic Structure Determination

The measurements were performed on a Bruker X8 APEXII CCD and Bruker D8 Venture diffractometers. Single crystals were positioned at 27, 60, and 60 mm from the detector, and 500, 1000, and 9214 frames were measured, each for 8, 1, and 1 s over −0.360, −0.360, and 0.360° scan width for **f**_**2**_**·CH**_**2**_**Cl**_**2**_, **g**_**2**_**·CH**_**2**_**Cl**_**2**_, and **3·3MeOH**, respectively. The data were processed using SAINT software.^60^ Crystal data, data collection parameters, and structure refinement details are given in Table S1. The structures were solved by direct methods and refined by full-matrix least-squares techniques. Non-H atoms were refined with anisotropic displacement parameters. H atoms were inserted in calculated positions and refined with a riding model. The following computer programs and hardware were used: structure solution, SHELXS and refinement, SHELXL;^61^ molecular diagrams, ORTEP;^62^ computer, Intel CoreDuo. CCDC 2194805 (**f**_**2**_·CH_2_Cl_2_), 2194806 (**g**_**2**_·CH_2_Cl_2_) and 2194807 (**3**·3MeOH).

### Additional Determination of Purity of Ligand **HL**^**5**^ and Complex **5**

Reverse-phase (RP) HPLC analysis of compounds **HL**^**5**^ and **5** was performed on a system composed of a maXis UHR ESI-Qq-TOF mass spectrometer (Bruker Daltonics, Bremen, Germany) coupled to an HPLC system (UltiMate 3000, Dionex). Separation was carried out on a C18 analytical column AcclaimTM 120 (Thermo Scientific, 2.1 × 150 mm, 3 μm, 120 Å) at a flow rate of 0.3 mL/min. Column temperature: 25 °C. Mobile phase A: (100% MeOH + 0.1% FA). Mobile phase B: (100% ACN + 0.1% formic acid (FA)). UV: 254 nm, 280 nm, and 350 nm. The sum formulae of the detected ions were determined using Bruker Compass DataAnalysis 5.1 based on the mass accuracy (Δ*m*/z ≤ 5 ppm) and isotopic pattern matching (SmartFormula algorithm). A peak for [**HL**^**5**^ + H]^+^ (*m*/*z* = 458.0984) (Figure S27) and a peak for [Cu^II^(**HL**^**5**^)]^+^ (*m*/*z* = 521.0084) (Figure S28) were observed.

## Biological Studies

### Cell Cultures

Human tumor cell lines derived from human colorectal carcinoma, LS-174 and HCT116, human breast adenocarcinoma, MDA-MB-361, human lung adenocarcinoma, A549, and non-tumor human fetal lung fibroblast cell line, MRC-5, were maintained as a monolayer culture in the Roswell Park Memorial Institute (RPMI) 1640 nutrient medium (Sigma Chemicals Co, USA). RPMI 1640 medium was prepared in sterile ionized water, supplemented with penicillin (100 IU/mL), streptomycin (200 μg/mL), 4-(2-hydroxyethyl) piperazine-1-ethanesulfonic acid (HEPES) (25 mM), l-glutamine (3 mM), and 10% of heat-inactivated fetal calf serum (FCS) (pH = 7.2). The cells were grown at 37 °C in a humidified atmosphere containing 5% CO_2_.

### MTT Assay

Cells were seeded into 96-well culture plates (Thermo Scientific Nunc) at cell densities of 5000 cells per well (HCT116, A549, and MRC-5) and 8000 cells/well (LS-174 and MDA-MB-361) in 100 μL of cell culture medium and left overnight. Eleven tested compounds were dissolved in DMSO to the stock concentration of 20 mM immediately before the experiment, whereas further dilutions were made in the culture medium so that the final concentration of DMSO never exceeded 1% (v/v). Cisplatin (*cis*-diamminedichloridoplatinum(II), CDDP) was used as a reference compound. After 72 h of continuous incubation, MTT solution (3-(4.5-dimethylthiazol-2-yl)-2.5-diphenyltetrazolium bromide, Sigma-Aldrich) was added to each well (5 mg/mL).^63^ The culture plates were incubated at 37 °C for the next 4 h, and finally, 10% sodium dodecyl sulfate (SDS) was added to dissolve formed formazan crystals. Absorbances were measured after 24 h on a microplate reader (Thermo Labsystems Multiscan EX 200–240 V) at a wavelength of 570 nm. The IC_50_ values (concentration of the investigated compound that causes 50% decrease in the number of viable cells in a treated cell population compared to a non-treated control) were determined from the cell survival diagrams.

### Flow-Cytometric Analysis of Cell Cycle Phase Distribution

Quantitative analysis of cell cycle phase distribution was performed by flow-cytometric analysis in fixed cells after staining with propidium iodide (PI).^64^ Cells were seeded at a density of 2 × 10^5^ (HCT116) or 3 × 10^5^ (LS-174) cells/well into six-well plates (Thermo Scientific Nunc) with the cell culture medium and left overnight. The next day, media were changed with the desired media dilutions of investigated compounds or CDDP. After 24 h or 48 h of continuous treatment, cells were collected by trypsinization, washed twice with ice-cold phosphate-buffered saline (PBS), and fixed overnight in 70% ice-cold ethanol. After fixation, cells were washed again with PBS and incubated with RNaseA (1 mg/mL) for 30 min at 37 °C. Immediately before flow-cytometric analysis, cells were stained in the dark with PI (400 μg/mL in PBS). Cell cycle phase distribution was analyzed using a fluorescence-activated cell sorting (FACS) BD Calibur flow cytometer (Becton–Dickinson, Heidelberg Germany) and Cell Quest computer software.

### Annexin V-FITC Apoptotic Assay

Quantitative analysis of apoptotic and necrotic cell death induced by the investigated complexes and CDDP was performed by Annexin V-FITC/PI assay. 2 × 10^5^ HCT116 or 3 × 10^5^ LS-174 cells were seeded into six-well plates (Thermo Scientific Nunc), and after 24 h of growth, cells were treated with tested compounds or CDDP. Following the 24 h and 48 h incubation time, cells were harvested and resuspended in 200 μL 1 × Binding Buffer (10 mM HEPES/NaOH pH 7.4, 140 mM NaCl, 2.5 mM CaCl_2_). 100 μL of the cell suspension (∼1 × 10^5^ cells) was transferred to a 5 mL round-bottom polystyrene tube (Falcon, Corning) and mixed with 5 μL of both FITC Annexin V (BD Pharmingen^39^) and PI (50 μg/mL in PBS).^65^ After 15 min of incubation at 37 °C in the dark, 400 μL of 1 × Binding Buffer was added to each tube. Samples were analyzed using an FACS BD Calibur flow cytometer (Becton–Dickinson, Heidelberg Germany) and Cell Quest computer software.

### Morphological Analysis

Cells were seeded into six-well plates (Thermo Scientific Nunc) as a flat monolayer culture. After 24 h of growth, cells were exposed to the tested compounds or CDDP. Following 24 h or 48 h of treatment, cells were visualized by bright-field microscopy (Carl Zeiss, Jena, Germany) at 6.3× magnification using a digital camera (Olympus, USA).

### Generation and Analysis of MCTSs

The MCTSs as 3D cell culture models were established by seeding cells at optimized densities between 500 and 1500 cells/well in the ultra-low attachment (ULA) U96-well plate Thermo Scientific Nunclon Sphera in their respective culture media.^34^ MCTS aggregates were formed after 4 days of incubation in 5% CO_2_ at 37 °C. The formation and growth of MCTSs were examined and imaged every 24 h using an inverted microscope (Carl Zeiss, Jena, Germany) (6.3× objective), equipped with a digital camera (Olympus, USA). The spheroids of appropriate dimensions (>500 μm in diameter) were treated by carefully adding a medium with freshly made serial dilutions of ligand **HL**^**5**^, complex **5** (up to 20 μM), and cisplatin (up to 80 μM) and incubation for another 72 h. The cytotoxicity was investigated by MTT assay. Spheroids were photographed after 72 h.

### Kinase Inhibition Assays

All kinase assays were carried out robotically^52^ in a total assay volume of 25.5 μL. To plates containing 0.5 μL of compounds, DMSO controls, or acid blanks, 15 μL of an enzyme mix containing the enzyme and peptide/protein substrate in the buffer was added. Assays were performed for 30 min using Multidrop Micro reagent dispensers (Thermo Electron Corporation, Waltham, MA, U.S.A.) in a 96-well format. The concentration of magnesium acetate in the assays was 10 mM, and [γ-^33^P]ATP (800 c.p.m./pmol) was used at 5, 20, or 50 μM in order to be at or below the *K*m for ATP for each enzyme. The assays were initiated with MgATP, stopped by the addition of 5 μL of 0.5 M orthophosphoric acid, spotted onto P81 filter plates using a unifilter harvester (PerkinElmer, Boston, MA, U.S.A.), and air-dried. The dry Unifilter plates were then sealed on the addition of MicroScint O and are counted in PerkinElmer Topcount scintillation counters. The IC_50_ values of inhibitors were determined after carrying out assays at 10 different concentrations of **HL**^**5**^ and **5** obtained by dilution of the 1 mM stock solution of each compound in DMSO. The substrates used for protein kinases (GSK-3α, GSK-3β, CDK2, CDK5, CDK9, Src, Lck) were reported previously.^52^ Unless stated otherwise, enzymes were diluted in a buffer consisting of 50 mM Tris/HCl, pH 7.5, 0.1 mM EGTA (ethylene glycol-bis(β-aminoethyl ether)-*N*,*N*,*N*′,*N*′-tetraacetic acid), 1 mg/mL BSA, and 0.1% 2-mercaptoethanol and assayed in a buffer comprising 50 mM Tris/HCl, pH 7.5, 0.1 mM EGTA, and 0.1% 2-mercaptoethanol.

## Supporting Information Available

Multistep synthesis of **HL**^**1**^–**HL**^**5**^**1a**, **1b**, and **2**–**5**; details of the synthesis of all intermediate species; additional X-ray crystallographic data; NMR spectra; ESI-MS data; HPLC MS data; overlay of the X-ray structure of **3** with that of a related complex based on 5,8-dihydroindolo[3,2-*d*]benzazepin-7(6*H*)-one; UV–vis data; time-dependent ^1^H NMR spectra; plot diagrams of Annexin V-FITC apoptosis assay; enzyme inhibition data; and NMR numbering scheme (PDF)
