# Supplementary material for: Enriching Chemical Space of Bioactive Scaffolds by New Ring Systems: Benzazocines and Their Metal Complexes as Potential Anticancer Drugs
Source: Inorg Chem. 2022 Dec 6;61(50):20445–60. doi: 10.1021/acs.inorgchem.2c03134 (PMC9768754; doi:10.1021/acs.inorgchem.2c03134)
Supplement: Supplementary file 1 — ic2c03134_si_001.pdf [file ic2c03134_si_001.pdf]

Supporting Information

for

Enriching chemical space of bioactive scaffolds by new ring systems: benzazocines and their metal complexes as potential anticancer drugs

Irina Kuznetcova,[†] Marija Ostojić,[#] Nevenka Gligorijević,[#] Sandra Aranđelović,[#,*] Vladimir B. Arion[†,*]

[†]*Institute of Inorganic Chemistry of the University of Vienna, Währinger Strasse 42, 1090 Vienna, Austria*

[#]*Department of Experimental Oncology, Institute for Oncology and Radiology of Serbia, Pasterova 14, 11000 Belgrade, Serbia*

Table of Contents

Synthesis of **HL**^{1}–**HL**^{5}**, 1a**, **1b** and **2–5** S3

X-ray crystallography data S11

NMR-spectra of **HL**^{1}–**HL**^{5} S12

ESI-MS spectra of **HL**^{2}–**HL**^{5}, **1a**, **1b**, **2–5** S23

NMR spectra of **1a** and **1b** S33

High Performance Liquid Chromatography-MS S38

Overlay of **3** with a related complex based on 5,8-dihydroindolo[3,2-*d*]benzazepin-7(6*H*)-one S40

UV-vis spectra S41

^{1}H NMR spectra of **HL**^{5} in DMSO-d_6/D_2O S42

Representative dot plot diagrams S43

Enzymes inhibition S44

NMR spectra of intermediate species S46

High resolution ESI mass spectrum of **HL**^{1} S66

NMR numbering schemes S67

Crystal data and details of data collection for $\mathbf{f_2}$, $\mathbf{g_2}$ and **3** S68

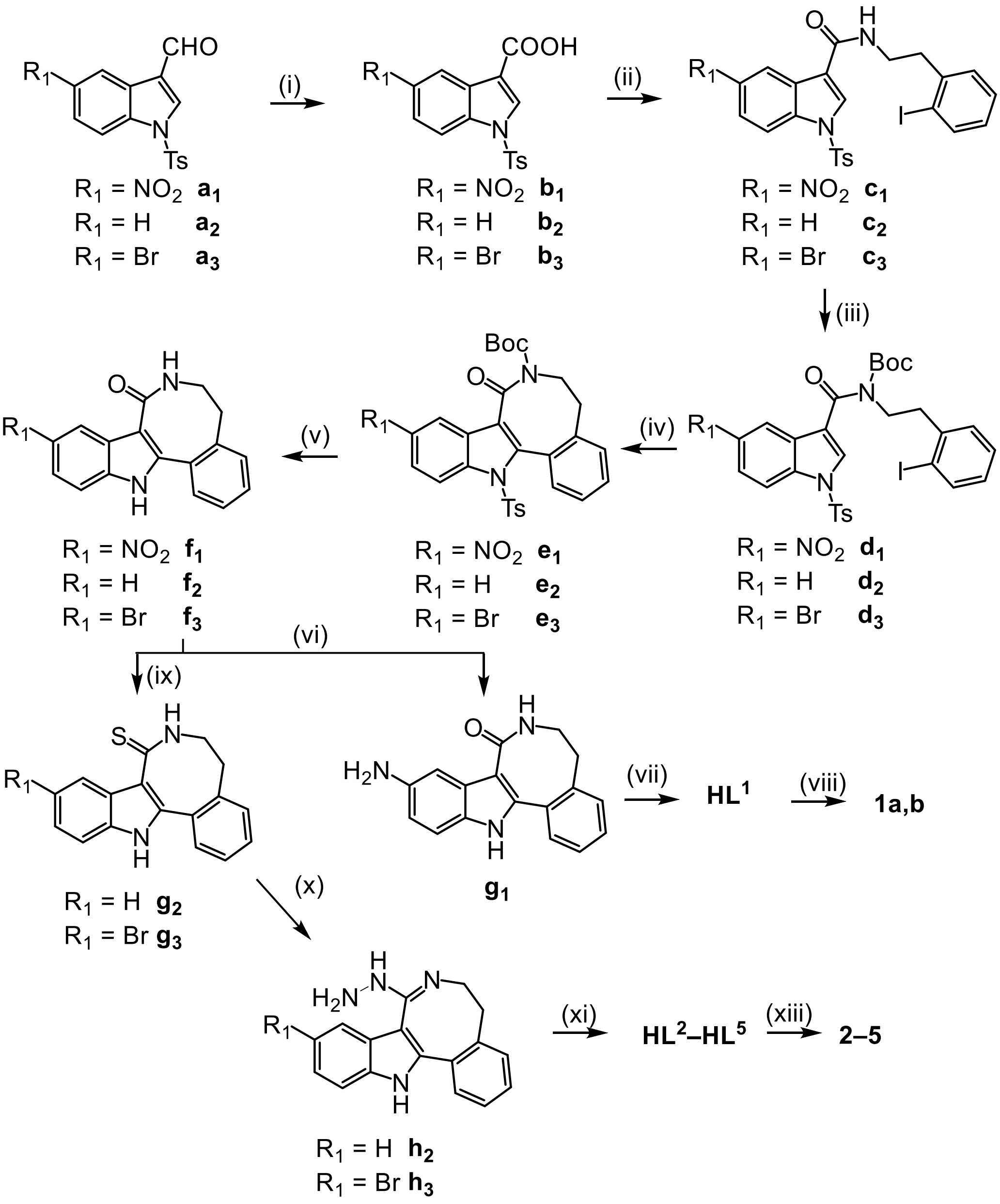

Scheme S1. Synthesis of indolo[3,2-*e*]benzazocines **HL¹–HL⁵**, ruthenium(II) and osmium(II) complexes **1a** and **1b**, as well as copper(II) complexes **2–5**. Reagents and conditions: (i) $NaClO_2$, H_3NSO_3, THF, rt; (ii) 2-iodobenzeneethylamine, DMAP, EDCI·HCl, CH_2Cl_2, 0 °C → rt, 24 h; (iii) Boc_2O, DMAP, MeCN (dry), rt, 12 h; (iv) PPh_3, $Pd(OAc)_2$, Ag_2CO_3, DMF (dry), 80–110 °C, 2 h; (v) CF_3COOH, CH_2Cl_2, rt, 2 h; TBAF, THF (dry), reflux, 1 h; (vi) Pd/C, THF (dry), 4 bar, 12 h; (vii) 2-formylpyridine, EtOH, 85 °C, 20 h; (viii) **1a**: [Ru(*p*-cymene)Cl_2]$_2$, *i*-PrOH, $CHCl_3$, 60 °C, 1 h; **1b**: [Os(*p*-cymene)Cl_2]$_2$, MeOH, $CHCl_3$, 50 °C, 1 h; (ix) P_4S_{10}, Al_2O_3, THF (dry), 75 °C, overnight; (x) $N_2H_4 \cdot H_2O$, $CHCl_3$, rt, 3 h; (xi) **HL²**: 2-formylpyridine, EtOH, 75 °C, overnight; **HL³**:

2-acetylpyridine, EtOH, 75 °C, overnight; **HL^4**: 2-formylpyridine, MeOH, 75 °C, overnight; **HL^5**: 2-acetylpyridine, MeOH, 75 °C, overnight. (xii) **2**: $CuCl_2·H_2O$, 2-propanol, reflux, 15 min; **3**: $CuCl_2·H_2O$, ethanol, reflux, 15 min; **4**: $CuCl_2·H_2O$, *i*-propanol, reflux, 15 min; **5**: $CuCl_2·H_2O$, methanol, reflux, 15 min.

Synthesis of the main organic scaffolds b_1–h_3

Indole-3-carboxylic acid **b_2**[47] was prepared by following a known protocol. Species **b_1**, **b_3** were synthesized analogously starting from 5-nitro-1-tosyl-indole-3-carboxaldehyde[48] **a_1** and 5-bromo-1-tosyl-indole-3-carboxaldehyde[49] **a_3** as described below.

Species b_1. To a suspension of 5-nitro-1-tosyl-1*H*-indole-3-carboxaldehyde[48] **a_1** (8.26 g, 23.92 mmol) in THF (150 mL) a solution of sodium chlorite (2.77 g, 30.62 mmol) and sulfamic acid (12.98 g, 133.70 mmol) in water (150 mL) were added. The resulting mixture was stirred at room temperature for 1 h. The reaction was monitored by TLC using DCM as eluent. After its completeness a saturated solution of $NaHCO_3$ was added to reach pH = 8. The product was extracted with EtOAc (3 × 150 mL). The organic phases were combined, and the solvent was evaporated under reduced pressure on a rotary evaporator. The crude product was crystallized from MeOH. Yield: 7.9 g, 94%. ^{1}H NMR (500 MHz, DMSO-d_6) δ, ppm: 13.32 (br s, 1H), 8.85 (d, J = 2.2 Hz, 1H), 8.61 (s, 1H), 8.27 (dd, J = 9.2, 2.3 Hz, 1H), 8.21 (d, J = 9.2 Hz, 1H), 8.11 (d, J = 8.5 Hz, 2H), 7.47 (d, J = 8.2 Hz, 2H), 2.35 (s, 3H). ESI-MS (acetonitrile/methanol + 1% water), positive: m/z 383.03 $[M + Na]^+$.

Species b_3. To a suspension of 5-bromo-1-tosyl-1*H*-indole-3-carboxaldehyde[49] **a_3** (8.86 g, 23.02 mmol) in THF (140 mL) a solution of sodium chlorite (4.21 g, 46.60 mmol) and sulfamic acid (12.14 g, 125.03 mmol) in water (150 mL) were added. The resulting mixture was stirred for 1 h. The reaction end was established by TLC using DCM as eluent. Then a saturated solution of $NaHCO_3$ was added to reach pH = 8. The product was extracted with EtOAc (3 × 150 mL). The organic phases were combined, the solvent was removed on a rotary evaporator and the residue crystallizied from MeOH. Yield: 8.7 g, 96%. ^{1}H NMR (500 MHz, DMSO-d_6) δ, ppm: 13.18 (br s, 1H), 8.40 (s, 1H), 8.16 (d, J = 1.9 Hz, 1H), 8.03 (d, J = 8.5 Hz, 2H), 7.94 (d, J = 8.9 Hz, 1H), 2.35 (s, 3H). ESI-MS (acetonitrile/methanol + 1% water), negative: m/z 391.84 $[M – H]^–$.

Species c$_1$. Under argon atmosphere a solution of the freshly prepared 2-iodobenzeneethylamine (2.90 g, 11.74 mmol) in DCM (100 mL) was cooled to 0 °C and 5-nitro-1-tosyl-1*H*-indole-3-carboxylic acid (**b$_1$**) (3.74 g, 10.84 mmol) was added, followed by EDCI·HCl (2.25 g, 11.74 mmol) and DMAP (1.32 g, 10.84 mmol). The reaction mixture was stirred in an ice bath for 4 h and then at room temperature for 20 h. Thereafter water (30 mL) was added, the mixture was acidified with 6 M HCl to pH = 1 and extracted with DCM (3 × 150 mL). The combined organic phases were dried over magnesium sulfate and concentrated on a rotary evaporator. The product was washed with ice-cold diethyl ether. Yield: 5.80 g, 91%. ^{1}H NMR (500 MHz, DMSO-d_6) δ, ppm: 9.11 (t, J = 5.5 Hz, 1H), 9.02 (d, J = 2.1 Hz, 1H), 8.91 (s, 1H), 8.25 (dd, J = 9.2, 2.2 Hz, 1H), 8.17 (d, J = 9.2 Hz, 1H), 8.00 (d, J = 8.3 Hz, 2H), 7.90 (d, J = 7.8 Hz, 1H), 7.48 (d, J = 8.2 Hz, 2H), 7.41 (d, J = 4.3 Hz, 2H), 7.10 – 7.02 (m, 1H), 4.47 (d, J = 5.4 Hz, 2H), 2.36 (s, 3H).

Species c2. Under argon atmosphere to a freshly prepared solution of 2-iodobenzeneethylamine (2.00 g, 8.10 mmol) in DCM (80 mL) cooled to 0 °C 1-tosyl-1*H*-indole-3-carboxylic acid (**b$_2$**) (2.36 g, 7.48 mmol) was added, followed by EDCI·HCl (1.60 g, 8.10 mmol) and DMAP (0.91 g, 7.48 mmol). This mixture was stirred in an ice bath for 4 h and then at room temperature for 20 h. Thereafter water (30 mL) was added, the mixture was acidified with 6 M HCl to pH = 1 and extracted with DCM (3 × 60 mL). The combined organic phases were dried over magnesium sulfate and the solvent was removed on a rotary evaporator. The product was washed with ice-cold diethyl ether. Yield: 3.77 g, 98%. ^{1}H NMR (500 MHz, DMSO) δ, ppm: 8.57 (t, J = 5.5 Hz, 1H), 8.50 (s, 1H), 8.14 (d, J = 7.6 Hz, 1H), 7.94 – 7.88 (m, 3H), 7.86 (d, J = 7.5 Hz, 1H), 7.43 (d, J = 8.1 Hz, 2H), 7.40 – 7.28 (m, 4H), 6.99 (m, 1H), 3.47 (m, 2H), 2.99 – 2.93 (m, 2H), 2.33 (s, 3H).

Species c$_3$. Under argon atmosphere to a solution of freshly prepared 2-iodobenzeneethylamine (3.00 g, 12.15 mmol) in DCM (120 mL) cooled to 0 °C 5-bromo-1-tosyl-1*H*-indole-3-carboxylic acid (**b$_3$**) (4.40 g, 11.20 mmol) was added, followed by EDCI·HCl (2.33 g, 12.15 mmol) and DMAP (1.37 g, 1.20 mmol). This solution was stirred in an ice bath for 4 h and then at room temperature for 20 h. Thereafter water (30 mL) was added, the mixture was acidified with 6 M HCl to pH = 1 and extracted with DCM (3 × 60 mL). The combined organic phases were dried over magnesium sulfate and the solvent was evaporated on a rotary evaporator. The

product was washed with ice-cold diethyl ether. Yield: 6.15 g, 88%. ^{1}H NMR (500 MHz, DMSO-d_6) δ, ppm: 8.62 (t, *J* = 5.5 Hz, 1H), 8.57 (s, 1H), 8.29 (d, *J* = 1.9 Hz, 1H), 7.92 – 7.84 (m, 4H), 7.53 (dd, *J* = 8.9, 2.1 Hz, 1H), 7.45 (d, *J* = 8.1 Hz, 2H), 7.37 – 7.30 (m, 2H), 6.99 (m, 1H), 3.47 (dd, *J* = 14.0, 6.3 Hz, 2H), 2.96 (t, *J* = 7.4 Hz, 2H), 2.34 (s, 3H).

Species d1. Under argon atmosphere a mixture of **c1** (5.80 g, 9.84 mmol), DMAP (0.12 g, 0.98 mmol) and Boc$_2$O (3.43 g, 15.74 mmol) in absolute THF (180 mL) was stirred at room temperature overnight. Then the solvent was removed on a rotary evaporator. The crude solid was washed with water and extracted with ethyl acetate (3 $\times$ 150 mL). The organic phases were combined, dried over magnesium sulfate and the solvent was removed on a rotary evaporator. The residue was purified on a silica column by using a mixture of hexane:ethyl acetate 3:1 as eluent. Yield: 6.40 g, 95%. ^{1}H NMR (500 MHz, DMSO-d_6) δ, ppm: 8.72 (s, 1H), 8.58 (s, 1H), 8.29 (s, 2H), 8.10 (d, *J* = 8.5 Hz, 2H), 7.91 (d, *J* = 6.9 Hz, 1H), 7.48 (d, *J* = 8.3 Hz, 2H), 7.43 (t, *J* = 7.1 Hz, 1H), 7.19 (d, *J* = 7.7 Hz, 1H), 7.06 (t, *J* = 7.6 Hz, 1H), 4.87 (s, 2H), 2.36 (s, 3H), 0.97 (s, 9H).

Species d2. Under argon atmosphere a mixture of **c$_2$** (11.40 g, 22.27 mmol), DMAP (0.27 g, 2.22 mmol) and Boc$_2$O (7.77 g, 35.63 mmol) in absolute acetonitrile (240 mL) was stirred at room temperature overnight. Then the solvent was removed on a rotary evaporator. The crude solid was washed with water and extracted with ethyl acetate (3 $\times$ 200 mL). The organic phases were combined and dried over magnesium sulfate. The solvent was removed under reduced pressure. The residue was purified on a silica column by using a mixture of hexane:ethyl acetate 3:1 as eluent. Yield: 13.48 g, 94%. ^{1}H NMR (500 MHz, DMSO) δ, ppm 8.11 (s), 7.99 (d, *J* = 8.4 Hz), 7.83 (d, *J* = 7.6 Hz), 7.54 (d, *J* = 7.9 Hz), 7.42 (dd, *J* = 16.7, 7.9 Hz), 7.37 – 7.28 (m), 7.00 – 6.95 (m), 3.99 (t, *J* = 7.1 Hz), 3.10 (t, *J* = 7.1 Hz), 2.34 (s), 0.92 (s). ESI-MS (acetonitrile/methanol + 1% water), positive: *m/z* 667.16 $[M + Na]^{+}$.

Species d3. Under argon atmosphere a mixture of **c3** (7.45 g, 11.96 mmol), DMAP (0.15 g, 1.20 mmol) and Boc$_2$O (4.17 g, 19.14 mmol) in absolute acetonitrile (200 mL) was stirred at room temperature overnight. The solvent was removed under reduced pressure. The crude solid was washed with water and extracted with ethyl acetate (3 $\times$ 200 mL). The organic phases were combined and dried over magnesium sulfate. The solvent was removed on a rotary evaporator. The residue was purified on a silica column by using a mixture of hexane:ethyl acetate 3:1 as eluent. Yield: 7.22 g, 83%.

^{1}H NMR (500 MHz, DMSO-d_6) δ, ppm: 8.23 (s, 1H), 8.01 (d, J = 8.4 Hz, 2H), 7.96 (d, J = 8.9 Hz, 1H), 7.85 – 7.81 (m, 1H), 7.59 (m, 1H), 7.56 (dd, J = 8.8, 2.0 Hz, 1H), 7.45 (d, J = 8.2 Hz, 2H), 7.35 – 7.27 (m, 2H), 7.00 – 6.95 (m, 1H), 4.01 (t, J = 6.9 Hz, 2H), 3.09 (t, J = 7.0 Hz, 2H), 2.35 (s, 3H), 0.94 (s, 9H). ESI-MS (acetonitrile/methanol + 1% water), positive: m/z 745.06 $[M + Na]^+$.

Species e$_1$. Under argon atmosphere a mixture of **d$_1$** (6.40 g, 9.28 mmol), triphenylphosphine (1.22 g, 4.64 mmol), palladium(II) acetate (0.52 g, 2.32 mmol) and silver(I) carbonate (6.40 g, 23.20 mmol) in dry DMF (180 mL) was vigorously stirred at 110 °C for 2 h. Then the mixture was cooled to room temperature, the solvent was removed under reduced pressure, the residue was taken up in dichloromethane, filtered over Celite® and washed with DCM. The solvent was evaporated under reduced pressure, and the crude product was purified on a silica column by using a mixture of hexane:ethyl acetate 3:1 as eluent. Yield: 1.10 g, 21%. ^{1}H NMR (500 MHz, DMSO-d_6) δ, ppm: 8.64 (d, J = 2.4 Hz, 1H), 8.52 (d, J = 9.1 Hz, 1H), 8.39 (dd, J = 9.3, 2.5 Hz, 1H), 7.48 – 7.44 (m, 1H), 7.43 – 7.40 (m, 2H), 7.38 – 7.33 (m, 4H), 7.27 (d, J = 7.6 Hz, 1H), 4.01 – 3.94 (m, 1H), 3.42 (m, 1H), 2.81 (m, 1H), 2.35 (s, 3H), 2.21 (m, 1H), 1.19 (s, 9H). ESI-MS (acetonitrile/methanol + 1% water), positive: m/z 584.22 $[M + Na]^+$.

Species e$_2$. Under argon atmosphere a mixture of **d$_2$** (4.39 g, 6.60 mmol), triphenylphosphine (0.87 g, 3.30 mmol), palladium(II) acetate (0.37 g, 1.65 mmol) and silver(I) carbonate (4.55 g, 16.50 mmol) was vigorously stirred in dry dimethylformamide (100 mL) at 80 °C for 2 h and then cooled to room temperature. The solvent was removed under reduced pressure, the residue was taken up in DCM, filtered over Celite® and washed with DCM. The filtrate was evaporated under reduced pressure, and the crude product was purified on a silica column by using a mixture of hexane:ethyl acetate 3:1 as eluent. Yield: 2.05 g, 54%. ^{1}H NMR (500 MHz, DMSO-d_6) δ, ppm: 8.27 (d, J = 8.5 Hz, 1H), 7.82 (d, J = 7.5 Hz, 1H), 7.52 (dd, J = 11.5, 4.3 Hz, 1H), 7.46 – 7.41 (m, 2H), 7.39 (d, J = 8.5 Hz, 2H), 7.34 (m, 4H), 7.28 (d, J = 7.6 Hz, 1H), 4.01 – 3.92 (m, 1H), 2.90 – 2.82 (m, 2H), 2.33 (s, 3H), 2.31 (m, 1H), 1.17 (d, J = 3.8 Hz, 9H). ESI-MS (acetonitrile/methanol + 1% water), positive: m/z 539.24 $[M + Na]^+$.

Species e$_3$. Under argon atmosphere a mixture of **d$_3$** (7.22 g, 9.98 mmol), triphenylphosphine (1.30 g, 4.99 mmol), palladium(II) acetate (0.56 g, 2.50 mmol) and silver(I) carbonate (6.86 g, 24.95 mmol) was vigorously stirred in dry

dimethylformamide (180 mL) at 85 °C for 2 h and then was cooled to room temperature. The solvent was removed under reduced pressure, the residue was taken up in DCM, filtered over Celite® and washed with DCM. The filtrate was evaporated under reduced pressure, and the crude product was purified on a silica column by using a mixture of hexane:ethyl acetate 3:1 as eluent. Yield: 2.61 g, 44%. ^{1}H NMR (500 MHz, DMSO) *δ*, ppm: 8.24 (d, *J* = 9.0 Hz, 1H), 7.95 (d, *J* = 2.0 Hz, 1H), 7.69 (dd, *J* = 9.0, 2.1 Hz, 1H), 7.46 – 7.42 (m, 1H), 7.37 – 7.32 (m, 6H), 7.27 (d, *J* = 7.6 Hz, 1H), 3.96 (m, 1H), 2.82 (m, 1H), 2.34 (m, 4H), 2.22 (m, 1H), 1.17 (s, 9H). ESI-MS (acetonitrile/methanol + 1% water), positive: *m/z* 617.13 $[M + Na]^+$.

Species f$_1$. To **e$_1$** (1.10 g, 1.96 mmol) in DCM (48 mL) was added trifluoroacetic acid (10 mL) and the solution was stirred at room temperature for 2 h. Then water (73 mL) was added, and the intermediate species was extracted with DCM (3 × 30 mL). The combined organic phases were evaporated under reduced pressure to afford a crude solid. To a solution of the crude solid in absolute THF (40 mL) tetra-*n*-butylammonium fluoride (9.8 mL) was added and the reaction mixture was refluxed for 1 h. The solvent was removed on a rotary evaporator and the product crystallized in methanol to give a light green solid. Yield: 540 mg, 90%. ^{1}H NMR (500 MHz, DMSO) *δ*, ppm: 12.77 (s, 1H), 8.97 (d, *J* = 2.3 Hz, 1H), 8.18 – 8.09 (m, 2H), 7.89 (d, *J* = 7.6 Hz, 1H), 7.68 (d, *J* = 9.0 Hz, 1H), 7.61 – 7.55 (m, 1H), 7.53 (m, 2H), 4.15 (d, *J* = 5.3 Hz, 2H), 3.17 (d, *J* = 4.9 Hz, 2H). ESI-MS (acetonitrile/methanol + 1% water), positive: *m/z* 330.12 $[M + Na]^+$.

Species f2. To **e$_2$** (5.77 g, 11.18 mmol) in DCM (270 mL) was added trifluoroacetic acid (56 mL) and the mixture stirred at room temperature for 2 h. Water (250 mL) was added and the intermediate species was extracted with DCM (3 × 200 mL). The combined organic phases were concentrated in vacuo to afford a crude solid. To a solution of the crude solid in absolute THF (51.1 mL) tetra-*n*-butylammonium fluoride (20.4 mL) was added and the mixture was refluxed for 1 h. Then the solvent was removed on a rotary evaporator and the product was purified by column chromatography with DCM containing 0.5% MeOH as eluent to give a white solid. Yield: 520 mg, 54%. ^{1}H NMR (500 MHz, DMSO) *δ*, ppm: 11.70 (s, 1H), 7.69 (d, *J* = 7.9 Hz, 1H), 7.43 – 7.36 (m, 5H), 7.31 (t, *J* = 4.8 Hz, 1H), 7.19 – 7.14 (m, 1H), 7.11 – 7.06 (m, 1H), 3.50 – 3.43 (m, 2H), 3.04 (t, *J* = 6.9 Hz, 2H). X-ray diffraction quality single crystals were obtained by slow evaporation of a methanolic solution of **f$_2$**.

Species f_3. To a solution of $\mathbf{e_3}$ (0.98 g, 1.65 mmol) in DCM (40 mL) was added trifluoroacetic acid (8 mL) and the mixture stirred at room temperature for 2 h. Then water (60 mL) was added, and the intermediate species was extracted with DCM (3 × 40 mL). The combined organic phases were concentrated in vacuo to afford a crude solid. To a solution of the crude solid in absolute THF (37.5 mL) tetra-*n*-butylammonium fluoride (8.1 mL) was added and the mixture was refluxed for 1 h. The solvent was removed under reduced pressure and purified on silica column by using DCM containing 0.5% MeOH as eluent to give a white solid. Yield: 433 mg, 77%. ^{1}H NMR (500 MHz, DMSO) *δ*, ppm: 11.94 (s, 1H), 7.82 (d, *J* = 1.9 Hz, 1H), 7.43 – 7.37 (m, 6H), 7.29 (dd, *J* = 8.6, 2.0 Hz, 1H), 3.46 (m, 2H), 3.04 (t, *J* = 6.9 Hz, 2H). ESI-MS (acetonitrile/methanol + 1% water), positive: *m/z* 363.10 $[M + Na]^+$.

Species g_1. A pressurization vessel containing a suspension of $\mathbf{f_1}$ (540 mg, 1.76 mmol) and palladium (10 %) on activated charcoal (187 mg, 0.18 mmol Pd) in absolute THF (88 mL) was flushed with hydrogen (3 × 4 bar) and then filled with hydrogen (4 bar). The reaction mixture was stirred at room temperature overnight. On the next day, the suspension was filtered over Celite®, the filtrate was evaporated to give the product. Yield: 460 mg, 94%. ^{1}H NMR (500 MHz, DMSO) *δ*, ppm: 11.23 (s, 1H), 7.35 (m, 4H), 7.10 (m, 2H), 6.89 (d, *J* = 2.0 Hz, 1H), 6.54 (dd, *J* = 8.5, 2.2 Hz, 1H), 4.62 (s, 2H), 3.44 (dd, J = 11.7, 6.9 Hz, 2H), 3.01 (t, *J* = 6.9 Hz, 2H). ESI-MS (acetonitrile/methanol + 1% water), positive: *m/z* 300.11 $[M + Na]^+$.

Species g_2. In a Schlenk tube under argon atmosphere to a solution of **f2** (1.70 g, 6.49 mmol) in absolute THF (80 mL) a mixture of phosphorus(V) pentasulfide and basic aluminium oxide (0.6 : 1 w/w)[50] (4.68 g) was added and the reaction mixture was stirred at 75 °C overnight. On the next day the mixture was cooled to room temperature and filtered. The filtrate was evaporated under reduced pressure and the residue purified on silica column by using DCM with 1% MeOH as eluent to give a yellow solid. Yield: 1.32 mg, 73%. ^{1}H NMR (500 MHz, DMSO) *δ*, ppm: 11.82 (s, 1H), 10.13 (t, *J* = 6.2 Hz, 1H), 7.95 (d, *J* = 8.0 Hz, 1H), 7.42 (d, *J* = 8.1 Hz, 1H), 7.39 – 7.33 (m, 4H), 7.19 – 7.15 (m, 1H), 7.12 – 7.07 (m, 1H), 3.66 (br d, *J* = 124.6 Hz, 2H), 3.18 (d, *J* = 6.4 Hz, 2H). ESI-MS (acetonitrile/methanol + 1% water), positive: *m/z* 279.13 $[M + H]^+$. X-ray diffraction quality single crystals were obtained by slow evaporation of a methanolic solution of $\mathbf{g_2}$.

Species g_3. In a Schlenk tube under argon to a solution of **f3** (433 mg, 1.27 mmol) in absolute THF (21 mL) was added a mixture of phosphorus(V) pentasulfide and basic

aluminium oxide (0.6 : 1 w/w)[50] (0.92 g) and the reaction mixture was stirred at 75 °C overnight. On the next day, the mixture was cooled to room temperature and filtered. The filtrate was evaporated on a rotary evaporator to give a residue, which was purified by column chromatography with DCM containing 1% MeOH as eluent. The product was isolated as yellow powder. Yield: 170 mg, 37%. ^{1}H NMR (500 MHz, DMSO) *δ*, ppm: 12.06 (s, 1H), 10.23 (t, *J* = 6.2 Hz, 1H), 8.08 (d, *J* = 1.9 Hz, 1H), 7.38 (m, 5H), 7.30 (dd, *J* = 8.6, 2.0 Hz, 1H), 3.69 (br d, *J* = 120.0 Hz, 2H), 3.18 (m, 2H). ESI-MS (acetonitrile/methanol + 1% water), positive: *m/z* 357.12 $[M + H]^+$.

Species h_2. Hydrazine hydrate (17.64 mL) was added to a suspension of **g_2** (800 mg, 2.93 mmol) in chloroform (133 mL) and the reaction mixture was refluxed for 3 h, cooled to room temperature, diluted with water (100 mL) and extracted with chloroform (3 × 100 mL). The organic phases were combined, dried over magnesium sulfate and concentrated under reduced pressure to afford the desired product as white powder. Yield: 833 mg, 98%. ^{1}H NMR (500 MHz, DMSO) *δ*, ppm: 11.31 (s, 1H), 8.14 (d, *J* = 8.0 Hz, 1H), 7.63 – 7.59 (m, 1H), 7.43 – 7.34 (m, 5H), 7.15 – 7.10 (m, 1H), 7.05 – 7.01 (m, 1H), 5.65 (br s, 2H), 3.92 (br s, 1H), 3.17 (br s, 2H), 2.77 (t, *J* = 6.0 Hz, 2H). ESI-MS (acetonitrile/methanol + 1% water), positive: *m/z* 277.15 $[M + H]^+$.

Species h_3. Hydrazine hydrate (2.00 mL) was added to a suspension of **g_3** (120 mg, 0.34 mmol) in chloroform (13 mL). The reaction mixture was refluxed for 3 h, cooled to room temperature, diluted with water (15 mL) and extracted with chloroform (3 × 20 mL). The organic phases were combined, dried over magnesium sulfate and concentrated under reduced pressure to afford the desired product as white powder. Yield: 120 mg, 99%. ^{1}H NMR (500 MHz, DMSO) *δ*, ppm: 11.52 (br s, 1H), 7.60 (m, 1H), 7.44 – 7.41 (m, 2H), 7.38 (m, 2H), 7.32 (d, *J* = 8.6 Hz, 1H), 7.25 (dd, *J* = 8.5, 2.0 Hz, 1H), 5.75 (br s, 2H), 3.92 (br s, 1H), 3.16 (br s, 3H), 2.76 (t, *J* = 5.5 Hz, 2H).

^{1}H NMR spectra of intermediate species **b_1**, **b_2**, **c_1–c_3**, **d_1–d_3**, **e_1–e_3**, **f_1–f_3**, **g_1–g_3**, **h_1**, **h_2** are shown in Figures S36–S54.

X-ray crystallography data

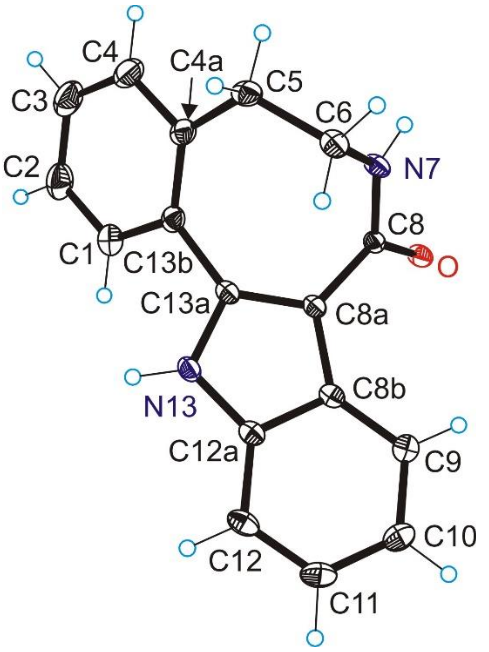

Figure S1. ORTEP view of the complex **f₂** with thermal ellipsoids at 50% probability level.

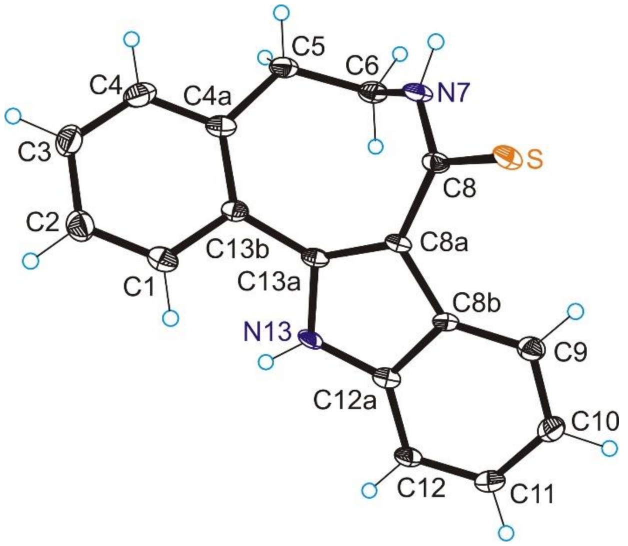

Figure S2. ORTEP view of the complex **g₂** with thermal ellipsoids at 50% probability level.

NMR spectra of **HL^1–HL^5**

All ^{13}C NMR spectra (except ^{13}C-CPD for **HL^2**, Figure S6 in the SI) are ^{13}C-DEPTq spectra where CH and CH_3 are shown as positive (upwards) signals, while C_q and CH_2 as negative (downwards) ones.

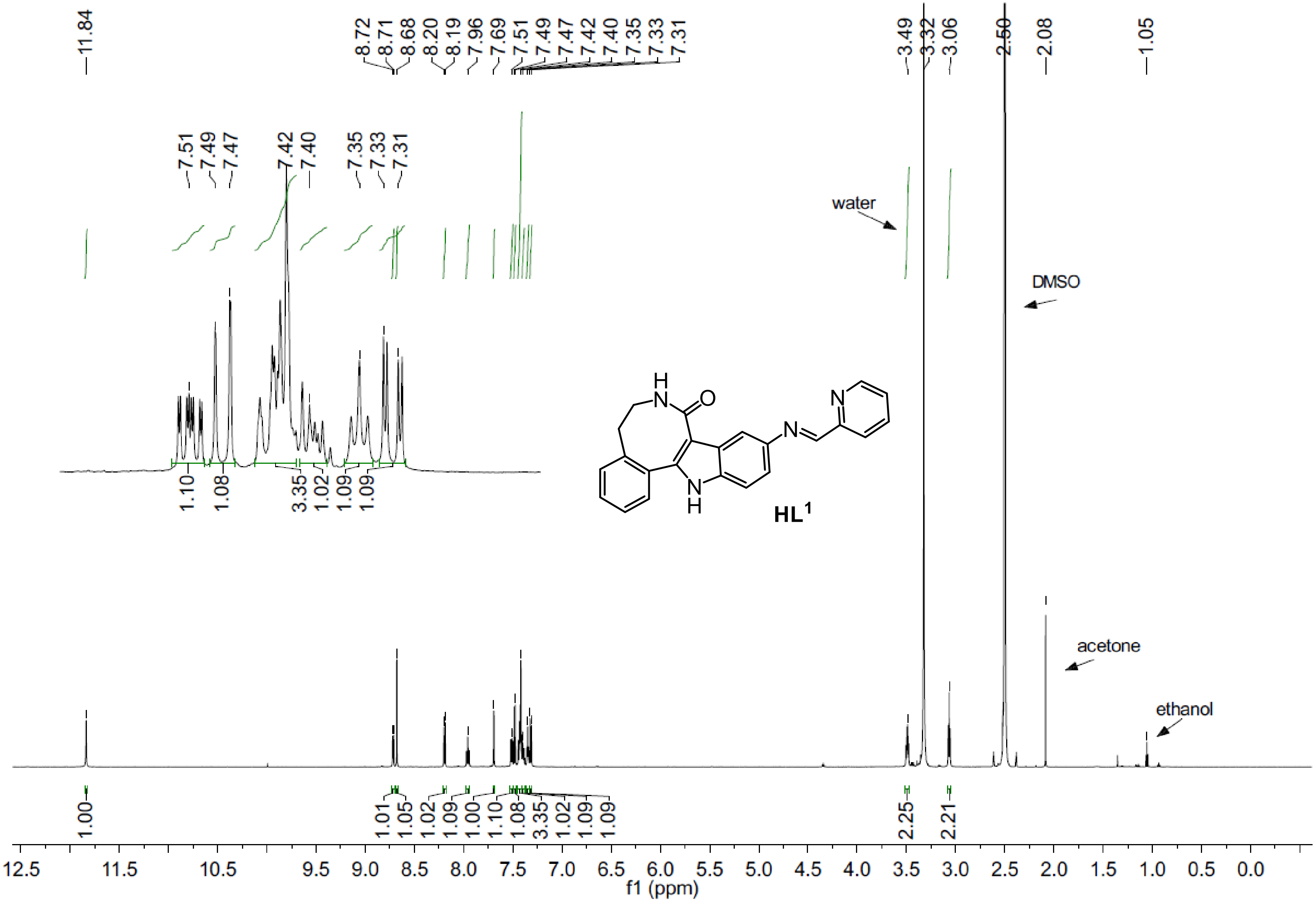

Figure S3. ^{1}H NMR spectrum of **HL[1]** in DMSO (600 MHz).

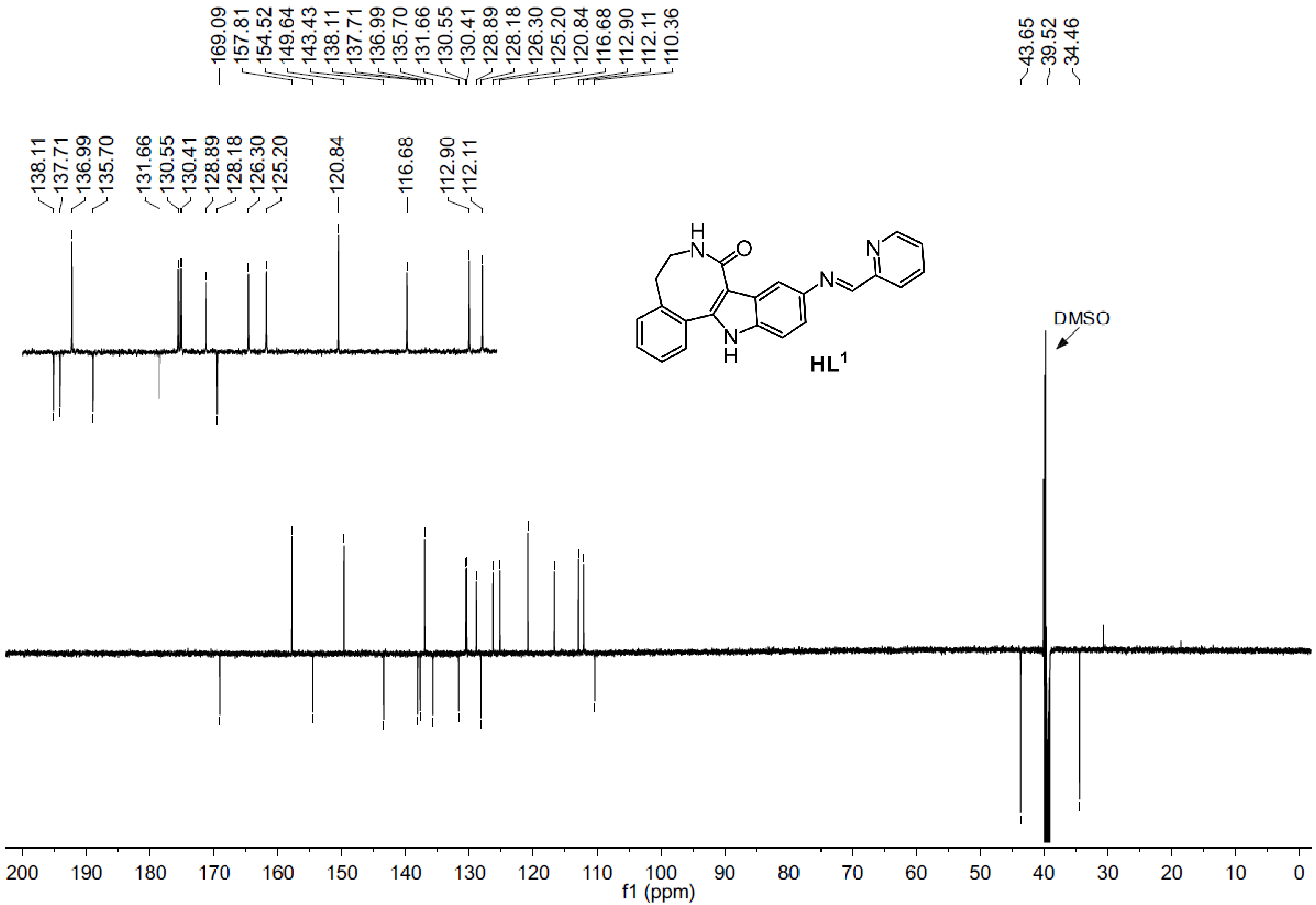

Figure S4. ^{13}C-DEPTq NMR spectrum of **HL^{1}** in DMSO (151 MHz).

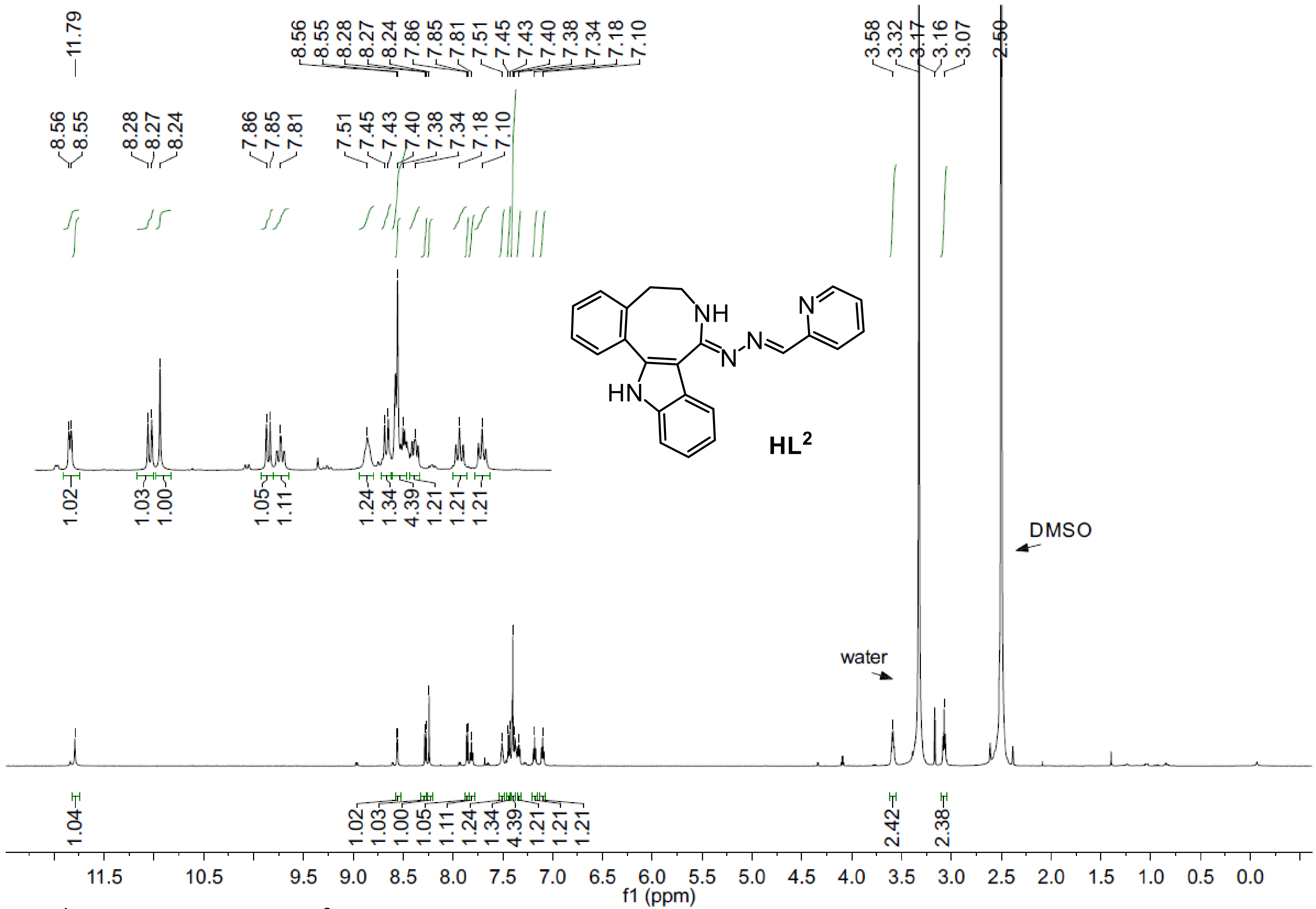

Figure S5. ^{1}H NMR spectrum of **HL^2** in DMSO (700 MHz).

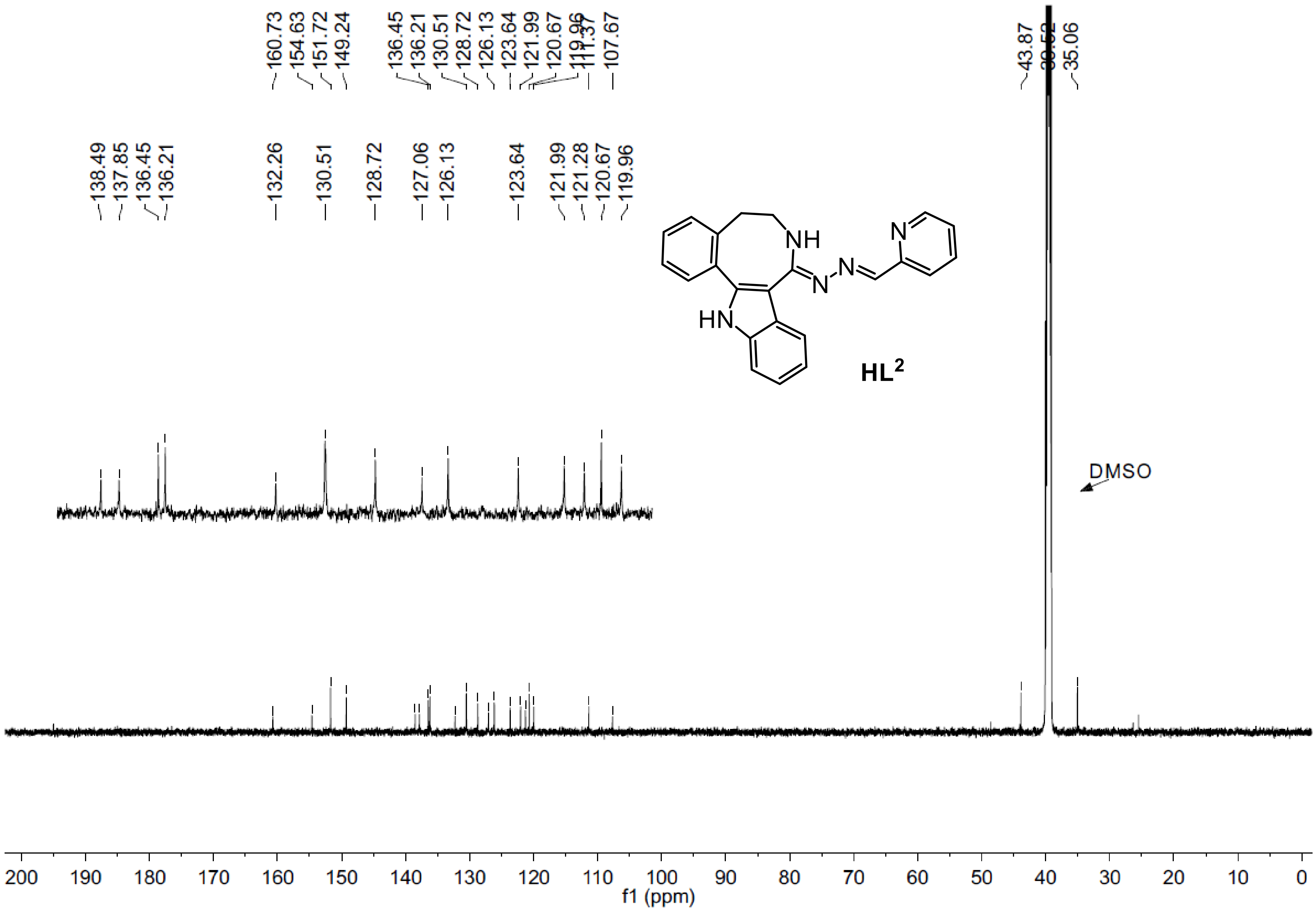

Figure S6. ^{13}C-CPD NMR spectrum of **HL^2** in DMSO (151 MHz).

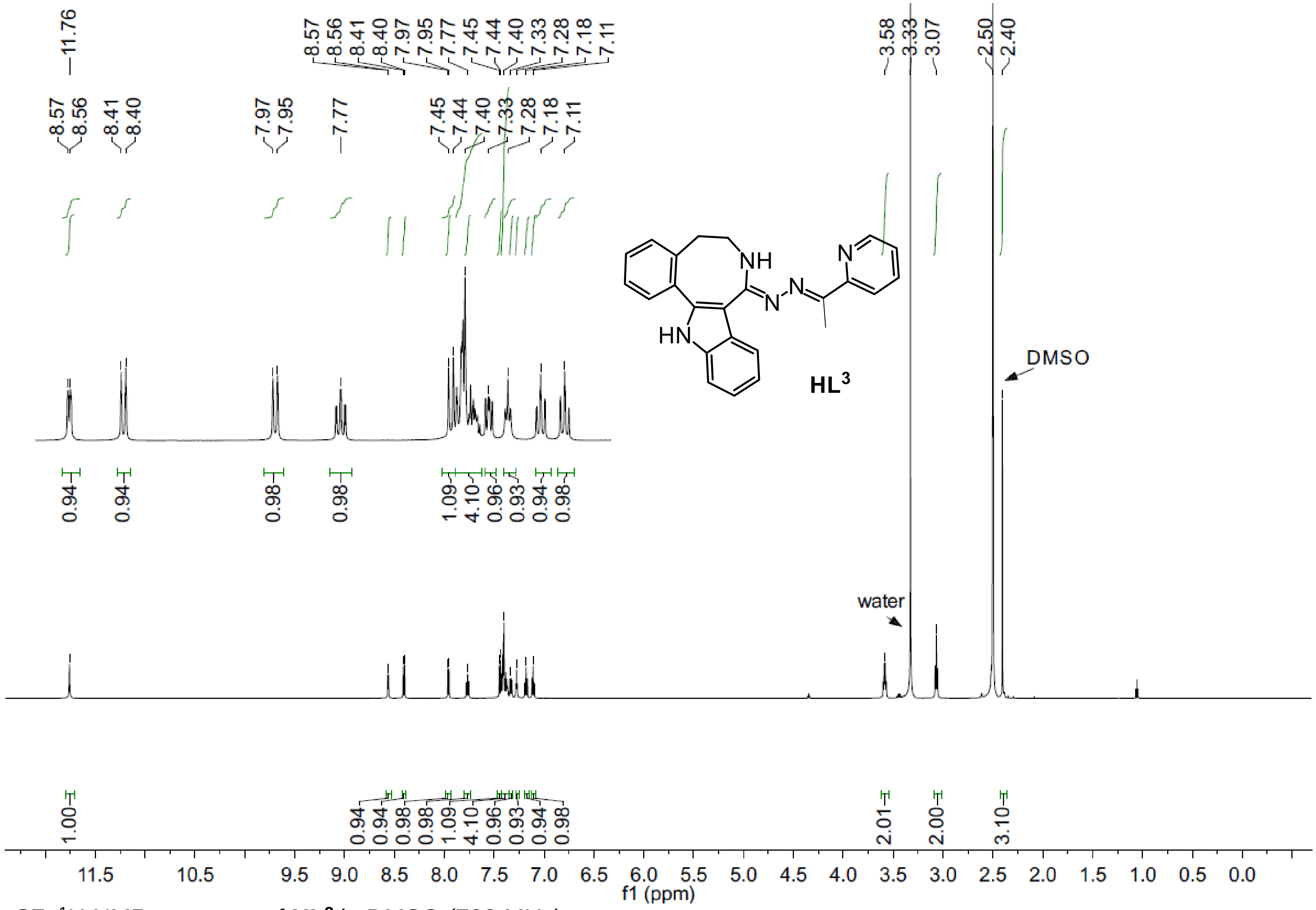

Figure S7. ^{1}H NMR spectrum of **HL^{3}** in DMSO (700 MHz).

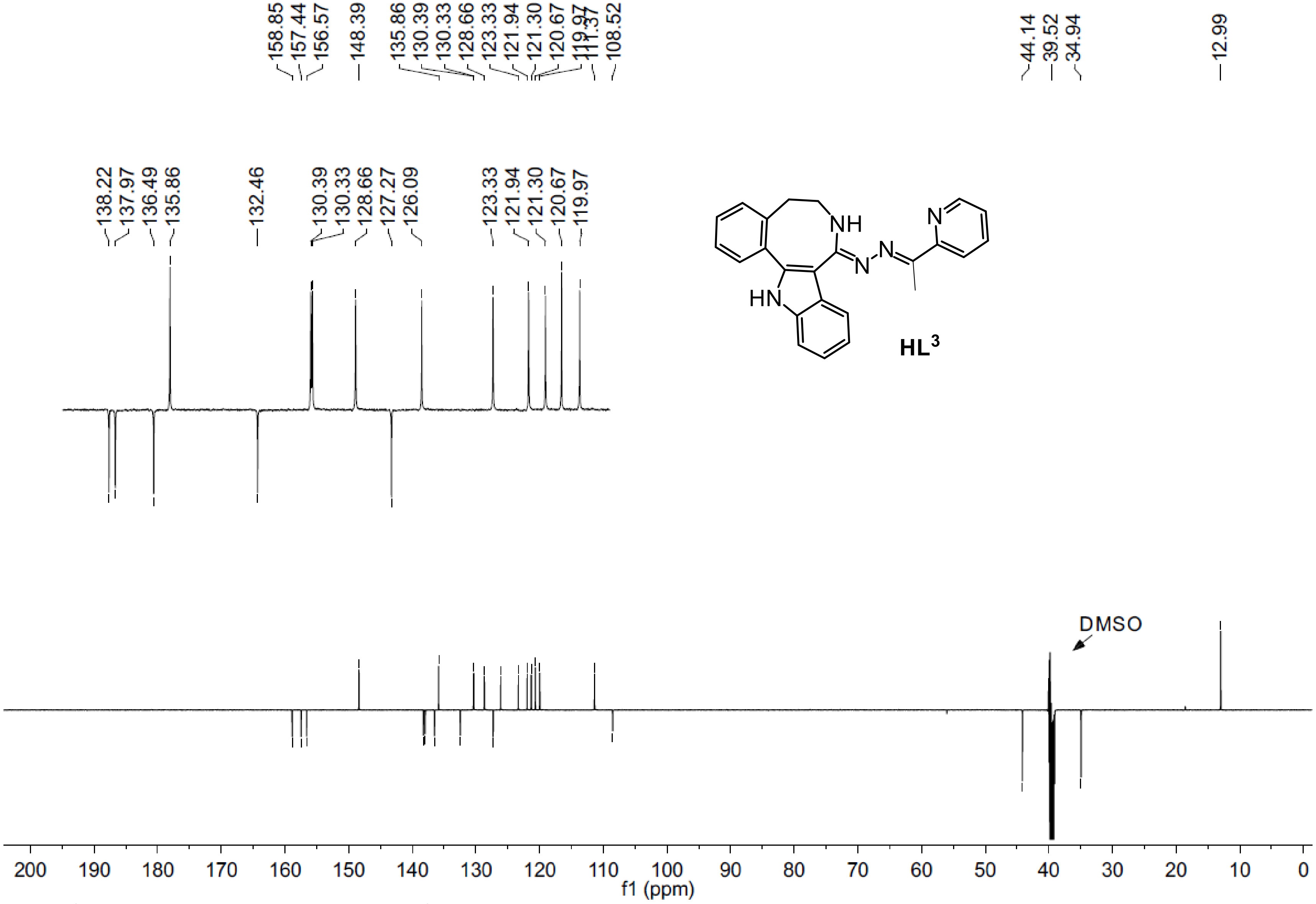

Figure S8. ^{13}C-DEPTq NMR spectrum of **HL^{3}** in DMSO (151 MHz).

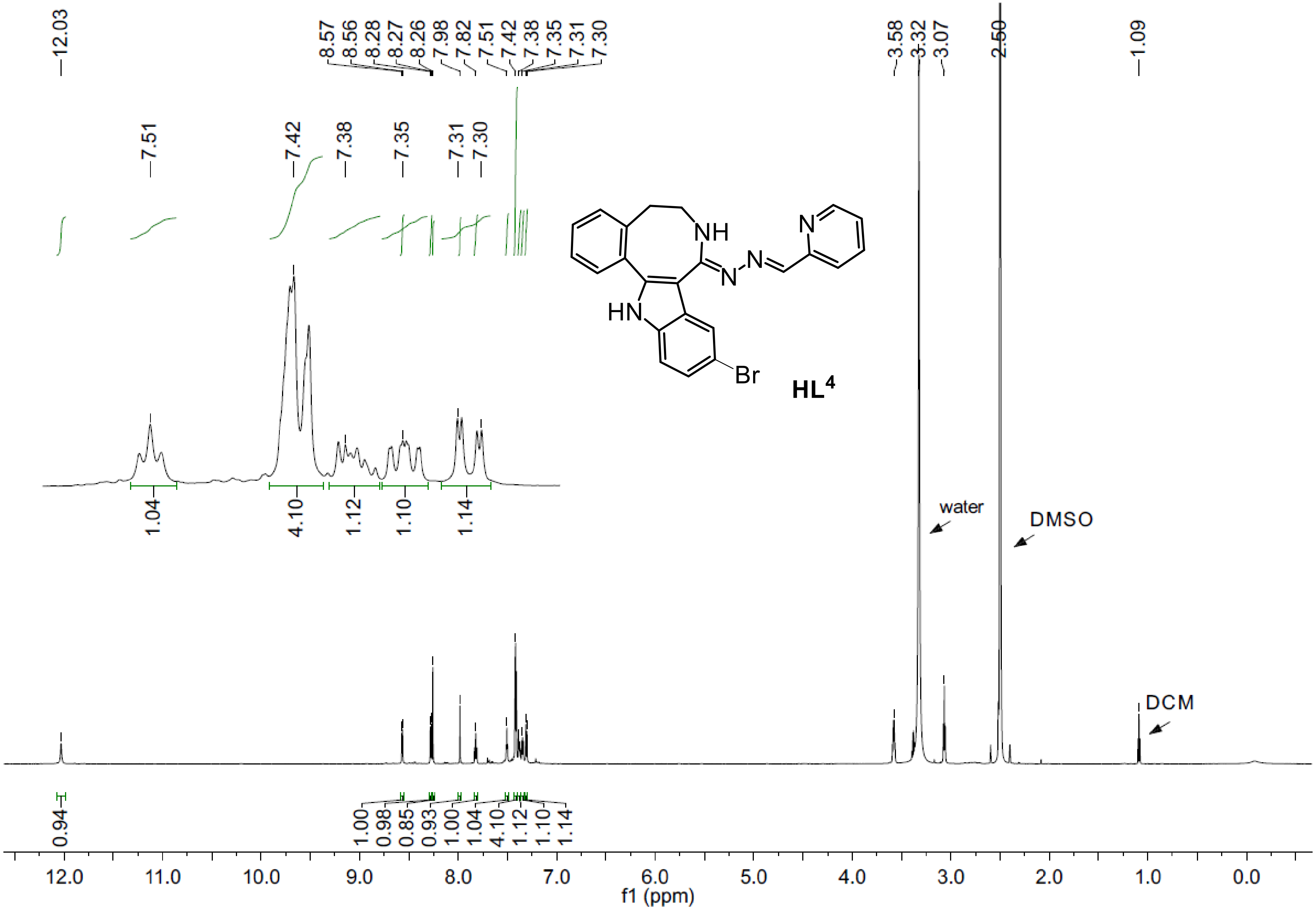

Figure S9. ^{1}H NMR spectrum of **HL^{4}** in DMSO (600 MHz).

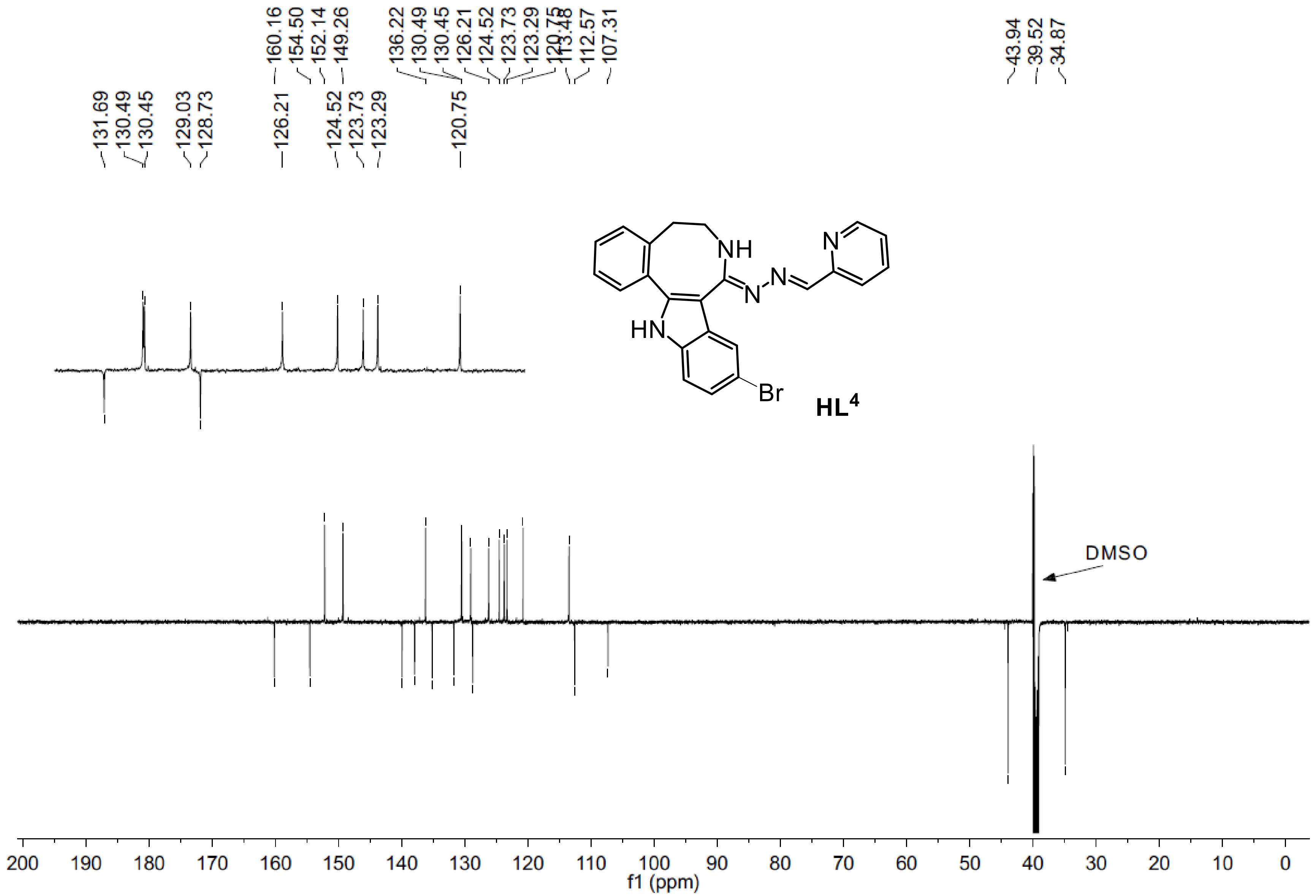

Figure S10. ^{13}C-DEPTq NMR spectrum of **HL^4** in DMSO (151 MHz).

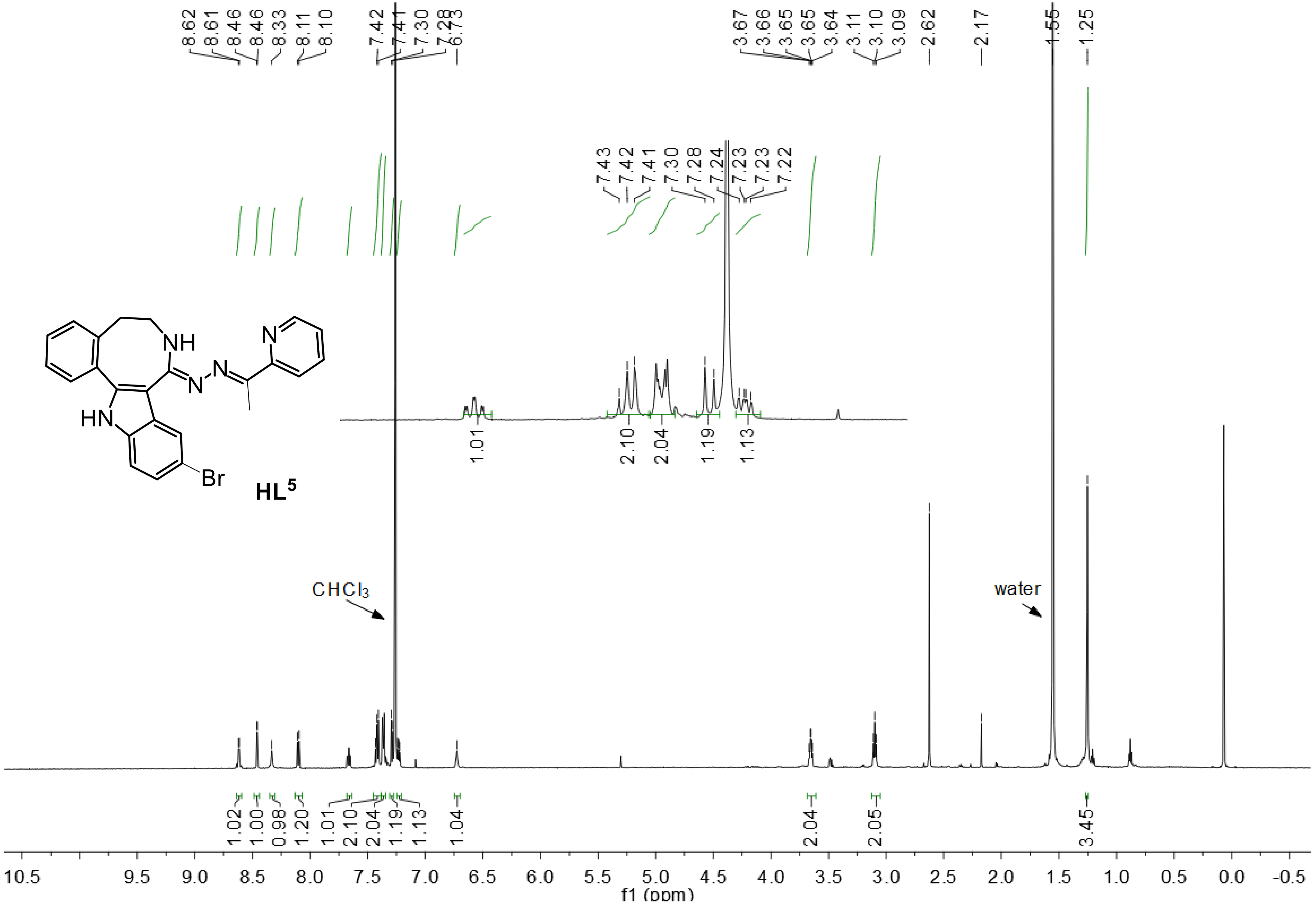

Figure S11. ^{1}H NMR spectrum of **HL⁵** in DMSO (700 MHz).

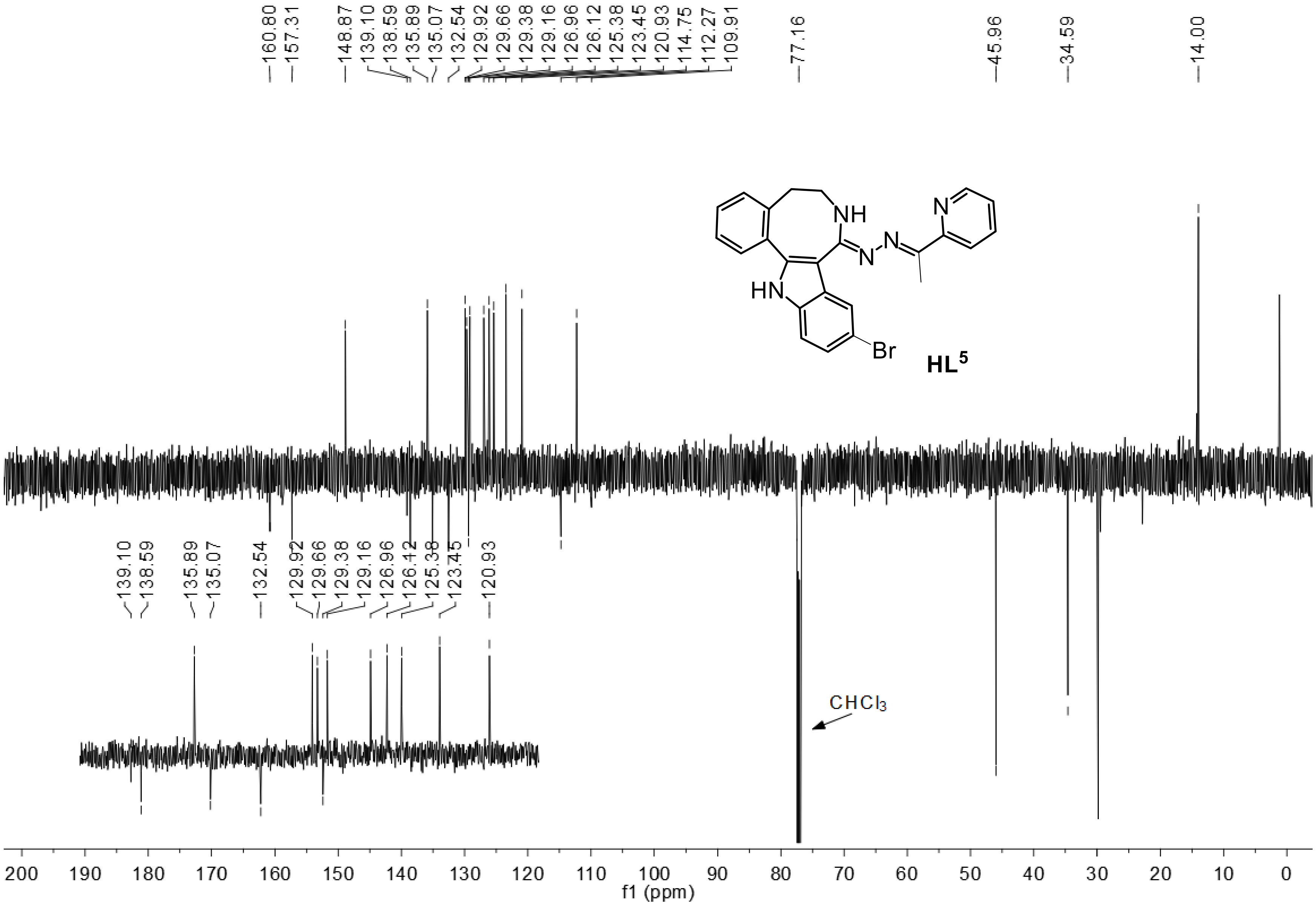

Figure S12. ^{13}C-DEPTq NMR spectrum of **HL^5** in $CDCl_3$ (151 MHz).

ESI-MS spectra of **HL^2–HL^5**, **1a**,**1b**, **2–5**

Generic Display Report

Analysis Info

Analysis Name	D:\Data\MS_MessService\77749_IRKU501_amazon.d	Acquisition Date	3/1/2021 12:54:21 PM
Method	MSC-Service_direct-injection.m	Operator	MSC
Sample Name	77749_IRKU501_amazon	Instrument	amaZon speed ETD
Comment	Kuznetcova / Anorg.Chem.<br>ACN / MeOH + 1 % H2O		

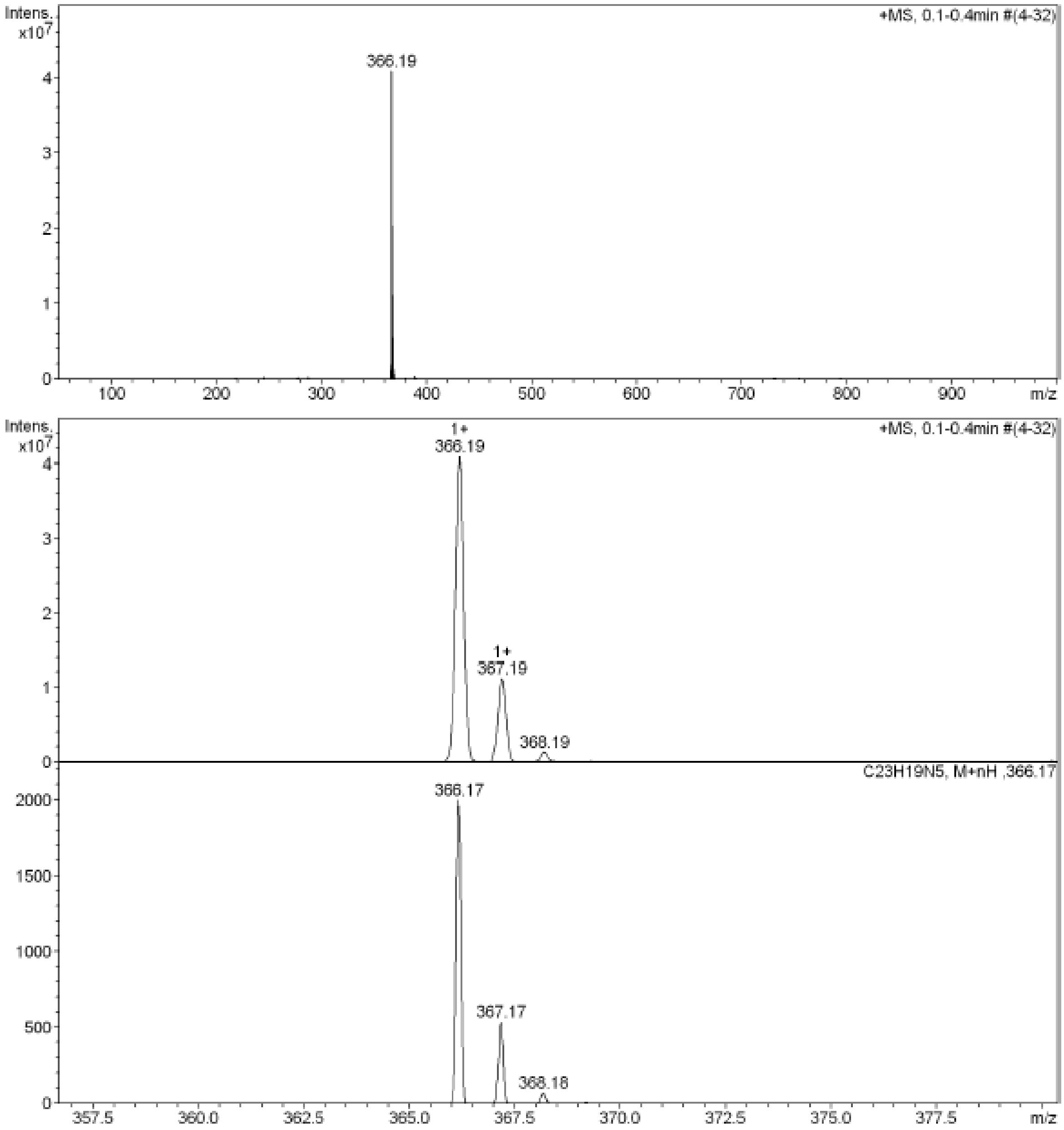

Bruker Compass DataAnalysis 4.0 printed: 3/1/2021 12:57:23 PM Page 1 of 1

Figure S13. ESI mass spectrum of **HL^2**.

Generic Display Report

**Analysis Info**		Acquisition Date	3/1/2021 12:46:36 PM
Analysis Name	D:\Data\MS_MessService\77748_IRKU500_amazon.d		
Method	MSC-Service_direct-injection.m	Operator	MSC
Sample Name	77748_IRKU500_amazon	Instrument	amaZon speed ETD
Comment	Kuznetcova / Anorg.Chem.<br>ACN / MeOH + 1 % H2O		

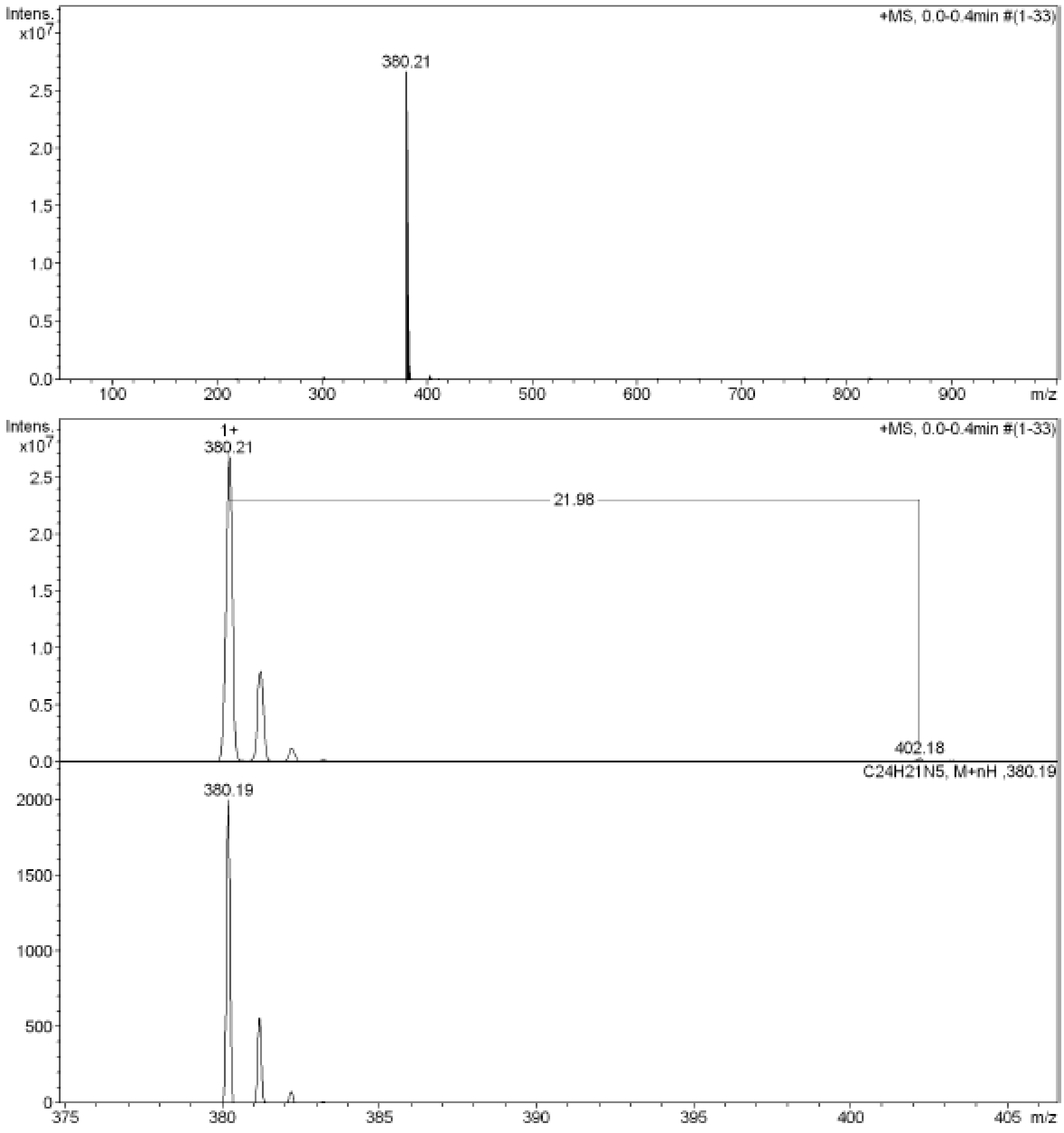

Bruker Compass DataAnalysis 4.0 printed: 3/1/2021 12:49:44 PM Page 1 of 1

Figure S14. ESI mass spectrum of **HL^3**.

Generic Display Report

Analysis Info

Analysis Name	D:\Data\MS_MessService\74895_IRKU393_amazon.d	Acquisition Date	11/17/2020 9:28:27 AM
Method	MSC-Service_direct-injection.m	Operator	MSC
Sample Name	74895_IRKU393_amazon	Instrument	amaZon speed ETD
Comment	Kuznetcova / AOC		
	ACN / MeOH 1% H2O		

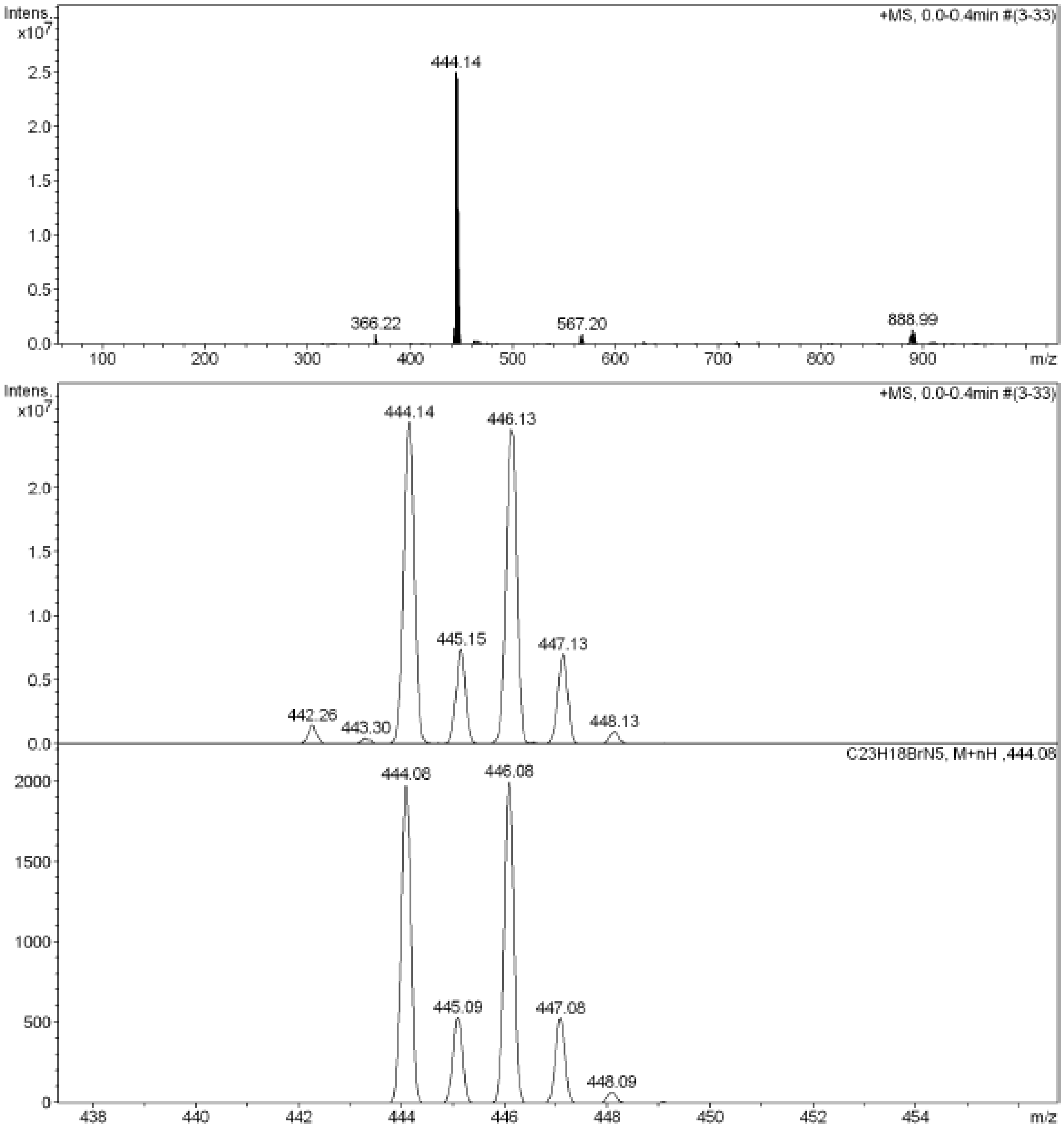

Bruker Compass DataAnalysis 4.0 printed: 11/17/2020 9:30:00 AM Page 1 of 1

Figure S15. ESI mass spectrum of **HL**^{4}.

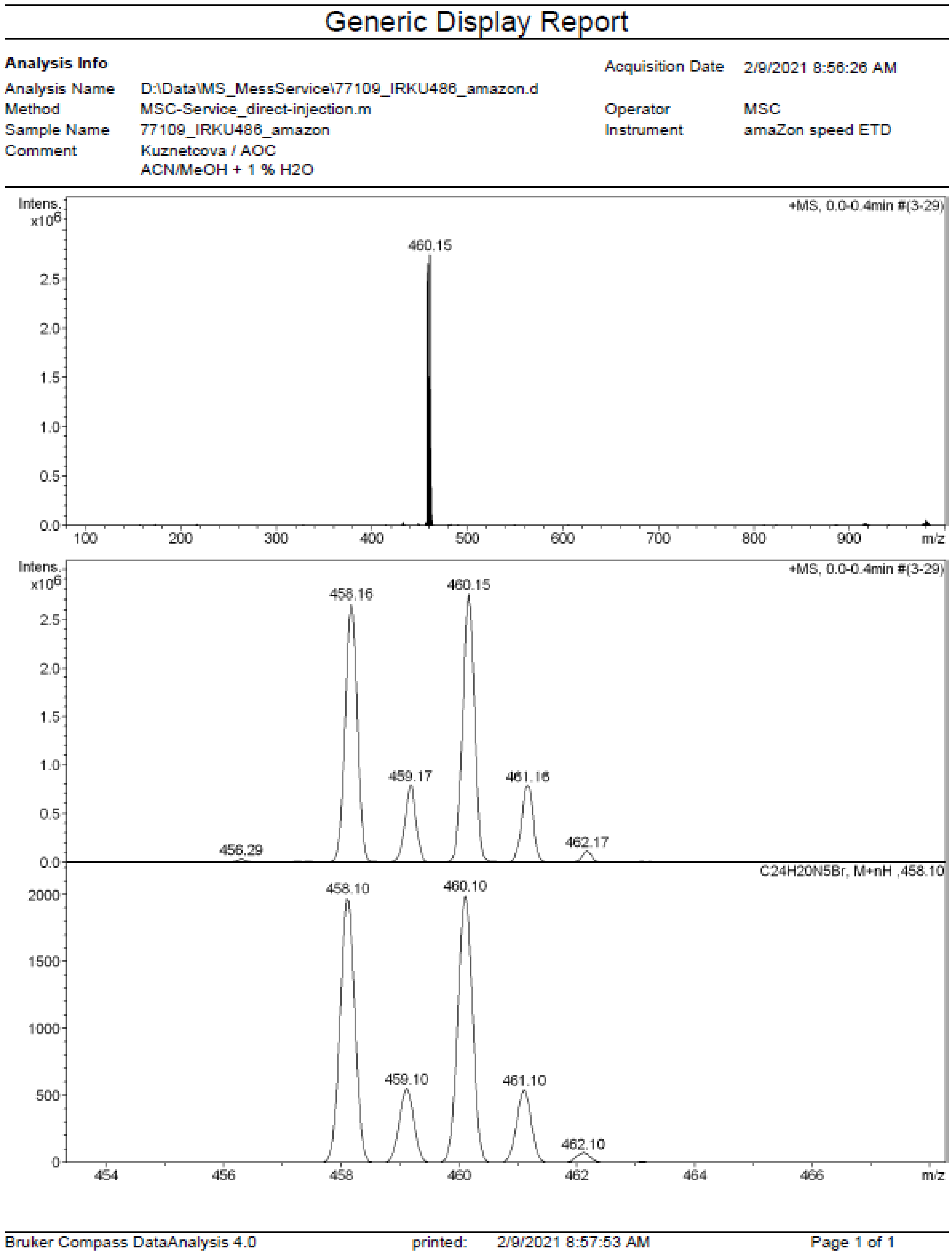

Figure S16. ESI mass spectrum of **HL**[5].

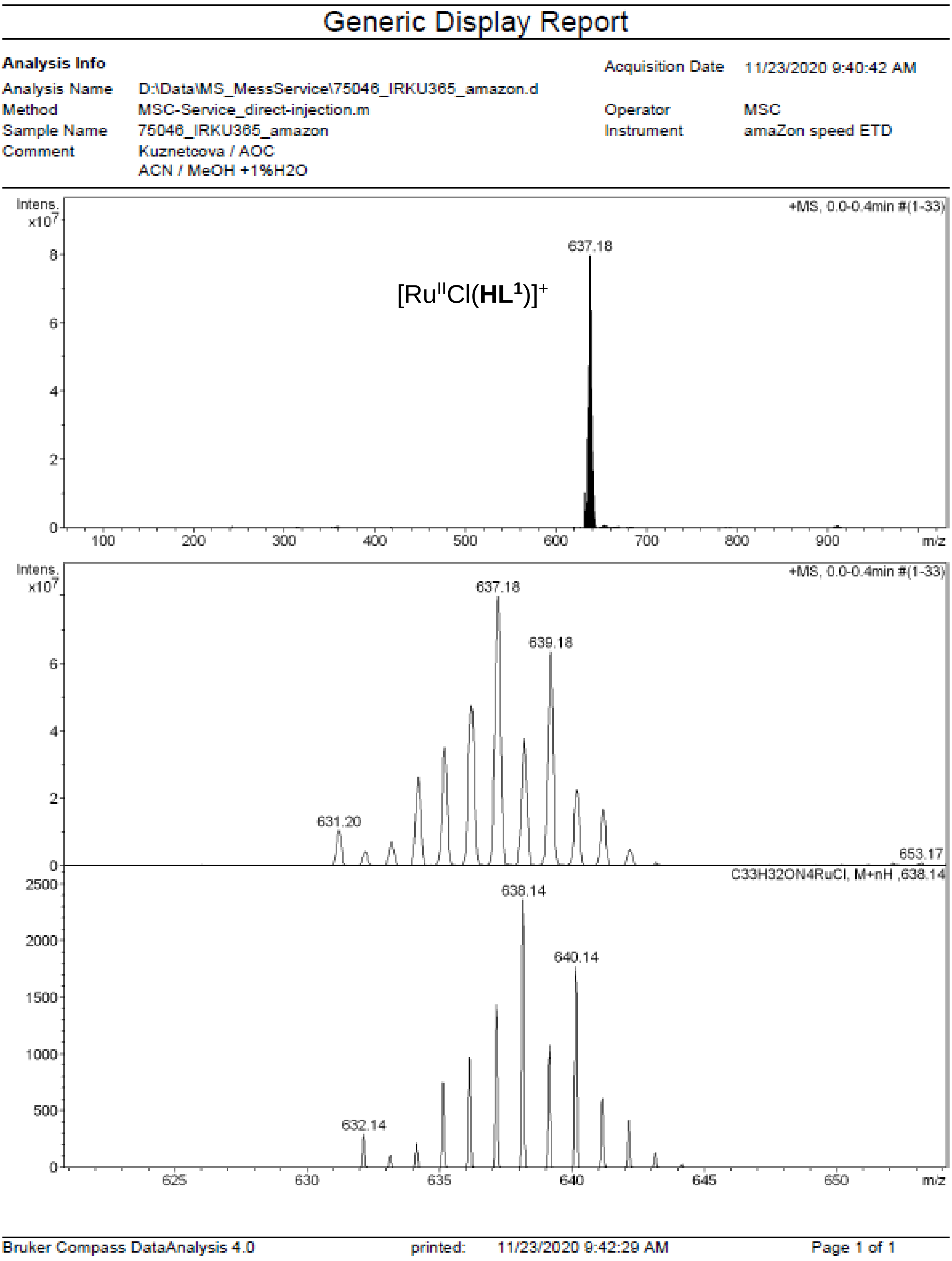

Figure S17. ESI mass spectrum of **1a**.

Generic Display Report

Analysis Info

Analysis Name	D:\Data\MS_MessService\78206_IRKU506_amazon.d	Acquisition Date	3/12/2021 1:56:21 PM
Method	MSC-Service_direct-injection.m	Operator	MSC
Sample Name	78206_IRKU506_amazon	Instrument	amaZon speed ETD
Comment	Kuznetkova / Anorg.Chem. ACN / MeOH + 1% H2O		

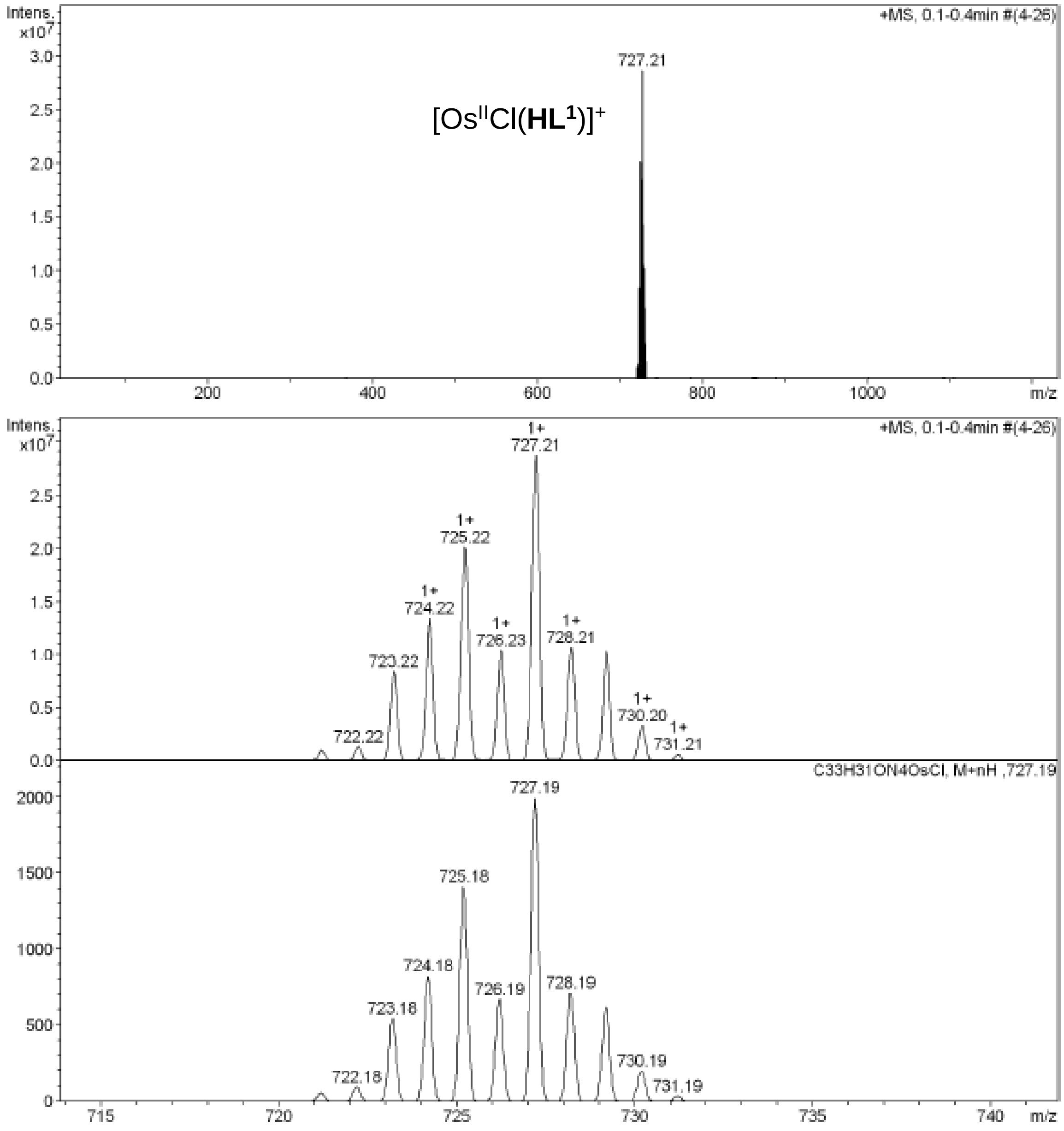

Bruker Compass DataAnalysis 4.0 printed: 3/12/2021 1:58:56 PM Page 1 of 1

Figure S18. ESI mass spectrum of **1b.**

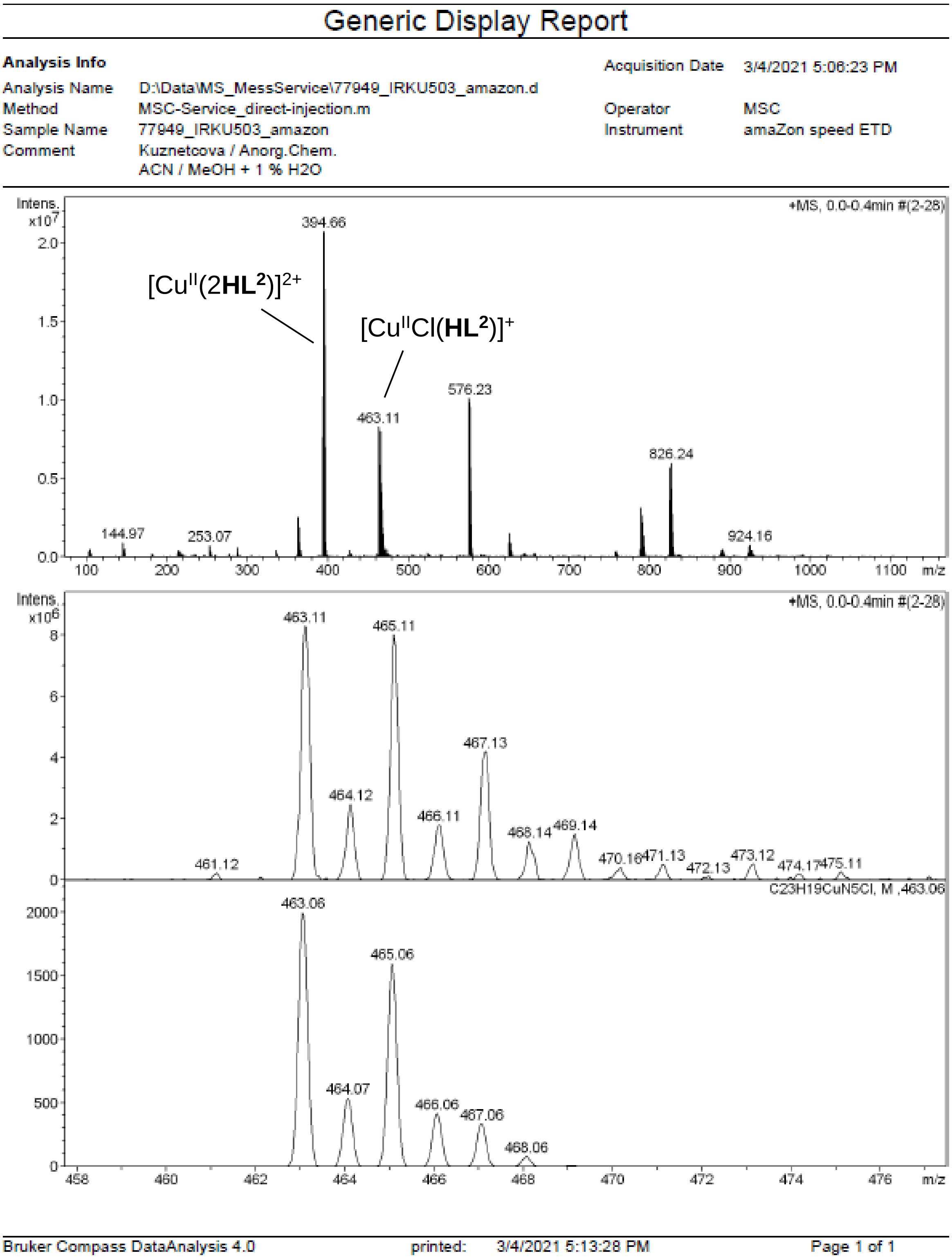

Figure S19. ESI mass spectrum of **2.**

Generic Display Report

Analysis Info			
Analysis Name	D:\Data\MS_MessService\77948_IRKU502_amazon.d	Acquisition Date	3/4/2021 4:48:40 PM
Method	MSC-Service_direct-injection.m	Operator	MSC
Sample Name	77948_IRKU502_amazon	Instrument	amaZon speed ETD
Comment	Kuznetcova / Anorg.Chem.<br>ACN / MeOH + 1 % H2O		

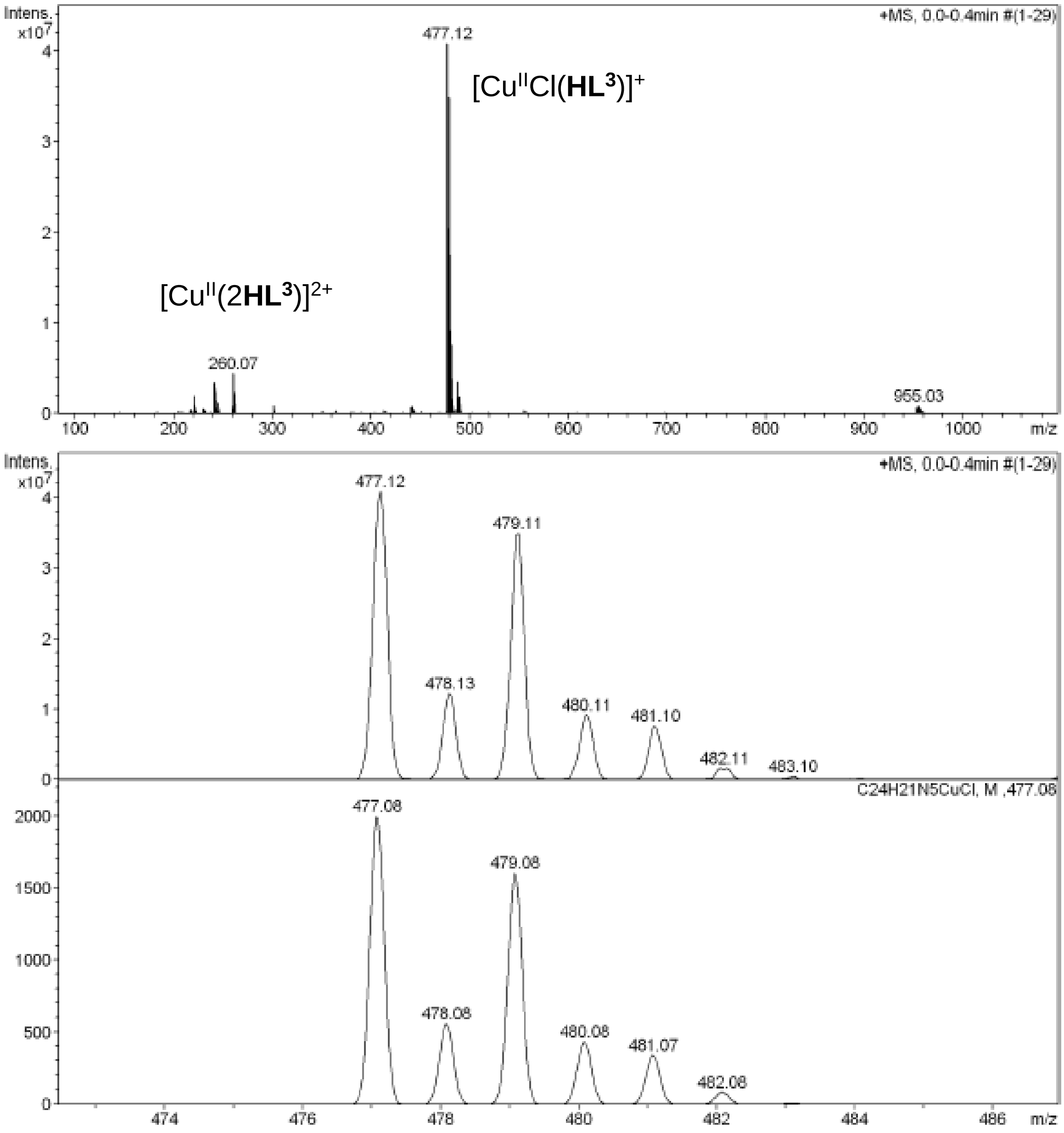

Bruker Compass DataAnalysis 4.0 printed: 3/4/2021 4:53:02 PM Page 1 of 1

Figure S20. ESI mass spectrum of **3.**

Generic Display Report

Analysis Info

Analysis Name	D:\Data\MS_MessService\75010_IRKU398_amazon.d	Acquisition Date	11/19/2020 1:22:41 PM
Method	MSC-Service_direct-injection.m	Operator	MSC
Sample Name	75010_IRKU398_amazon	Instrument	amaZon speed ETD
Comment	Kuzneteova / AOC<br>ACN / MeOH 1% H2O		

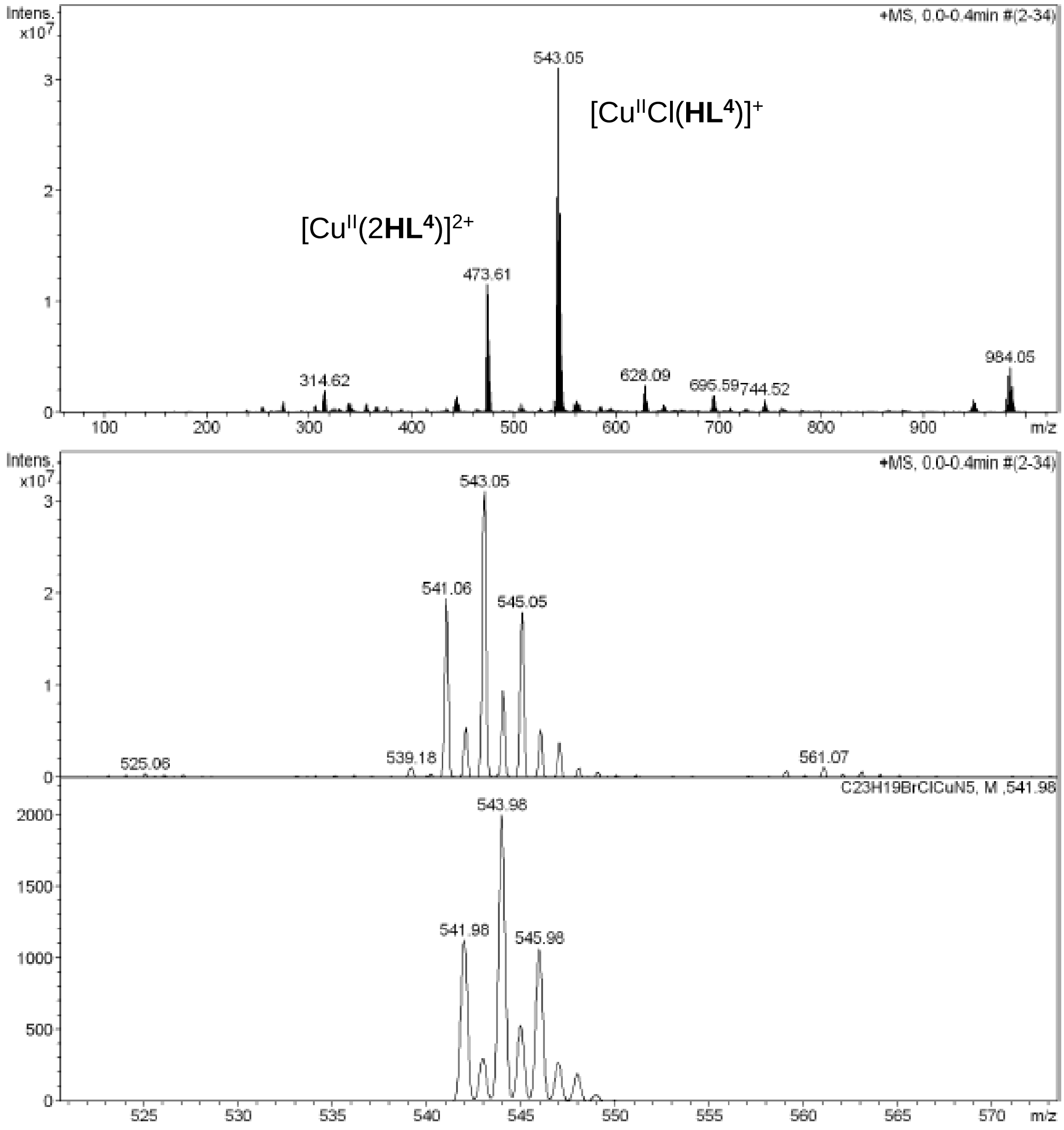

Bruker Compass DataAnalysis 4.0 printed: 11/19/2020 1:25:18 PM Page 1 of 1

Figure S21. ESI mass spectrum of **4.**

Generic Display Report

Analysis Info

Analysis Name	D:\Data\MS_MessService\77561_IRKU494_amazon.d	Acquisition Date	2/23/2021 6:06:57 PM
Method	MSC-Service_direct-injection.m	Operator	MSC
Sample Name	77561_IRKU494_amazon	Instrument	amaZon speed ETD
Comment	Kuznetcova / Anorg.Chem.		
	ACN / MeOH + 1 % H2O		

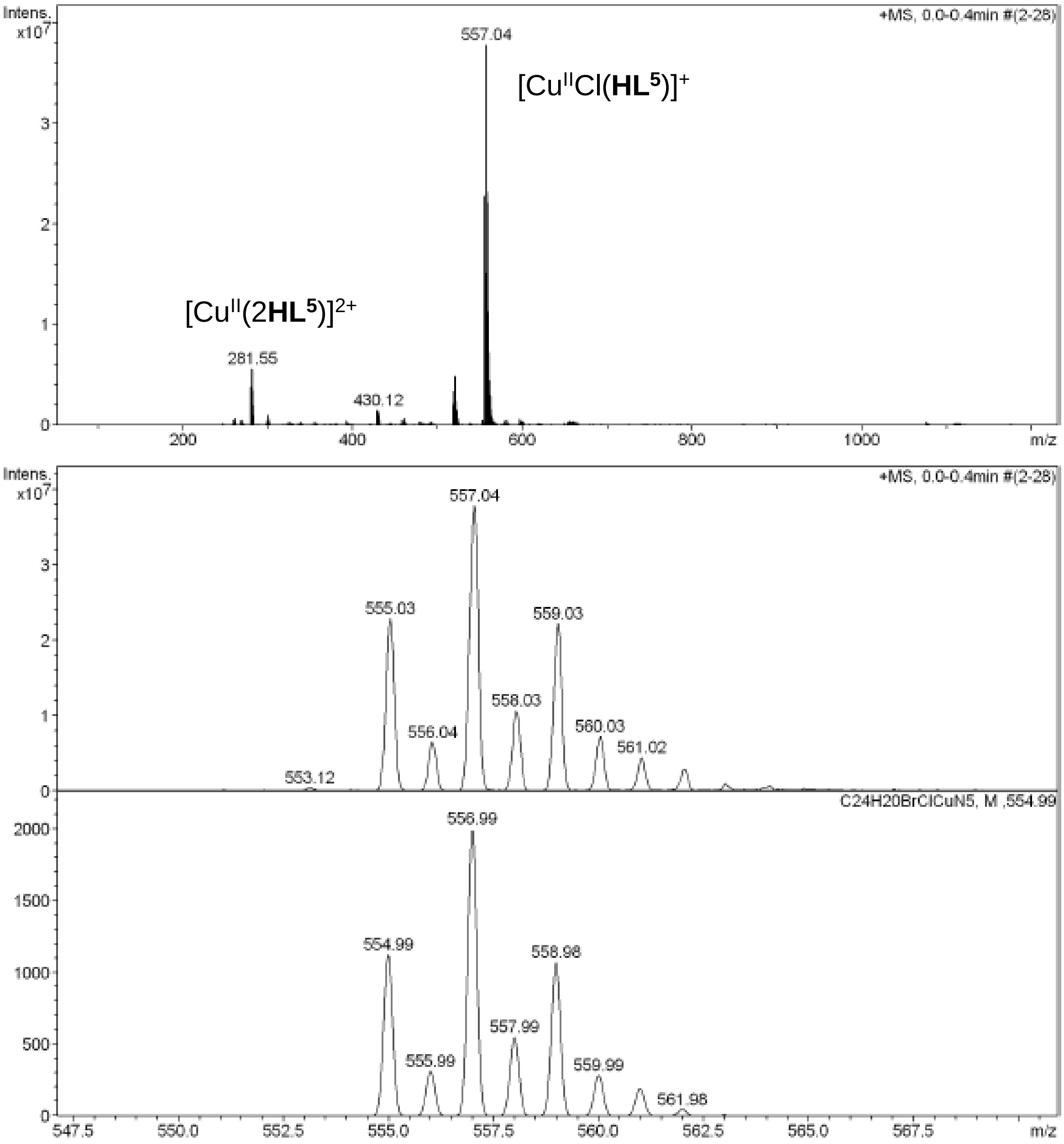

Bruker Compass DataAnalysis 4.0 printed: 2/23/2021 6:10:36 PM Page 1 of 1

Figure S22. ESI mass spectrum of **5.**

NMR spectra of **1a** and **1b**

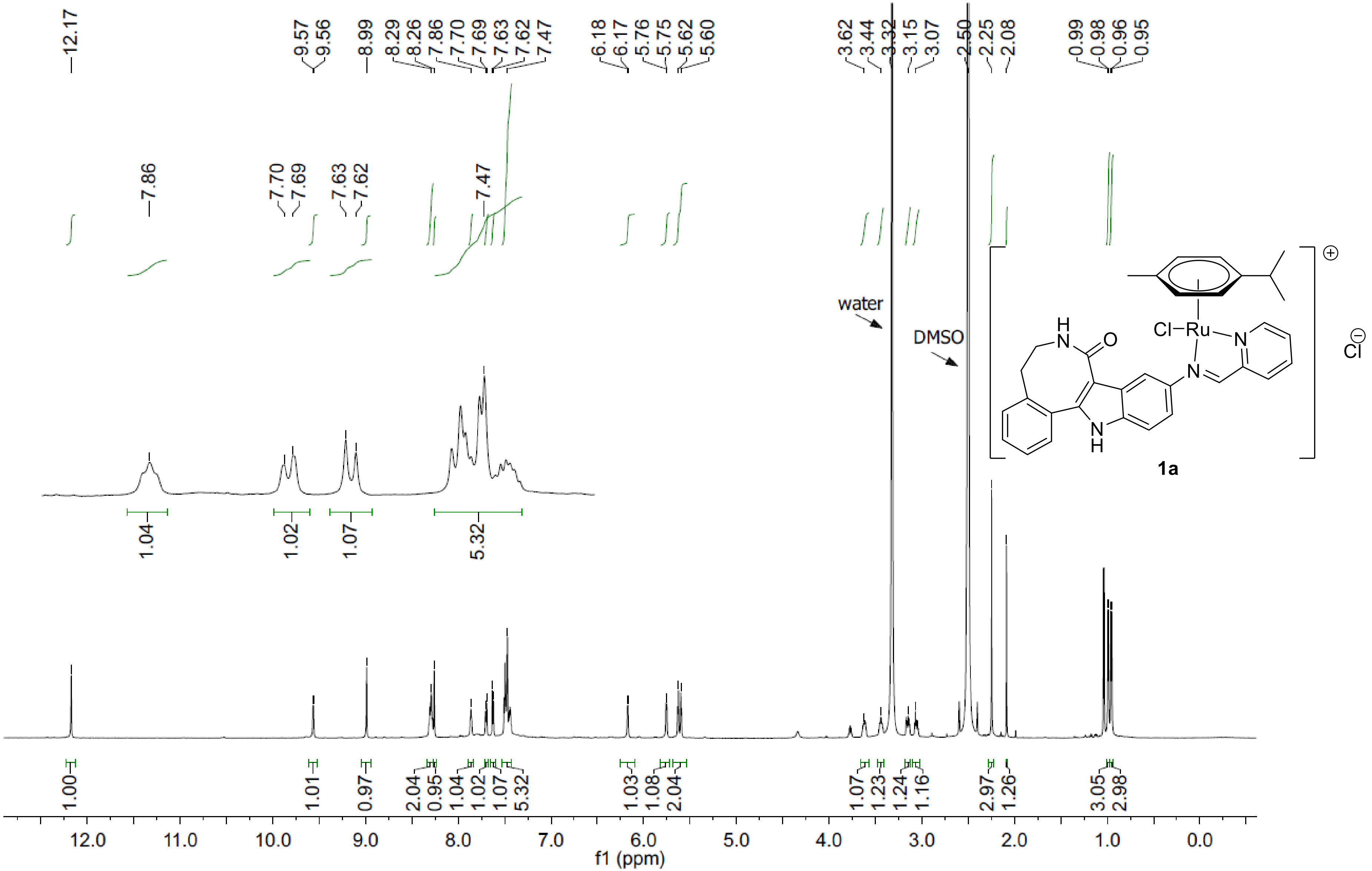

Figure S23. ^{1}H NMR spectrum of **1a** in DMSO (700 MHz).

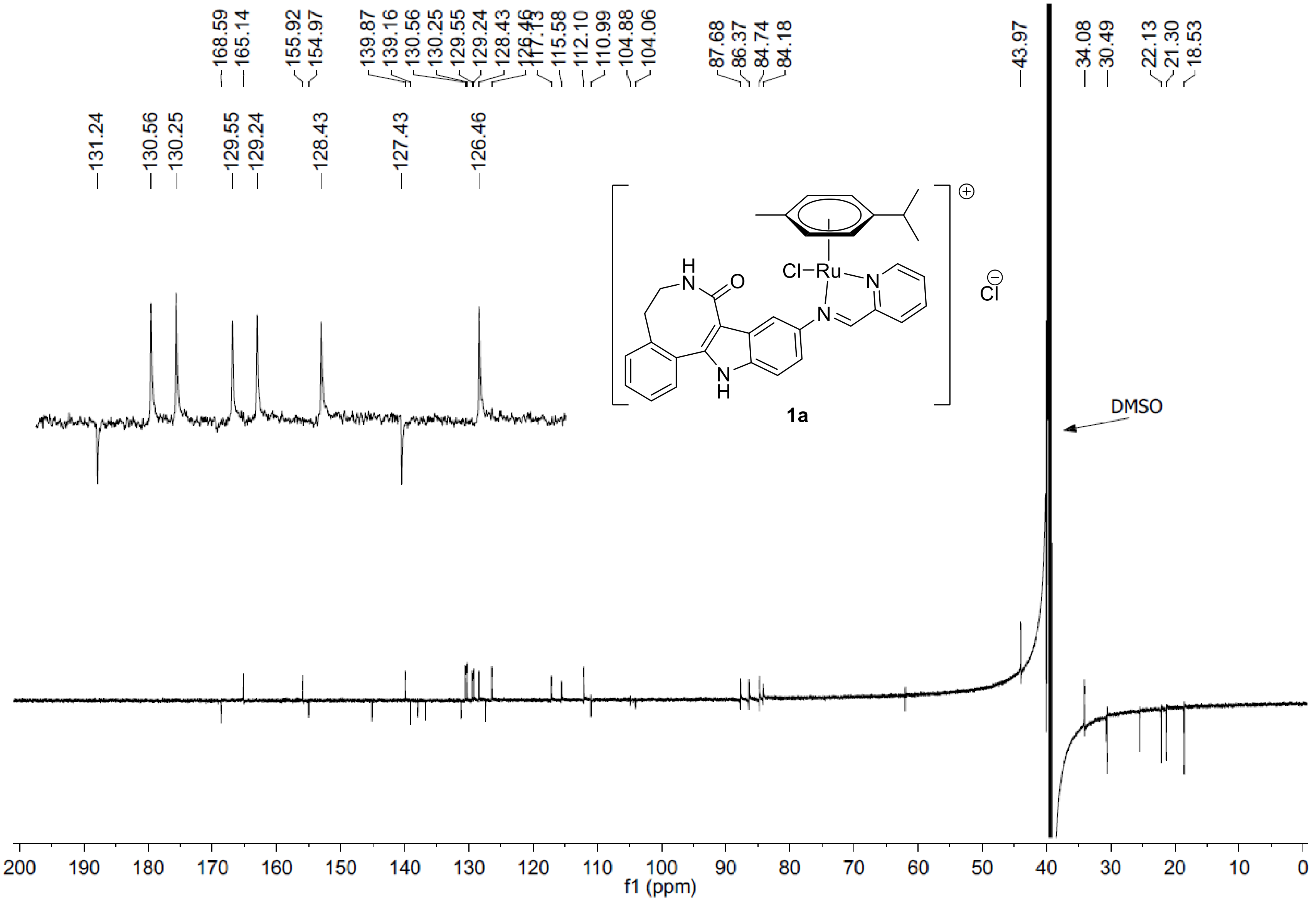

Figure S24. ^{13}C-DEPTq NMR spectrum of **1a** in DMSO (151 MHz).

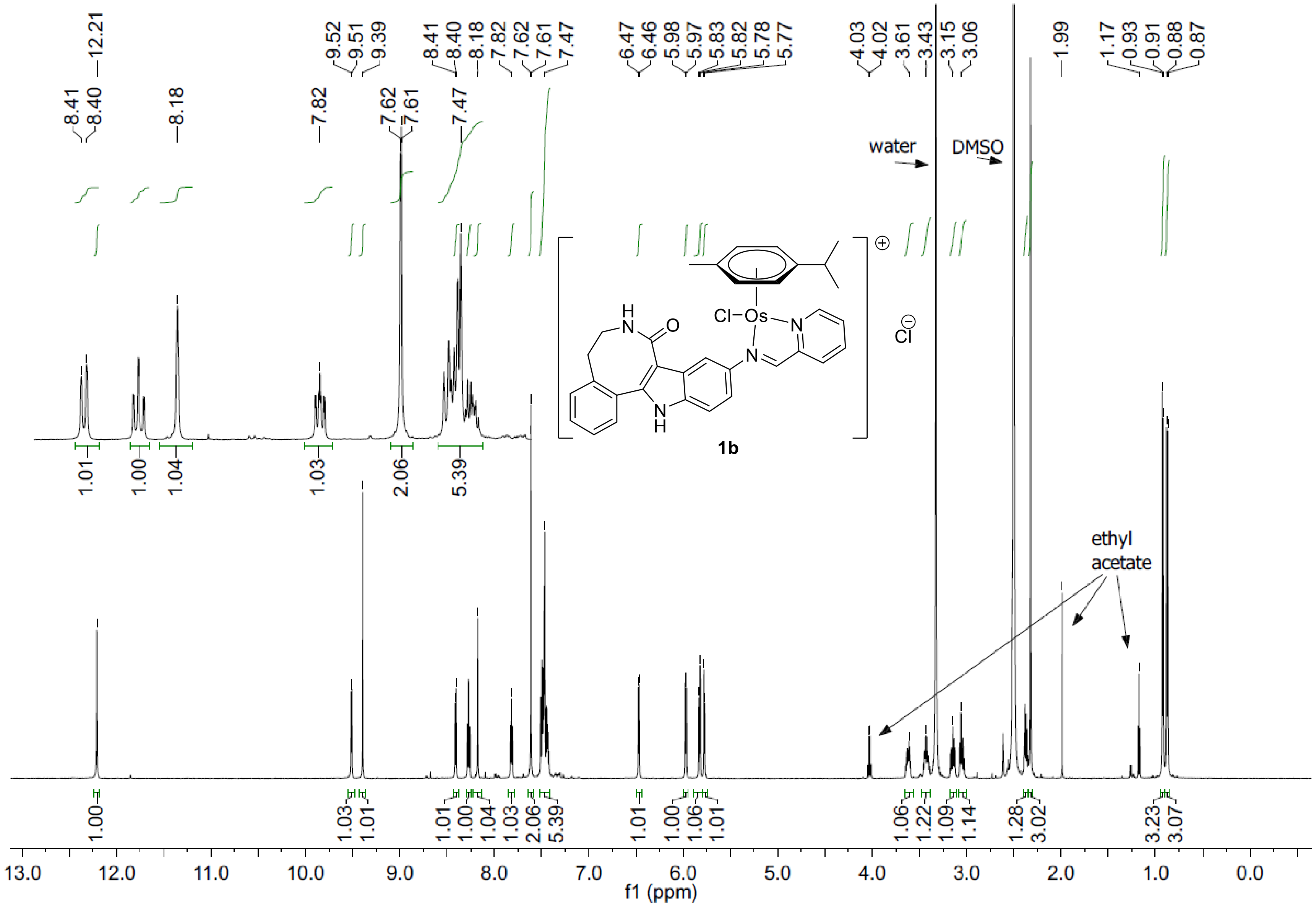

Figure S25. ^1H NMR spectrum of **1b** in DMSO (600 MHz).

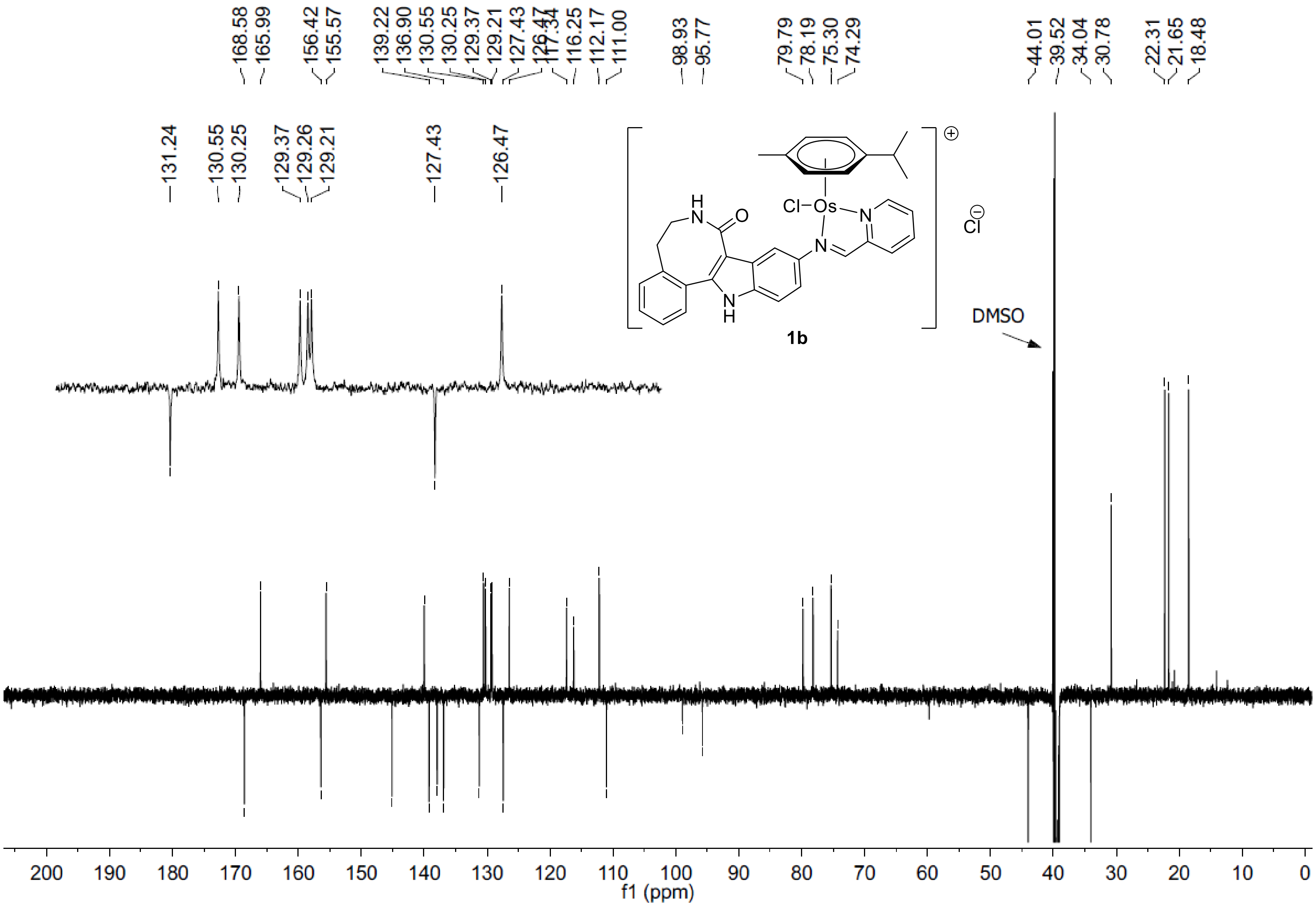

Figure S26. ^{13}C-DEPTq NMR spectrum of **1b** in DMSO (151 MHz).

High Performance Liquid Chromatography-MS

Generic Display Report

Analysis Info

Acquisition Date 24/11/2021 16:47:31

Analysis Name W:\MS_MessService\85178000001-85180_lc-ms_Wittmann\85179_IRKU486_12318.d

Method lc-ms1_r50-1900_isocrat50meoh-acn_300ulmin_10min_pos_wittmann.m Operator msc

Sample Name 85179_IRKU486 Instrument maXis

Comment

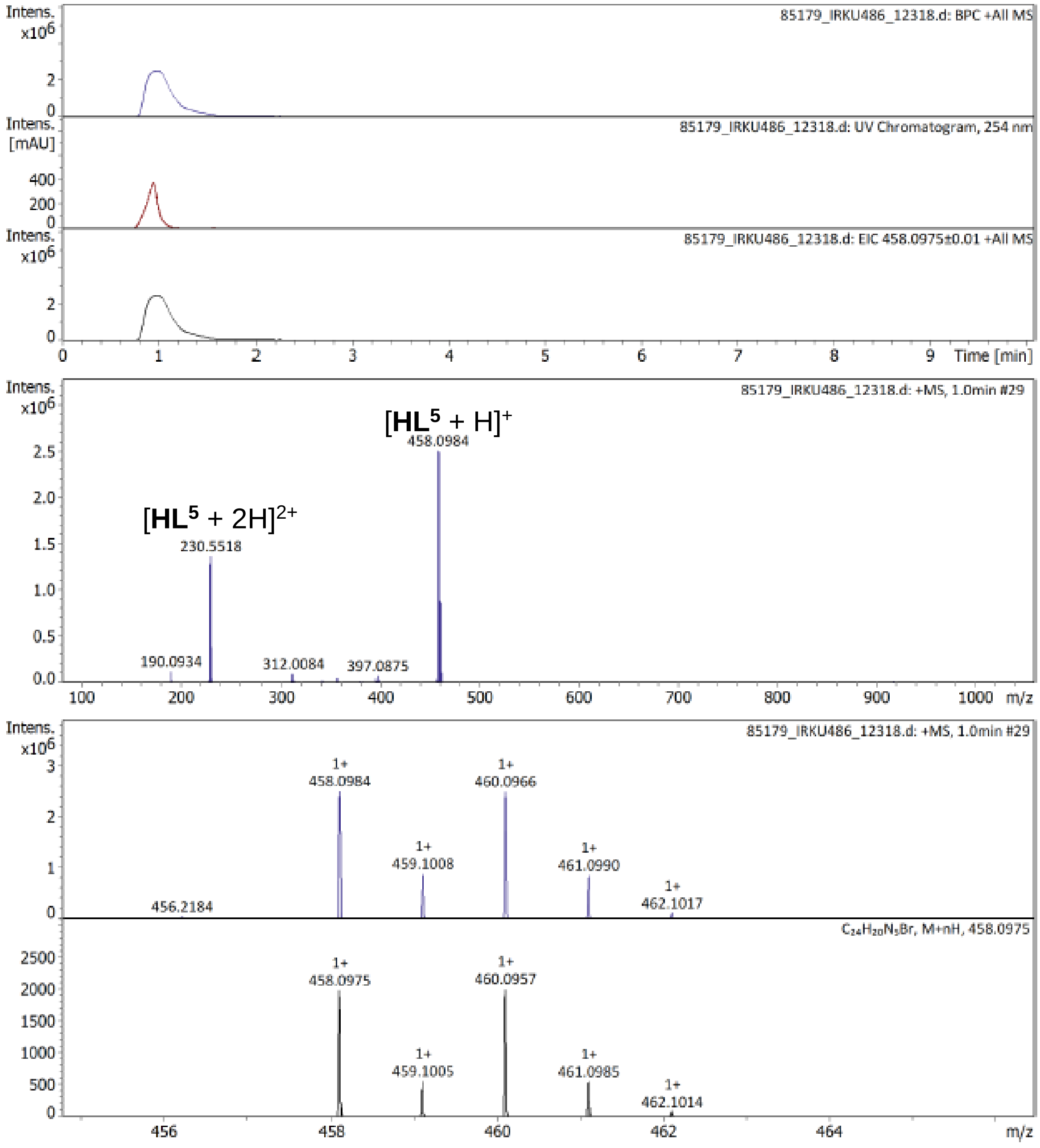

Bruker Compass DataAnalysis 5.1 printed: 24/11/2021 18:30:57 by: admin Page 1 of 1

Figure S27. Purity control of ligand **HL⁵** via HPLC and HR ESI MS.

Generic Display Report

Analysis Info		Acquisition Date	24/11/2021 18:02:35
Analysis Name	W:\MS_MessService\85178000001-85180_lc-ms_Wittmann\85180_IRKU494_12321.d		
Method	lc-ms1_r50-1900_isocrat50meoh-acn_300ulmin_10min_pos_wittmann.m	Operator	msc
Sample Name	85180_IRKU494	Instrument	maXis
Comment			

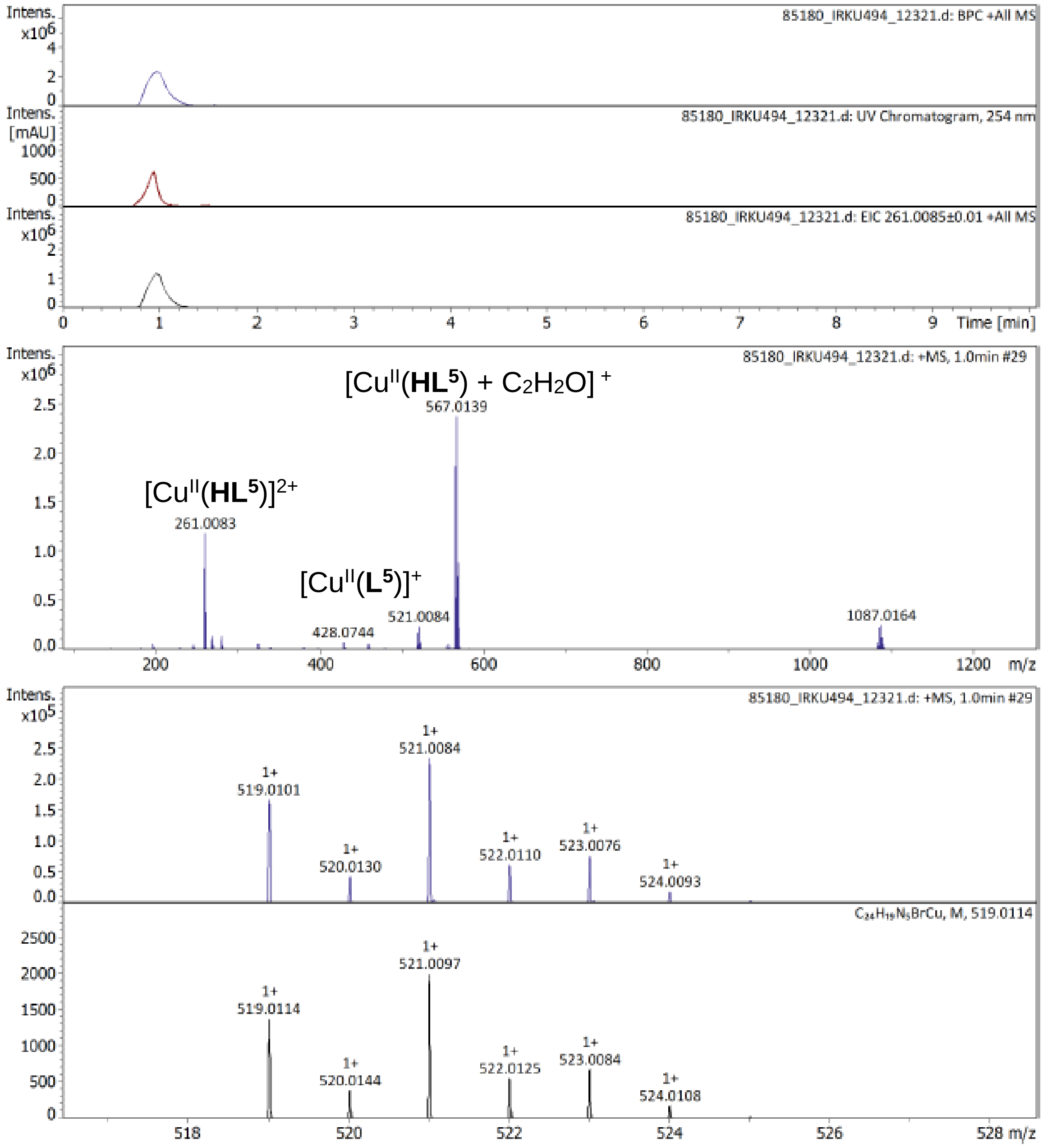

Bruker Compass DataAnalysis 5.1 printed: 24/11/2021 18:47:52 by: admin Page 1 of 1

Figure S28. Purity control of complex **5** via HPLC and HR ESI MS.

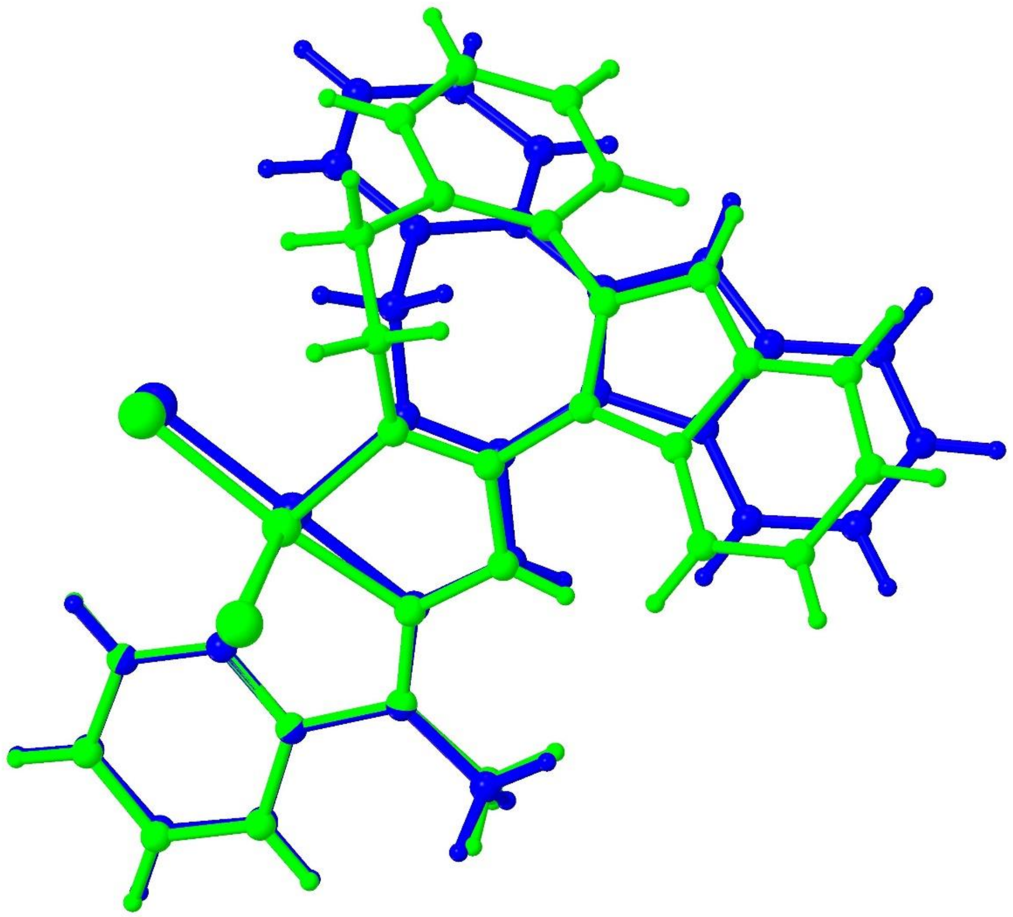

Figure S29. Overlay of the 5,6,7,9-tetrahydro-8*H*-indolo[3,2-*e*]benzazocin-8-one derived complex **3** (green trace) with a related complex based on 5,8-dihydroindolo[3,2-*d*]benzazepin-7(6*H*)-one (blue trace) reported in ref. 15.

UV-vis spectra

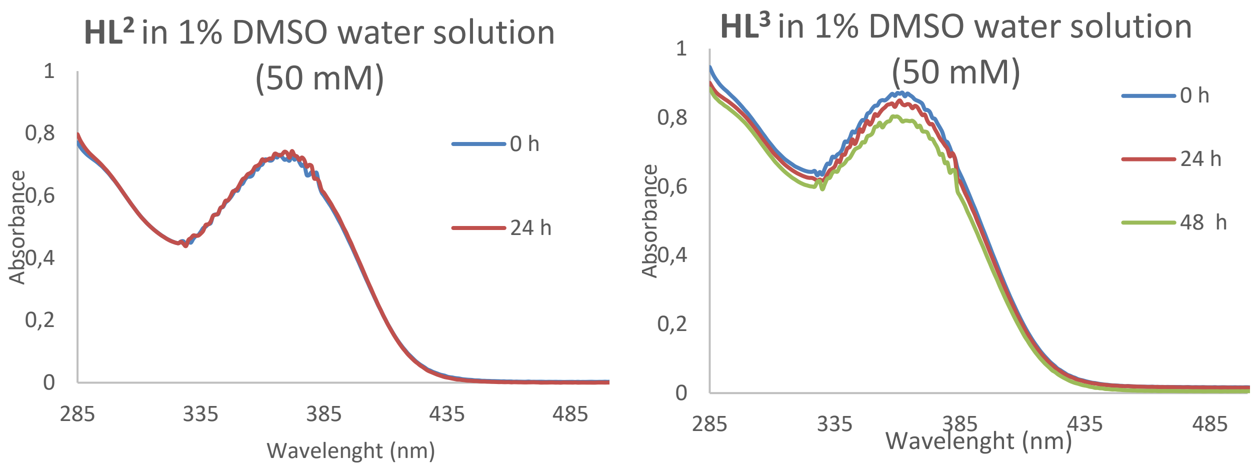

Figure S30. UV-Vis spectra for ligands **HL²** and **HL³** in aqueous solution containing 1% DMSO over 24 and 48 h.

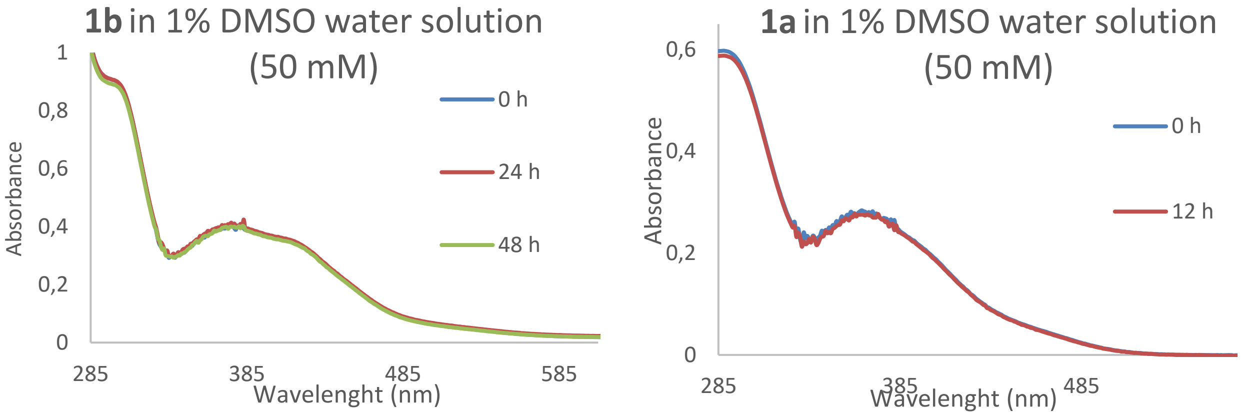

Figure S31. UV-Vis spectra for complexes **1a** and **1b** in aqueous solution containing 1% DMSO over 12 and 48 h.

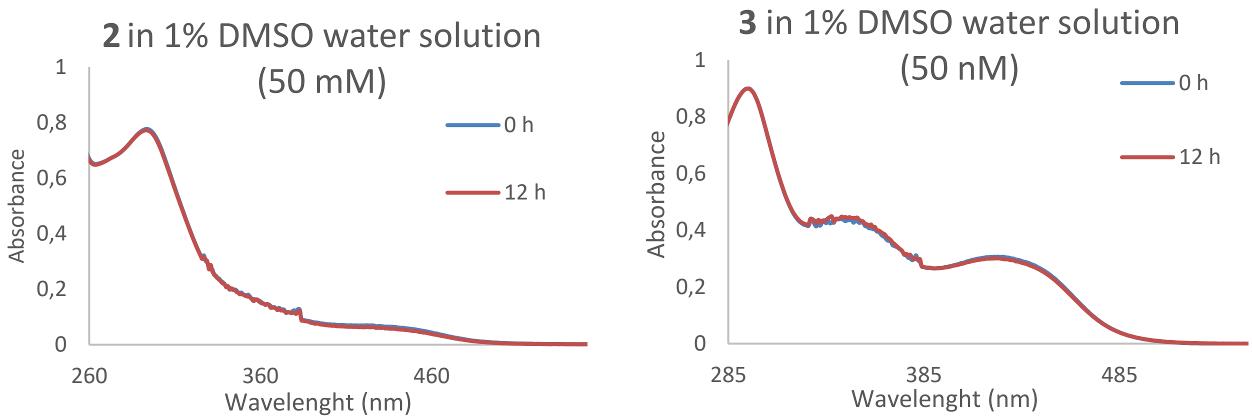

Figure S32. UV-Vis spectra for complexes **2** and **3** in aqueous solution containing 1% DMSO over 12 h.

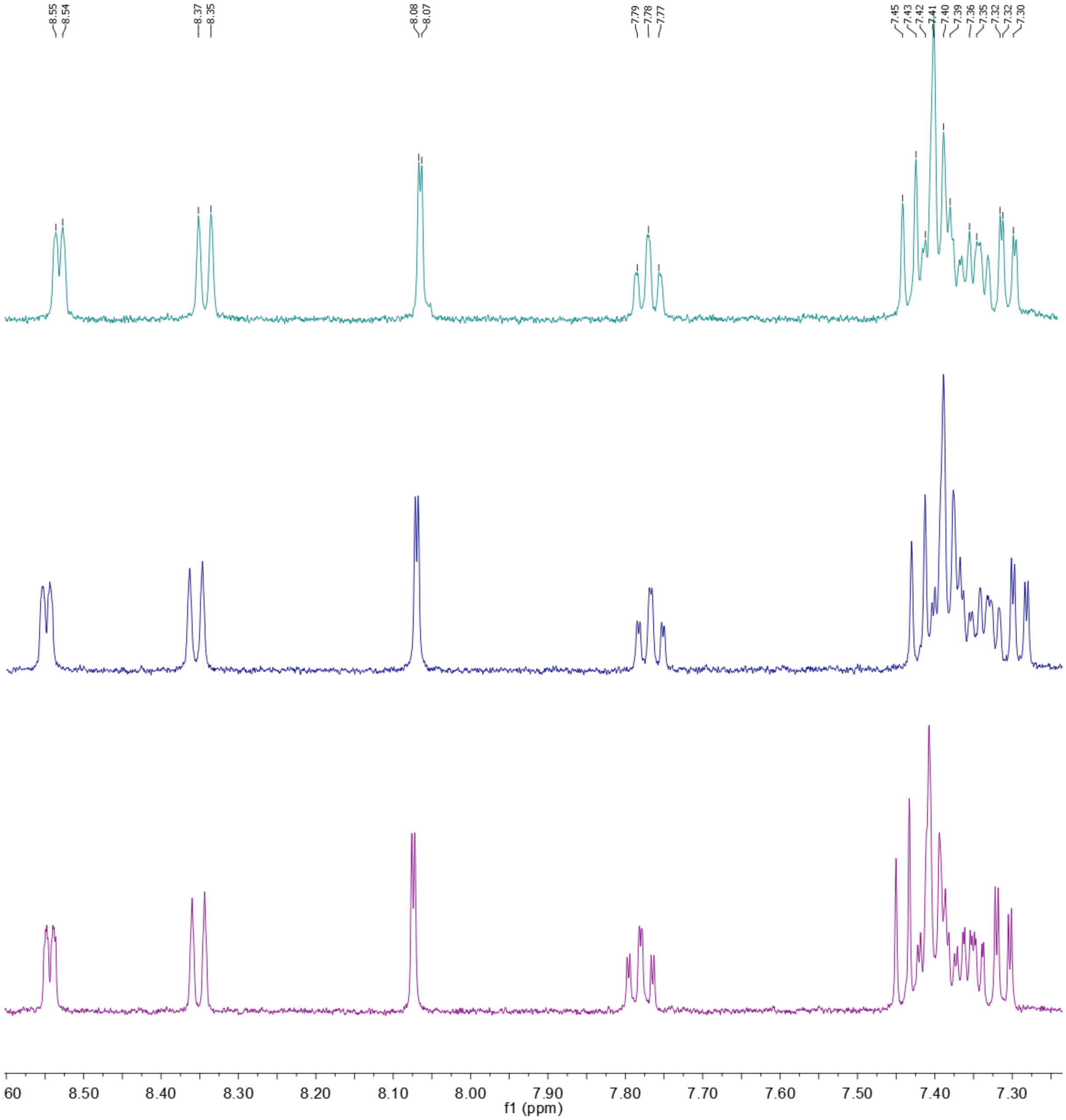

Figure S33. ^{1}H NMR spectra of **HL^5** in DMSO-d_6/D_2O 2:1 measured vs time. From top to bottom: green: t = 0 h; blue t = 2 h; lilac: t = 20 h.

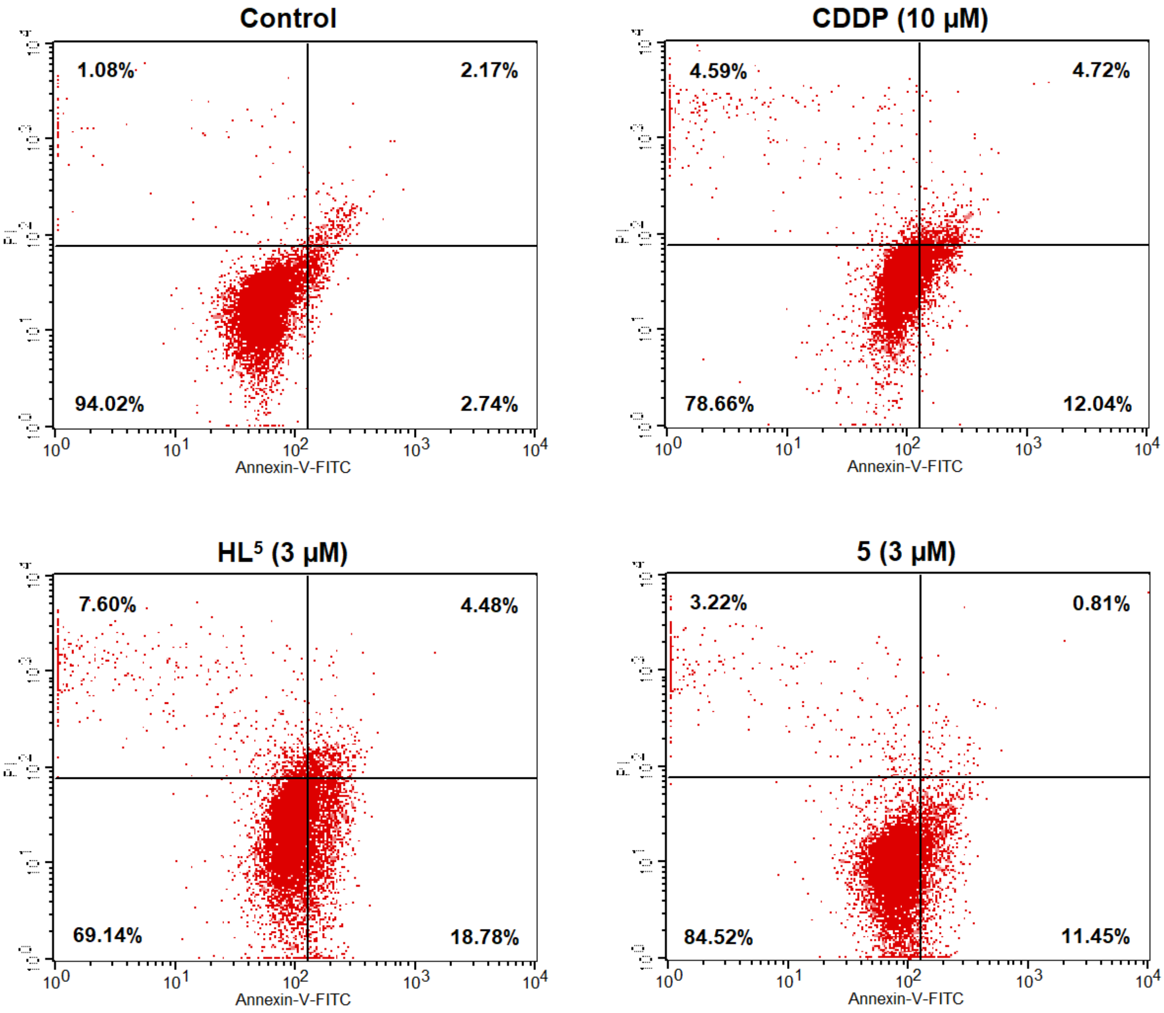

Figure S34. Representative dot plot diagrams obtained by flow-cytometric analysis of treated HCT 116 cells after dual staining with Annexin-V-FITC and PI. Annexin-V-FITC/PI staining was monitored in HCT 116 cells, following 48 h treatment with CDDP, ligand HL5 and complex **5**. Intact cells can be seen at lower-left quadrant, early apoptotic cells at lower-right quadrant, late apoptotic or necrotic cells at upper-right quadrant, and necrotic cells at upper-left quadrant.

Enzymes inhibition

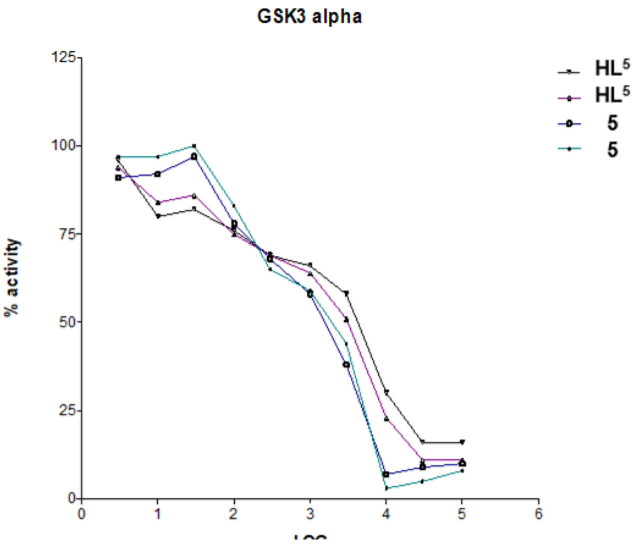

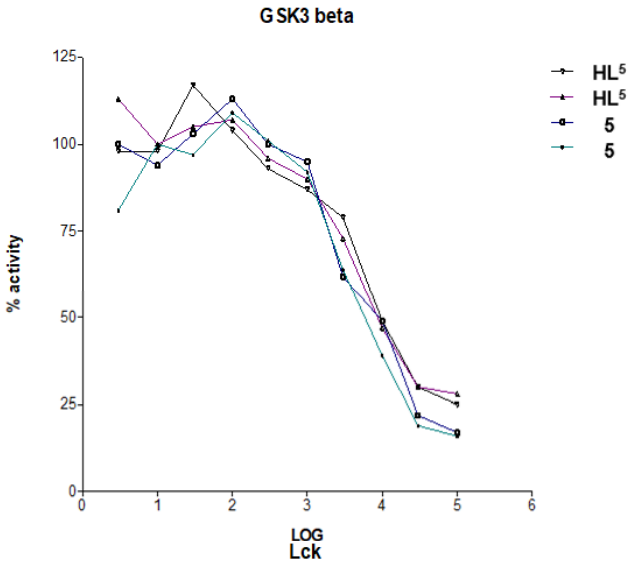

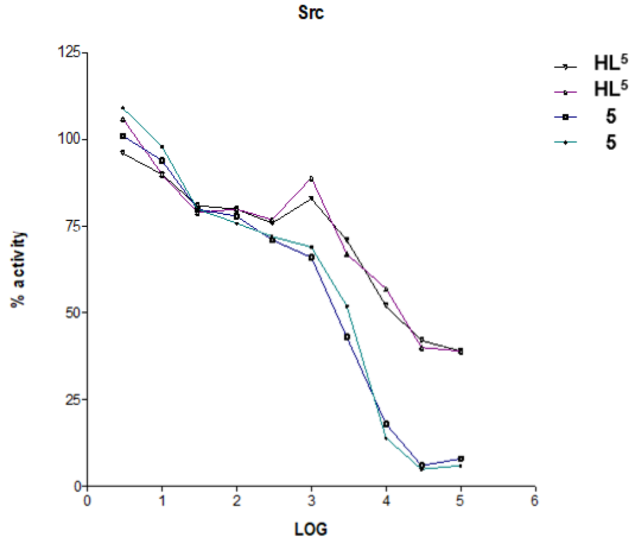

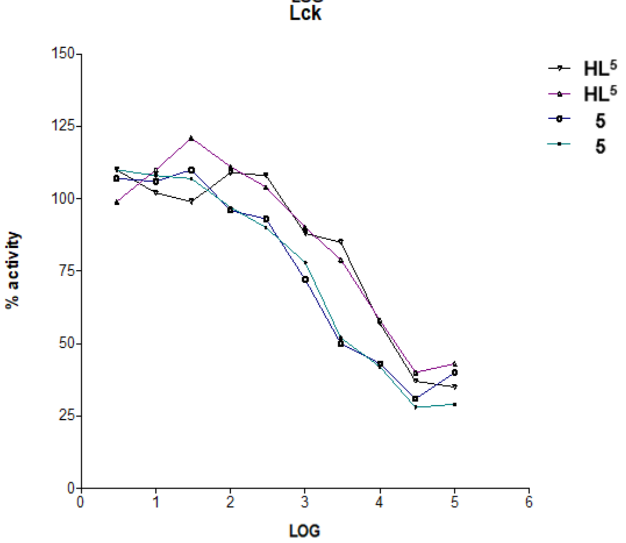

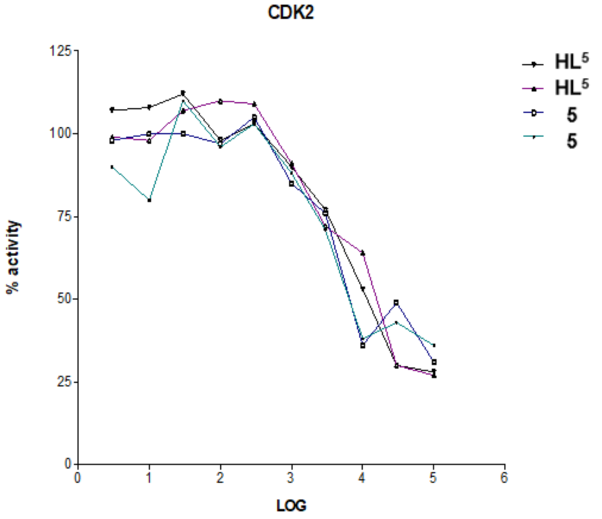

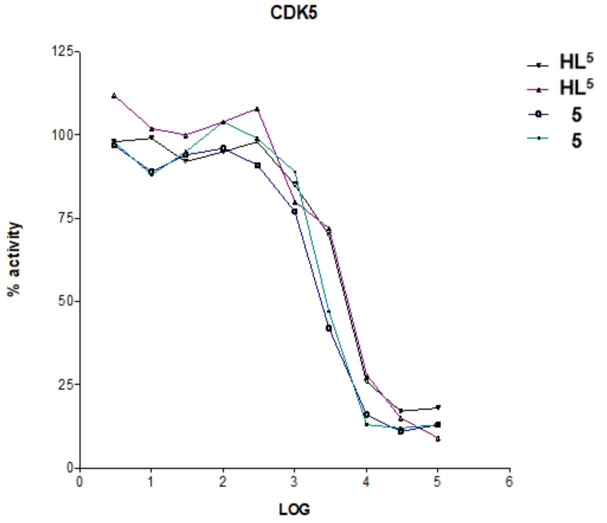

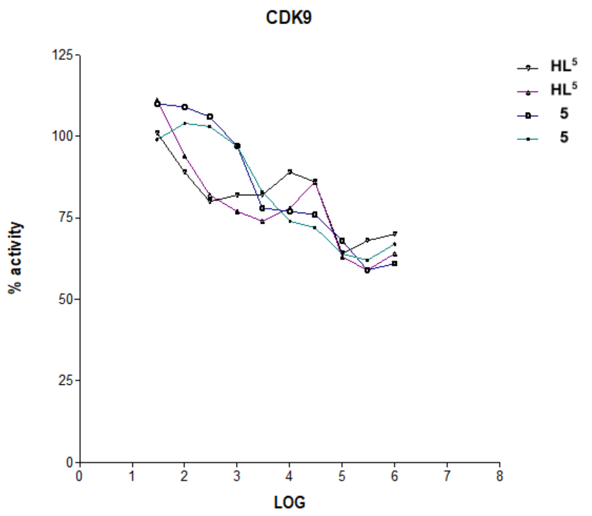

Figure S35. Activity of the **HL⁵** and **5** against the protein kinases GSK-3α, GSK-3β, Src, Lck, CDK2, CDK5 and CDK9.

NMR spectra of intermediate species

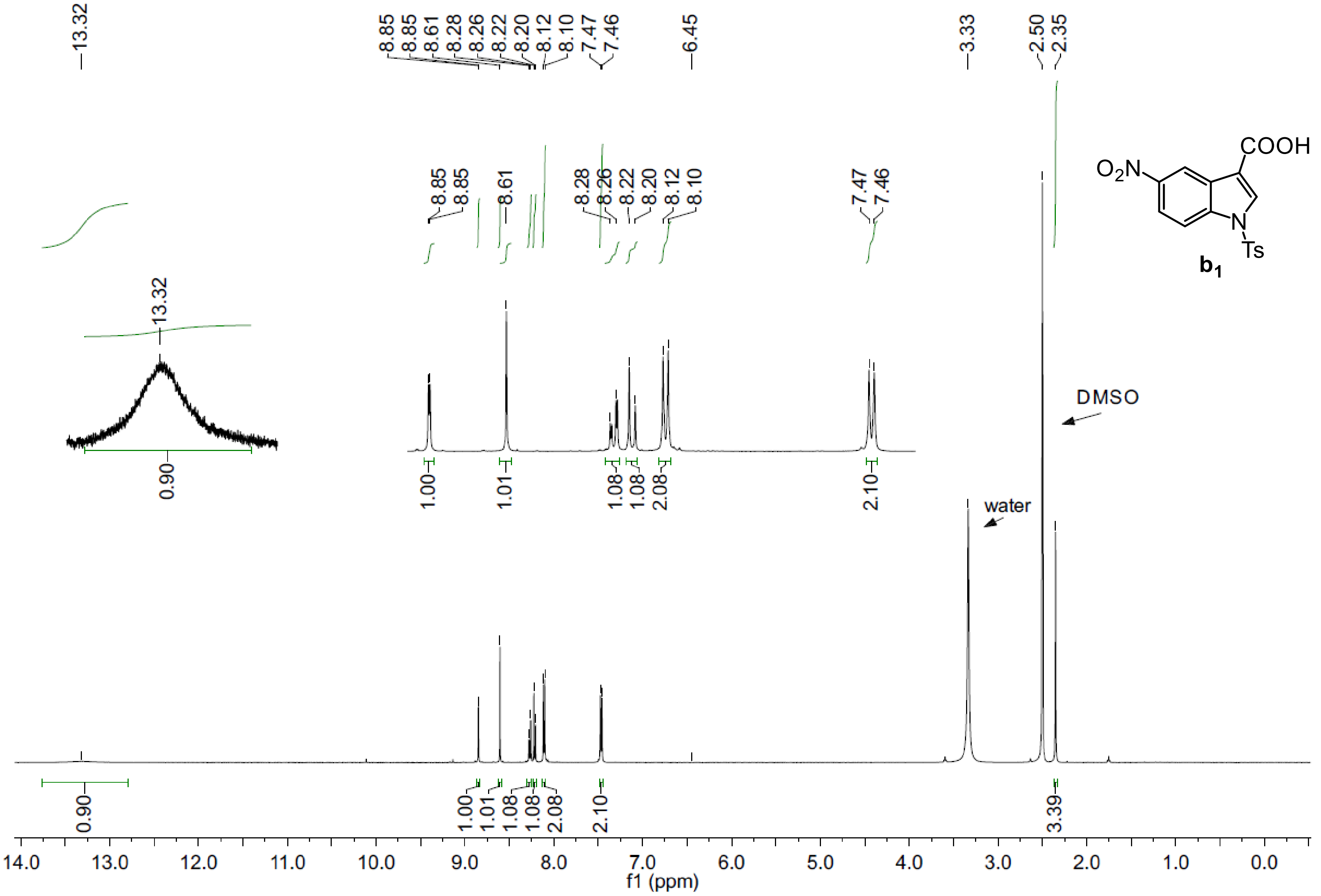

Figure S36. ^{1}H NMR spectrum of **b$_1$** in DMSO (700 MHz).

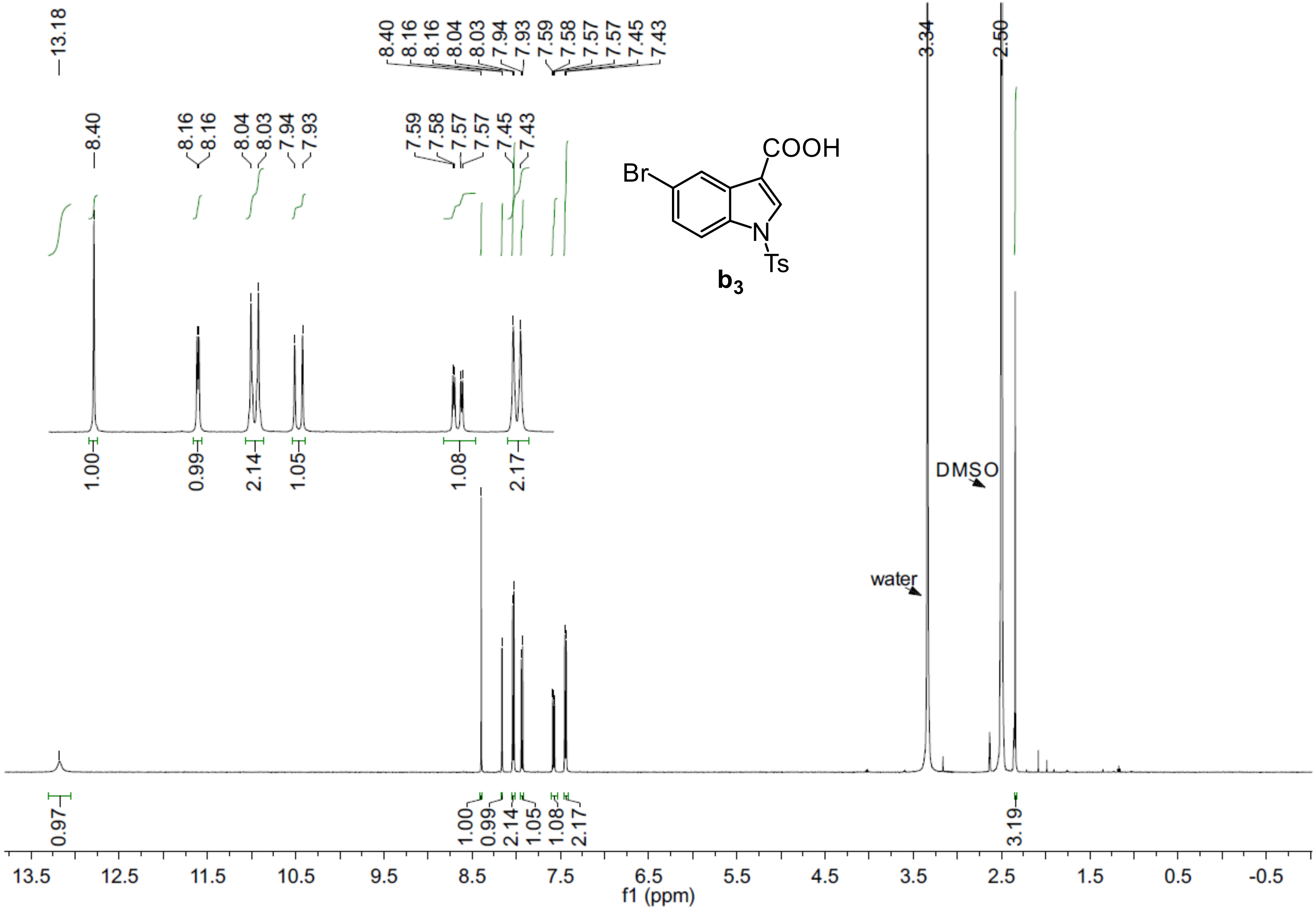

Figure S37. ^{1}H NMR spectrum of **b₂** in DMSO (500 MHz).

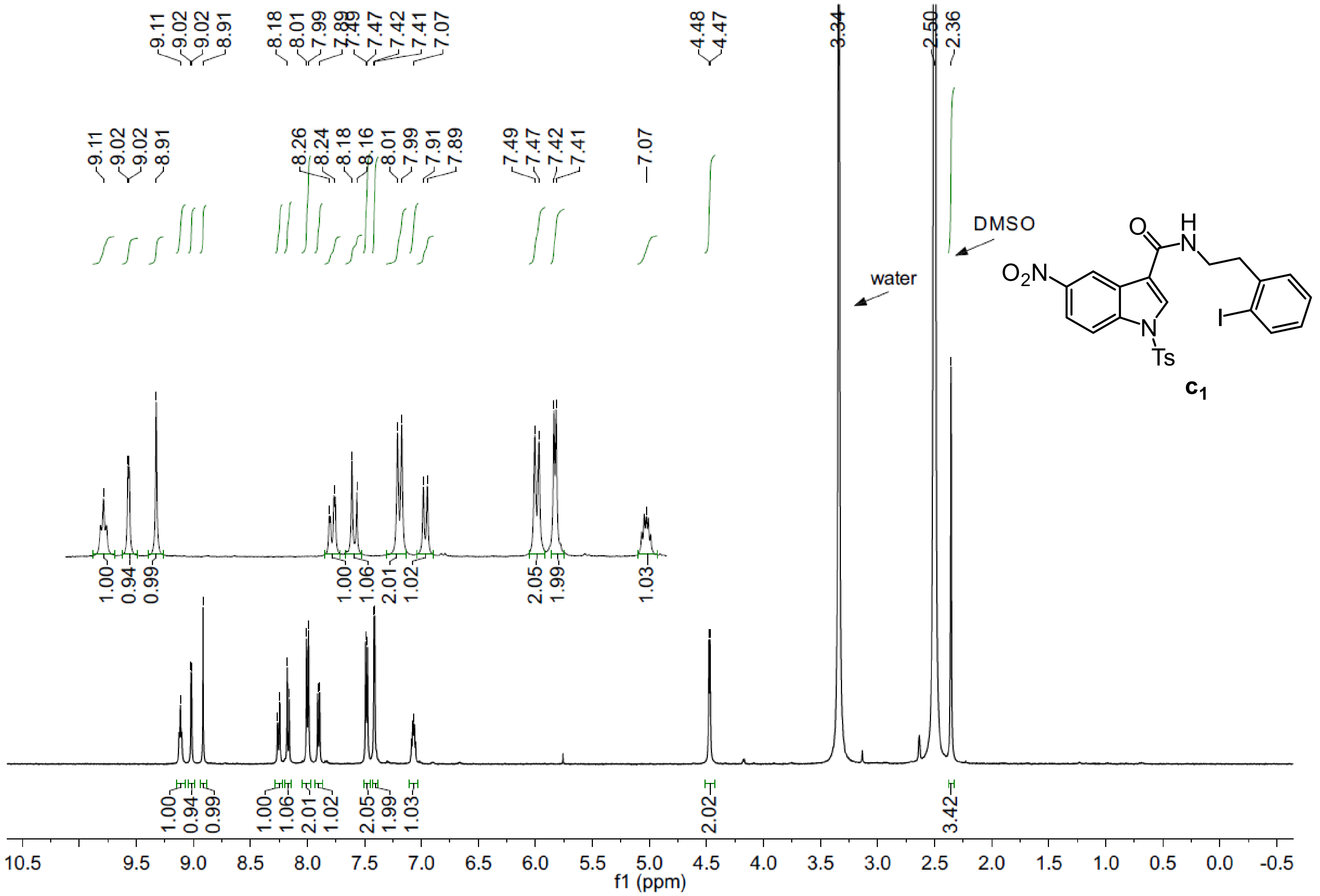

Figure S38. ^{1}H NMR spectrum of **c_1** in DMSO (500 MHz).

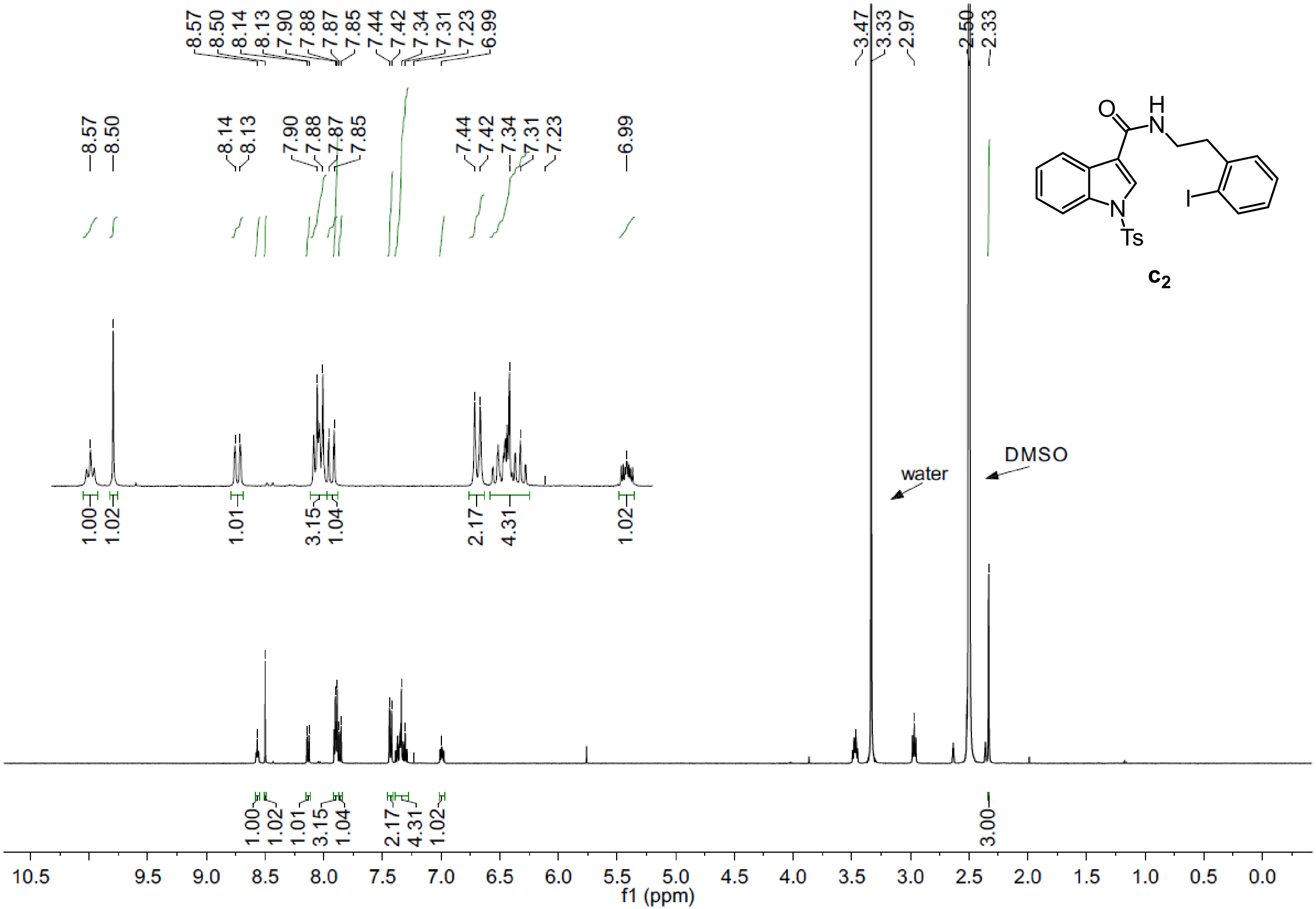

Figure S39. ^{1}H NMR spectrum of **c₂** in DMSO (500 MHz).

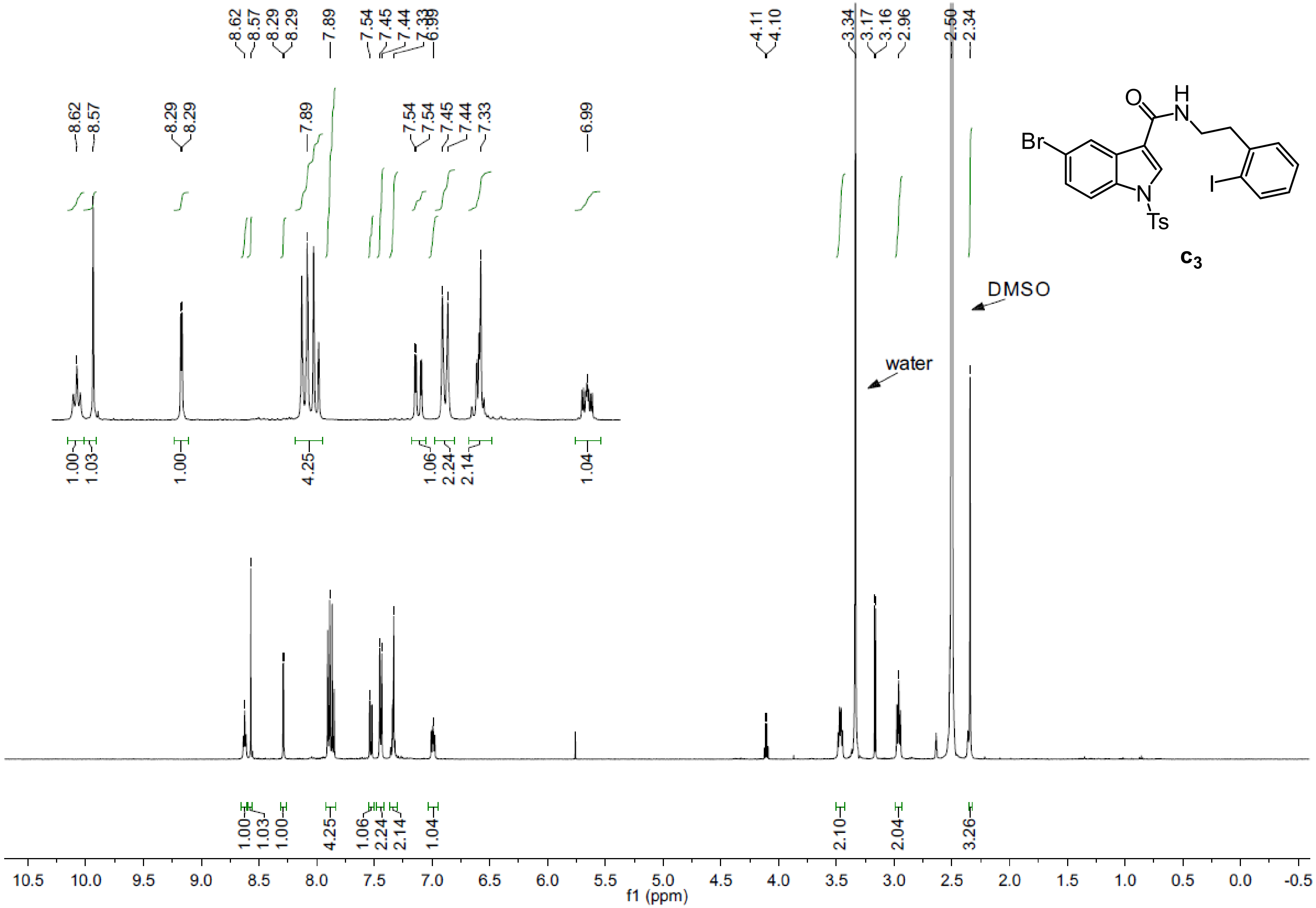

Figure S40. ^{1}H NMR spectrum of **c₃** in DMSO (500 MHz).

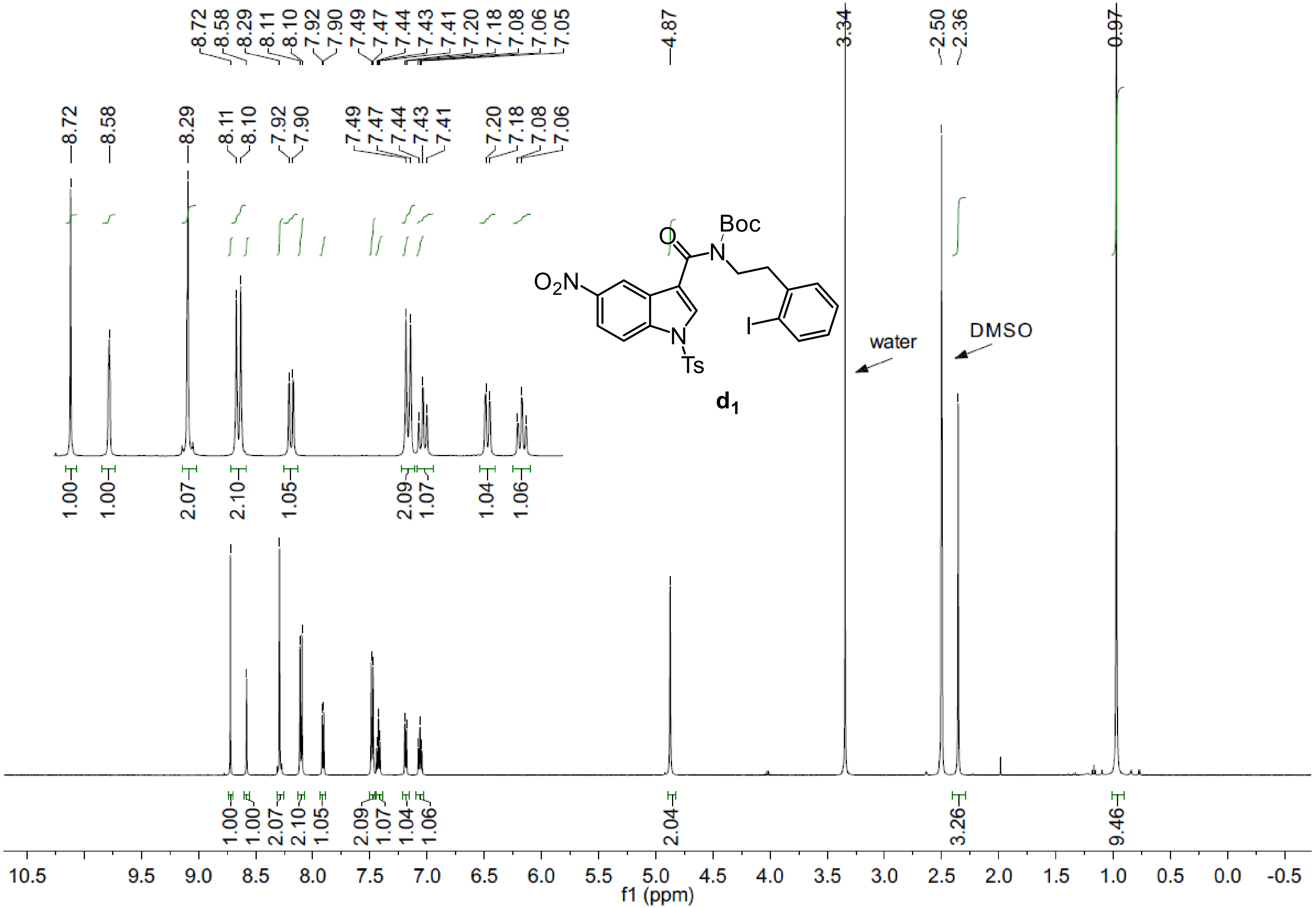

Figure S41. ^{1}H NMR spectrum of **d_1** in DMSO (500 MHz).

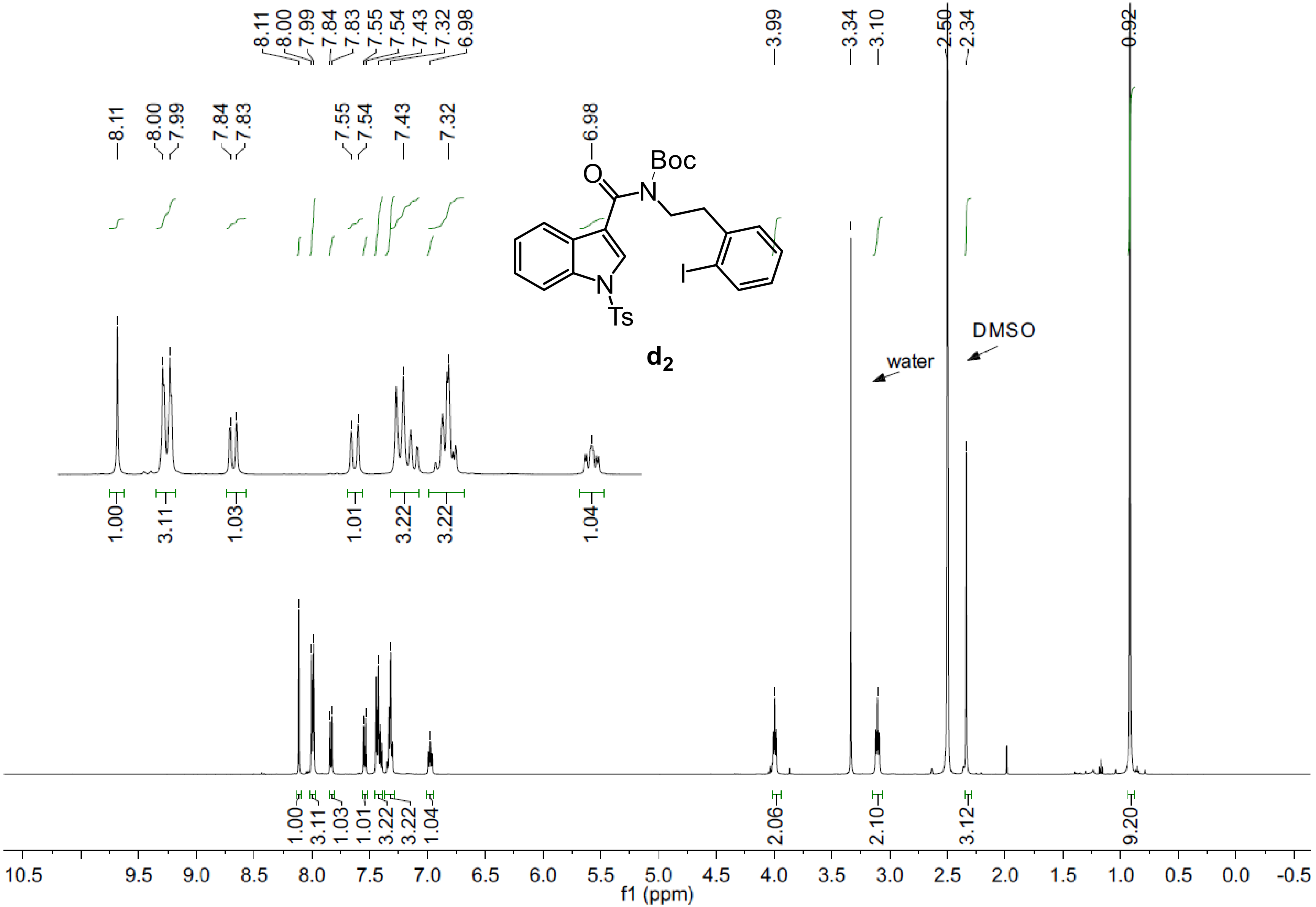

Figure S42. ^{1}H NMR spectrum of **d₂** in DMSO (500 MHz).

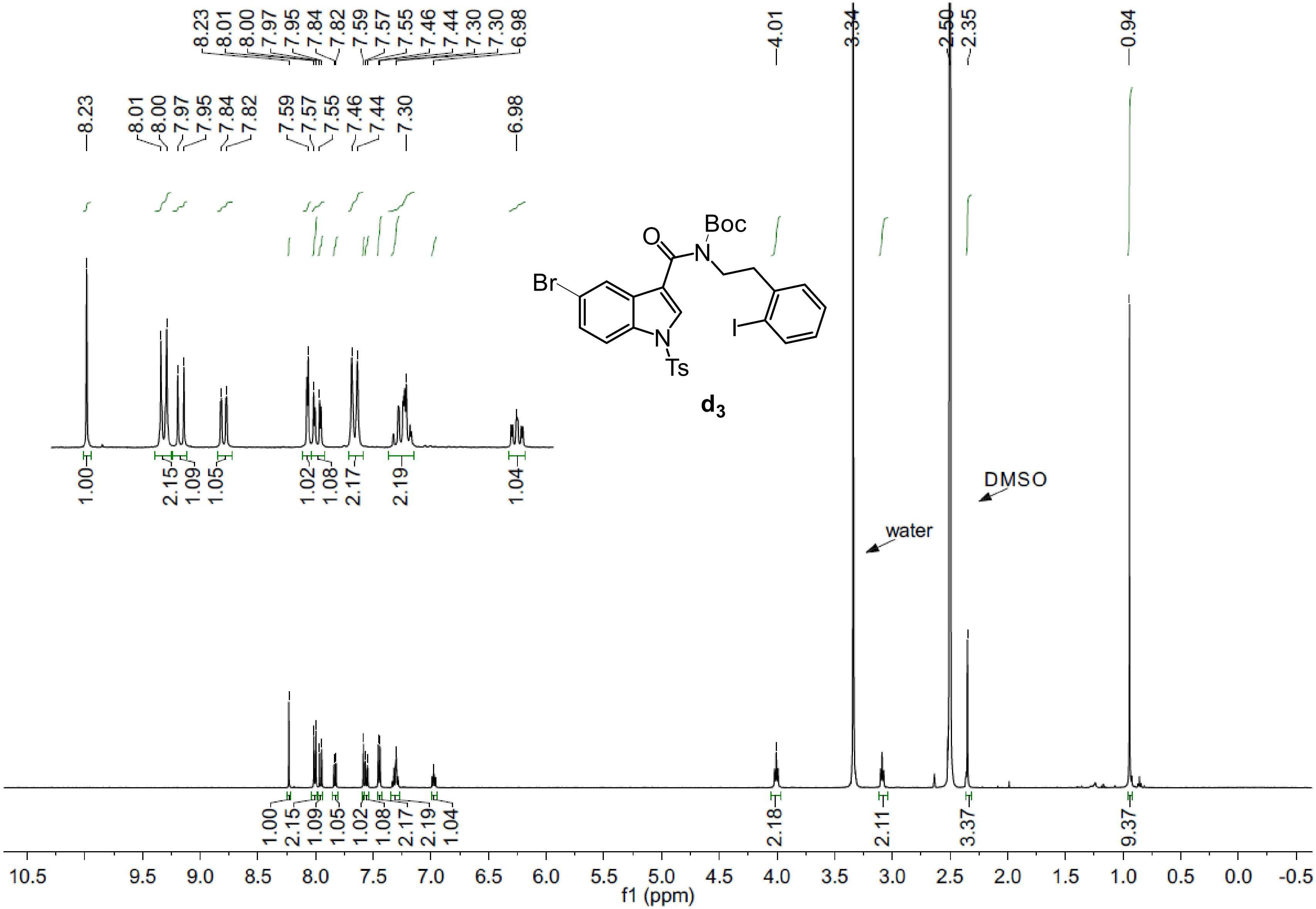

Figure S43. ^{1}H NMR spectrum of **d₃** in DMSO (500 MHz).

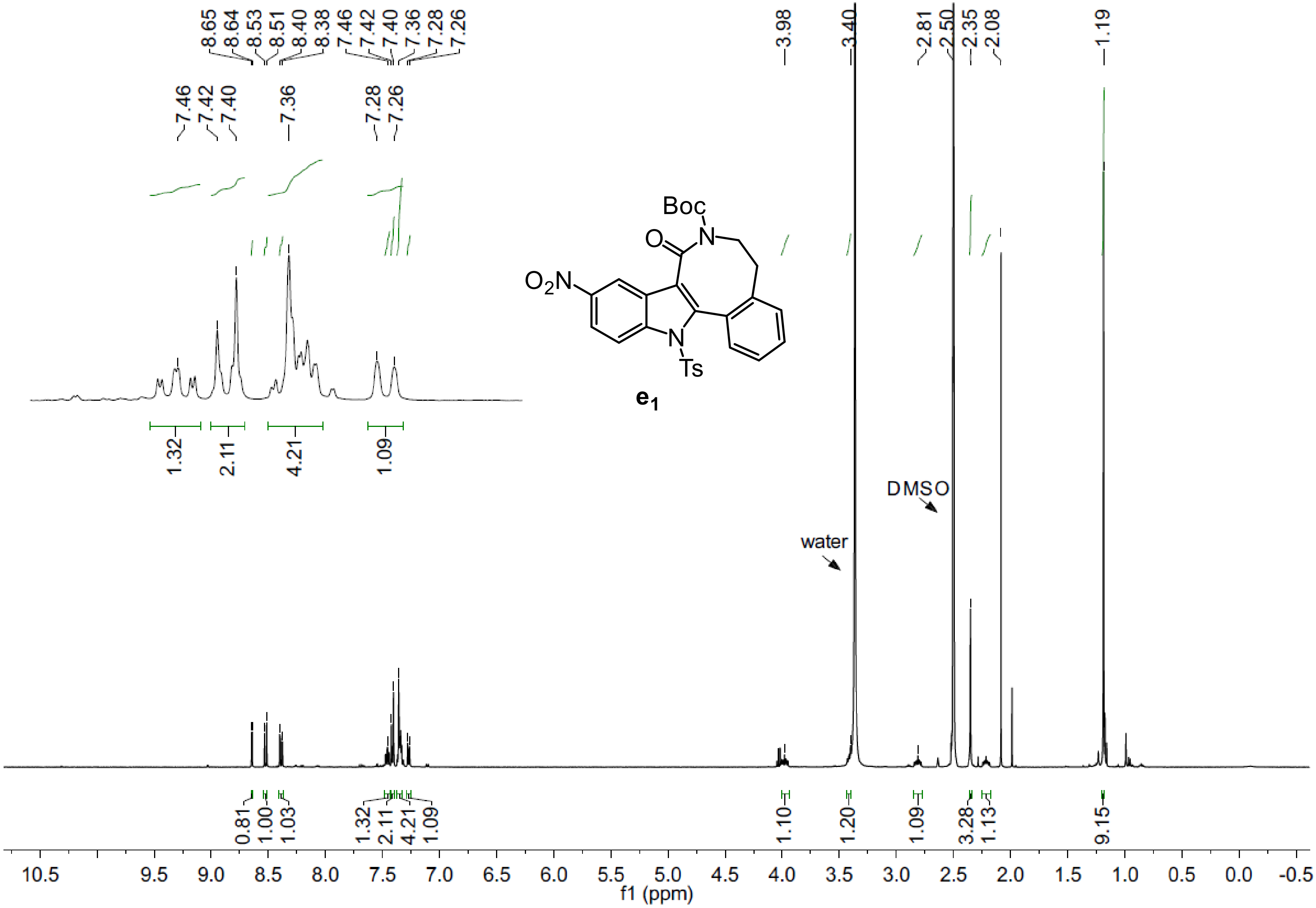

Figure S44. ^{1}H NMR spectrum of $\mathbf{e_1}$ in DMSO (500 MHz).

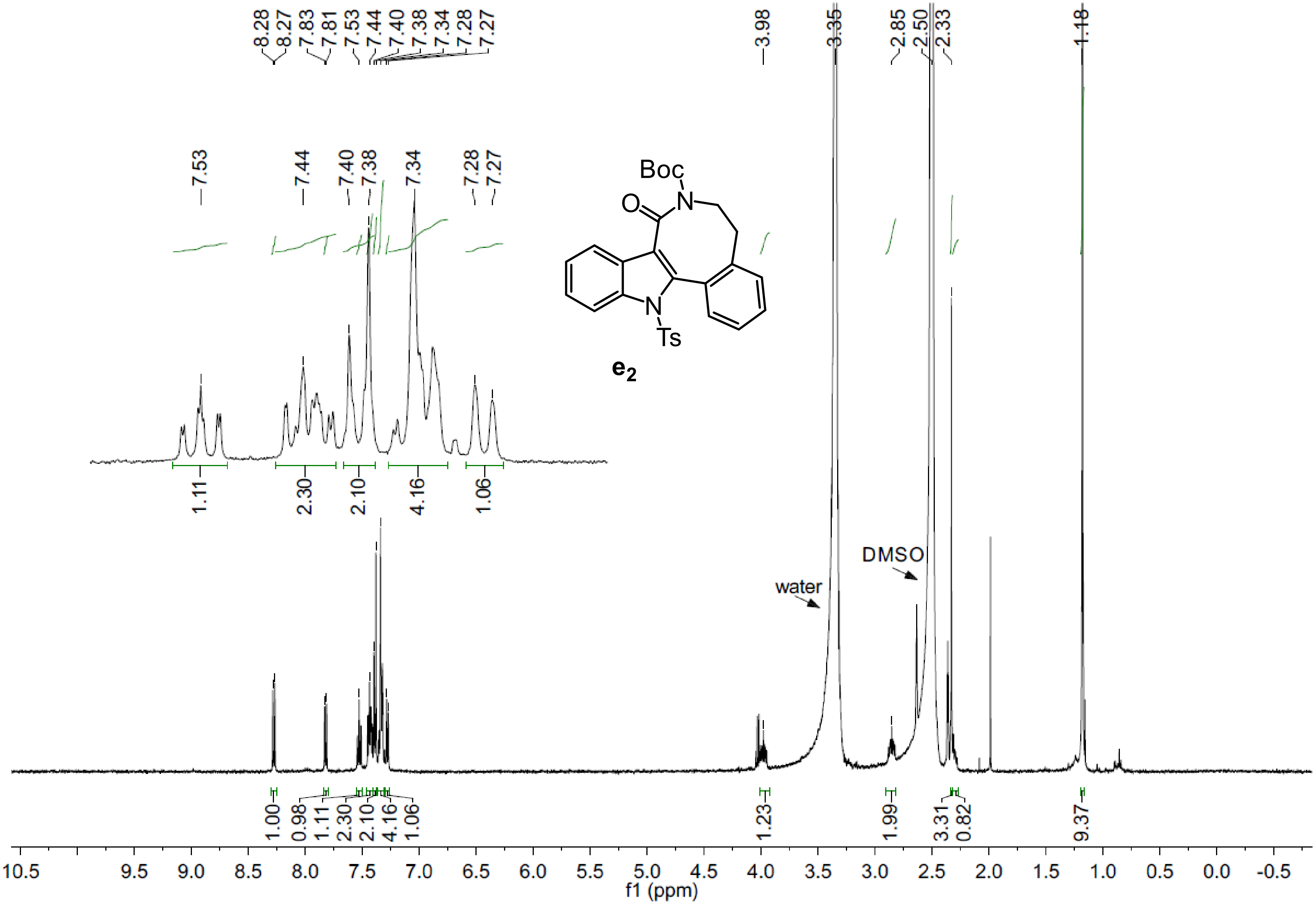

Figure S45. ^{1}H NMR spectrum of **e_2** in DMSO (500 MHz).

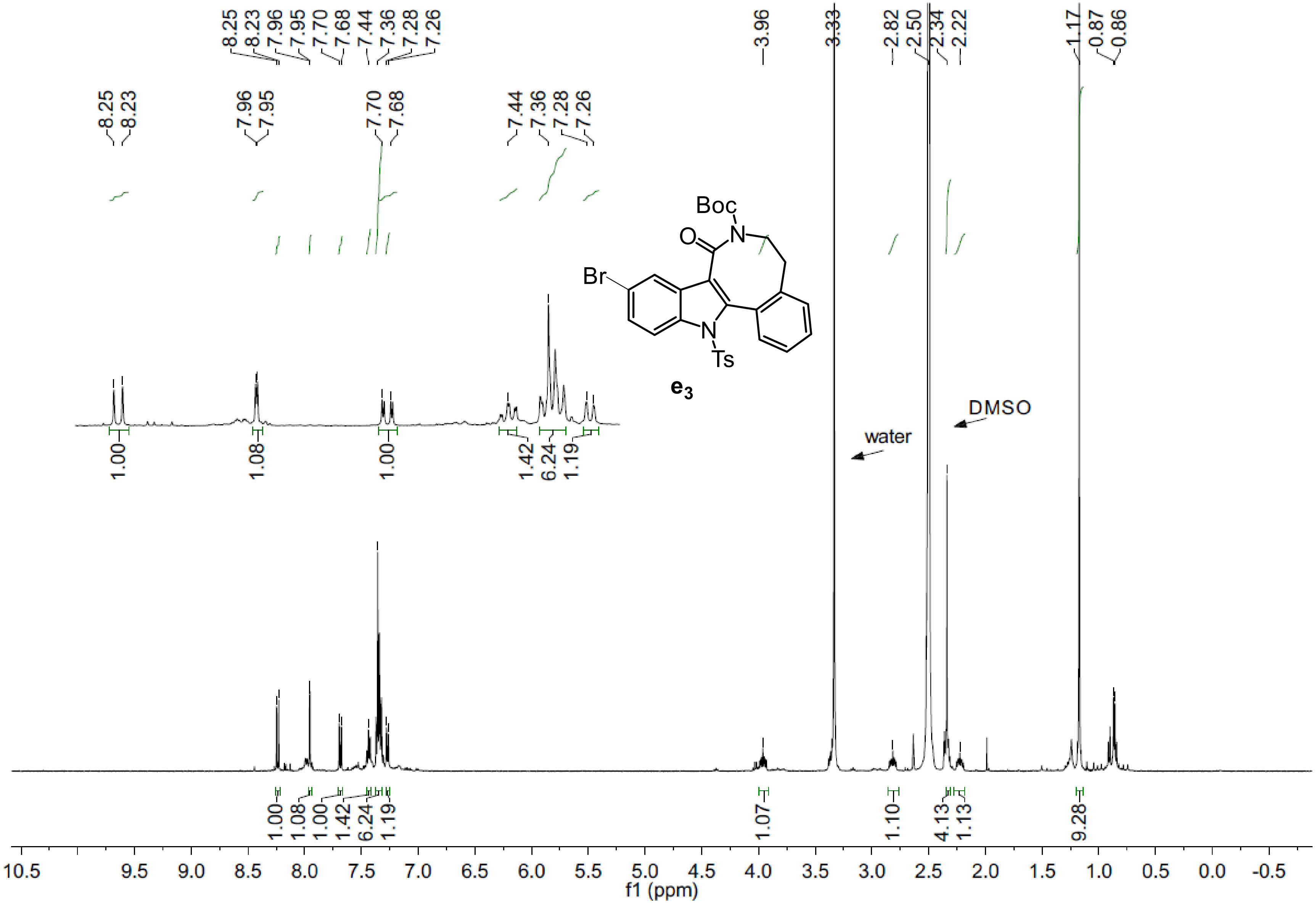

Figure S46. ^{1}H NMR spectrum of **e₃** in DMSO (500 MHz).

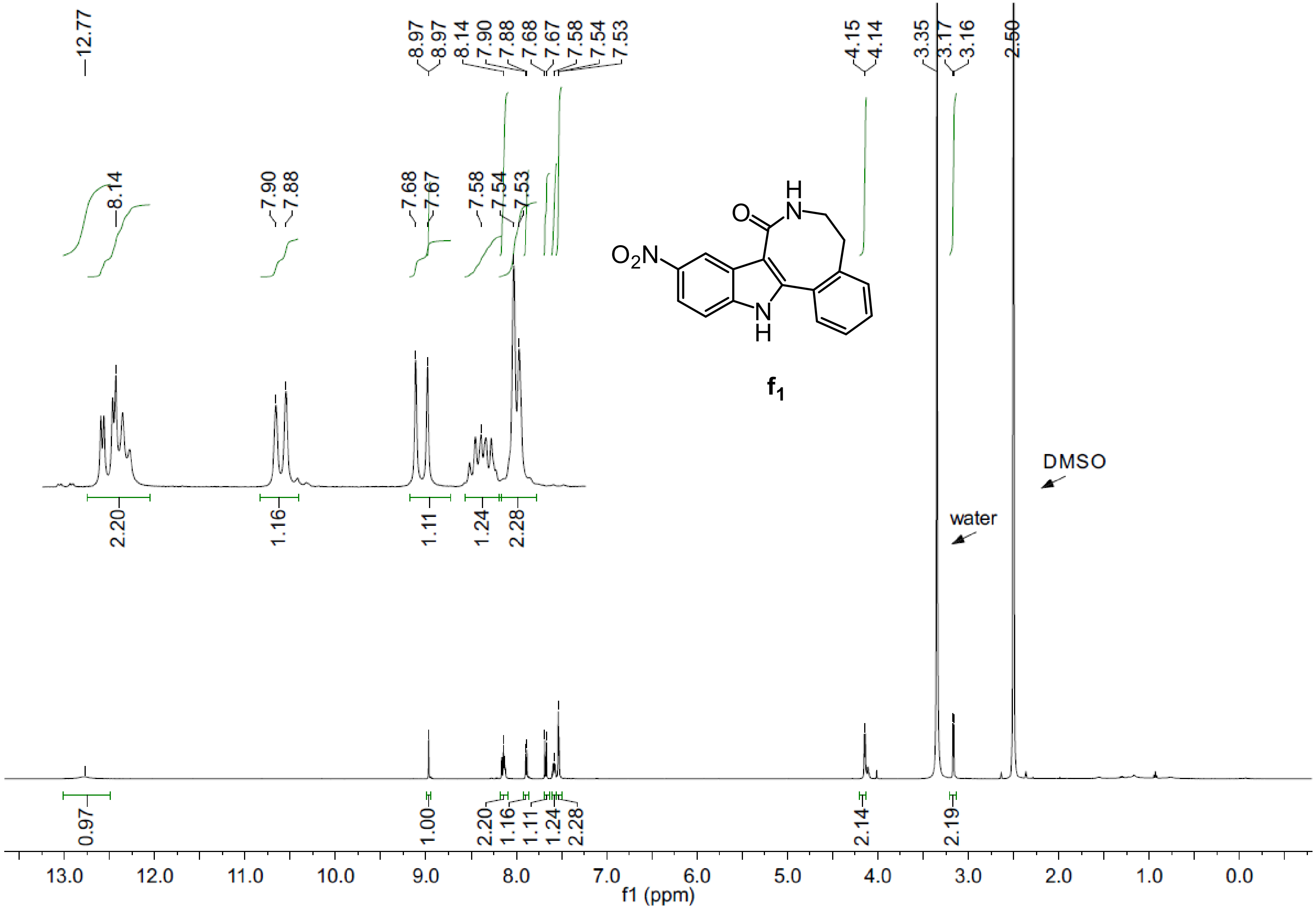

Figure S47. ^{1}H NMR spectrum of **f$_1$** in DMSO (500 MHz).

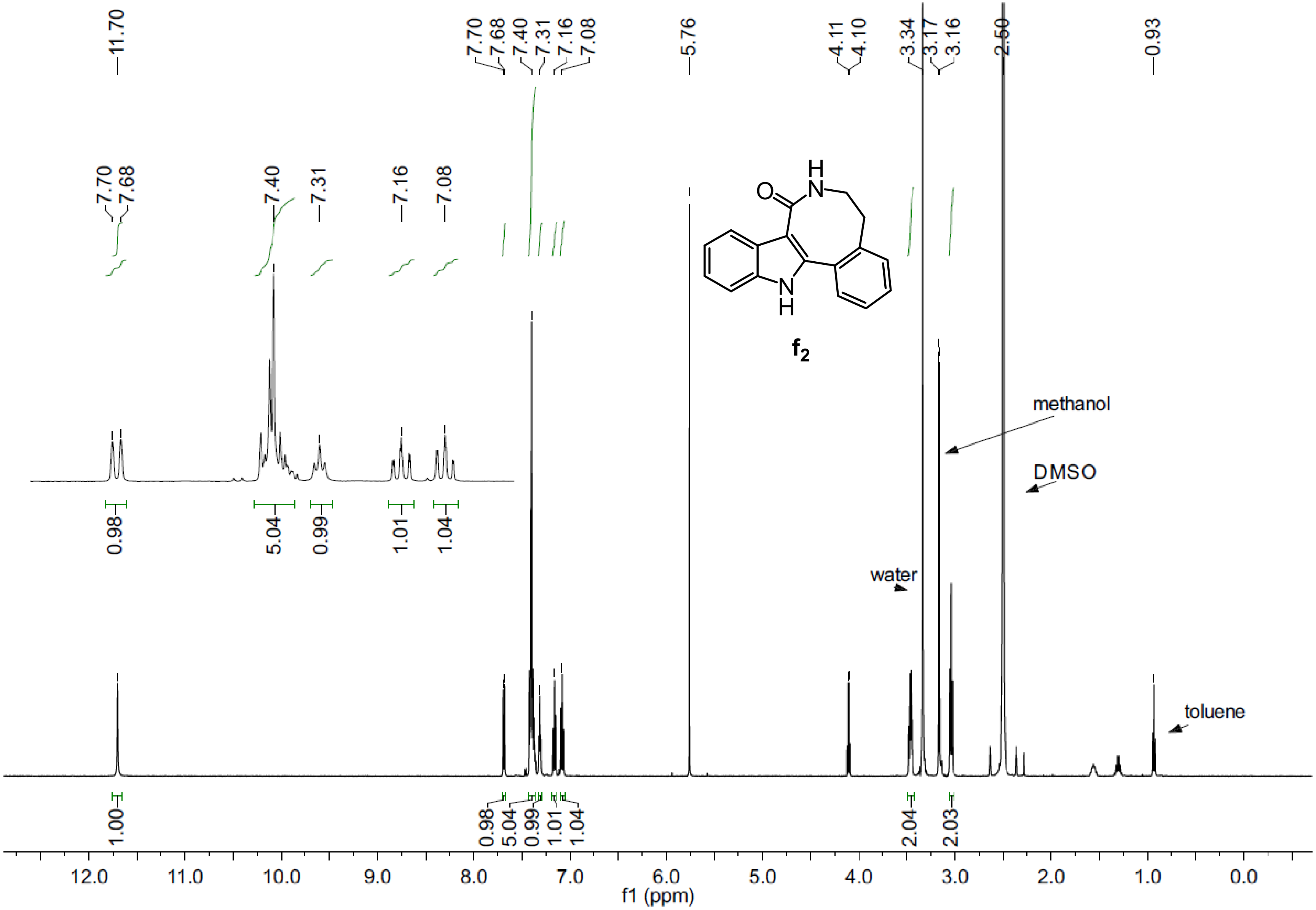

Figure S48. ¹H NMR spectrum of **f₂** in DMSO (500 MHz).

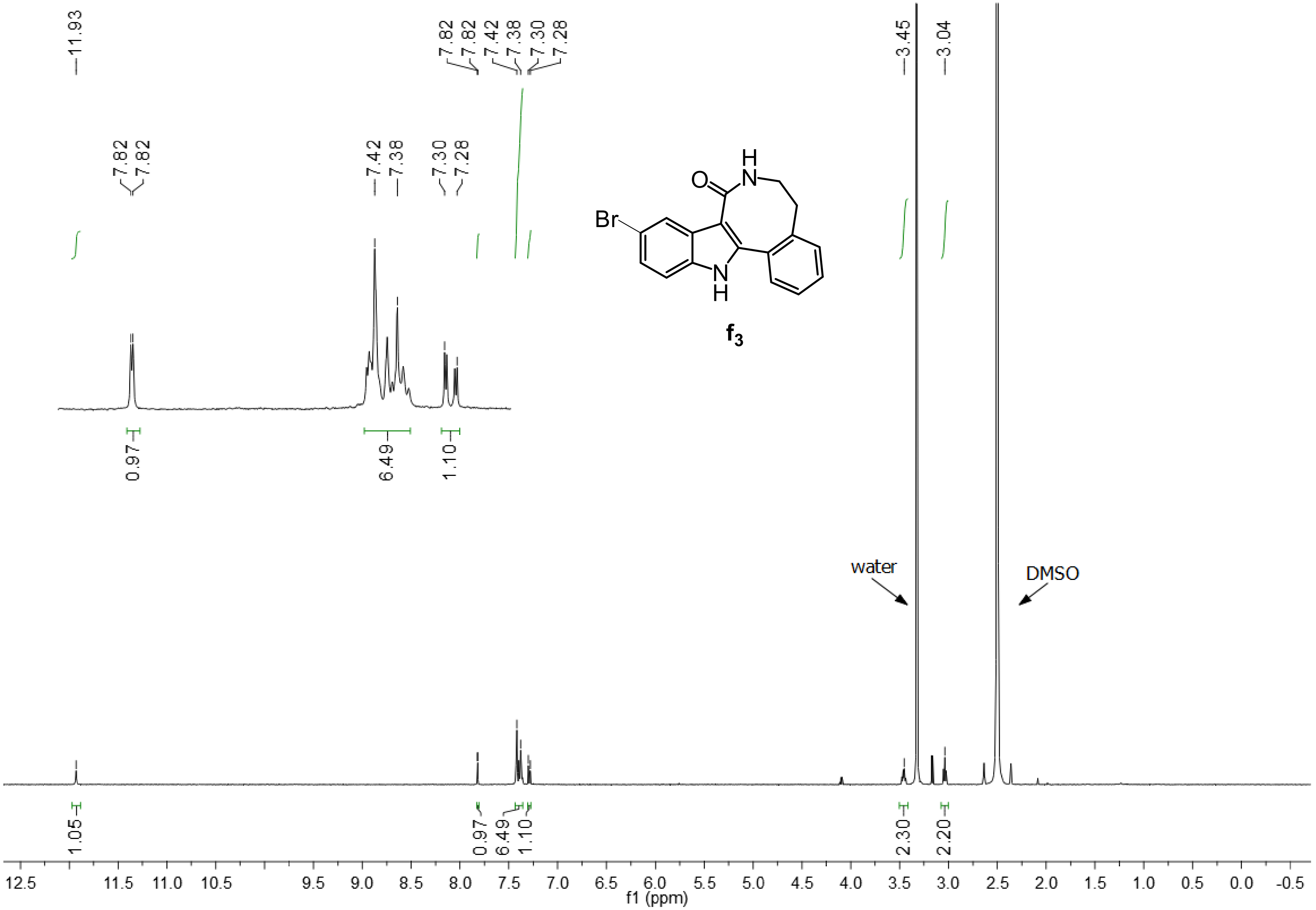

Figure S49. ^{1}H NMR spectrum of **f₃** in DMSO (500 MHz).

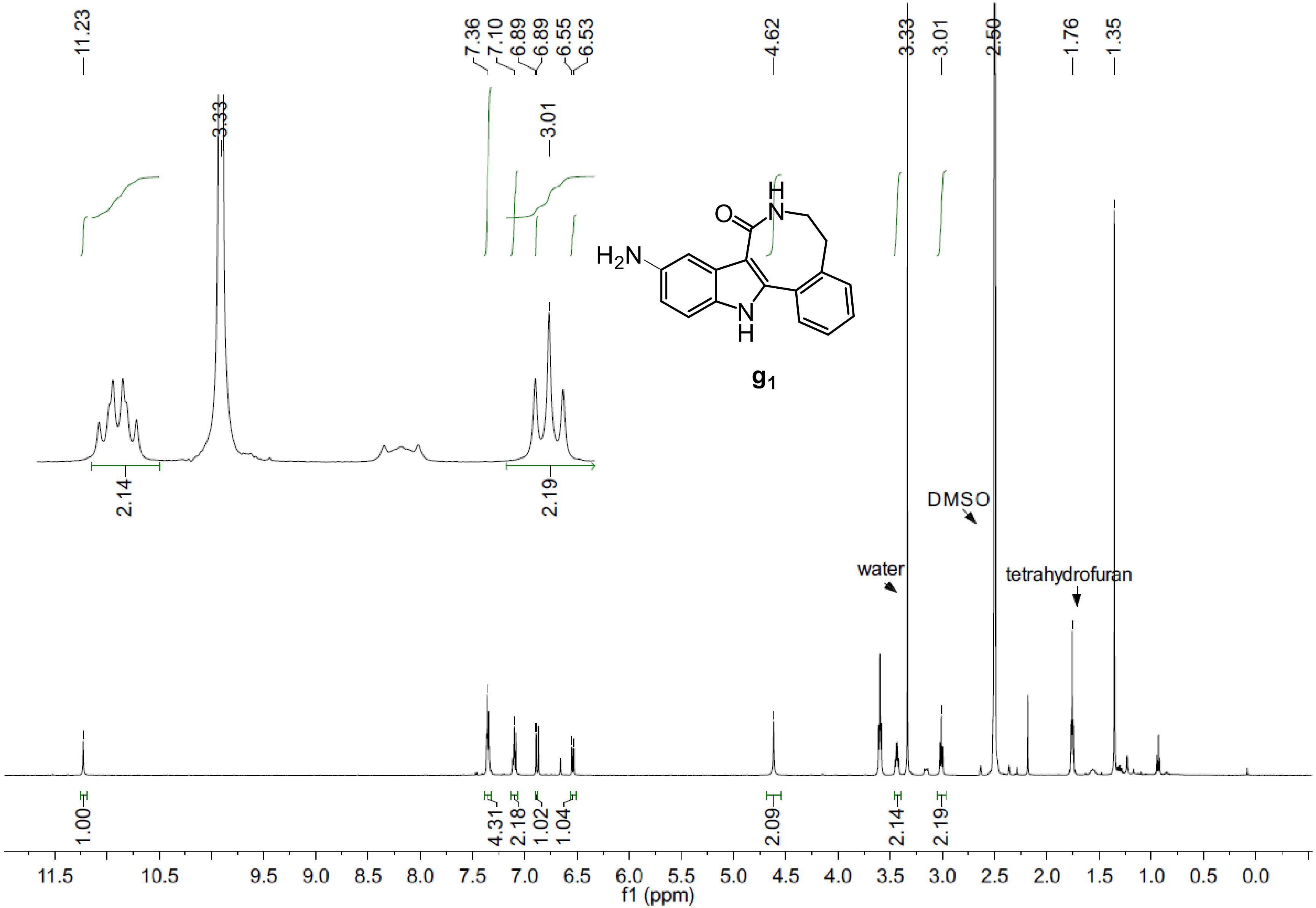

Figure S50. ^{1}H NMR spectrum of **g_1** in DMSO (500 MHz).

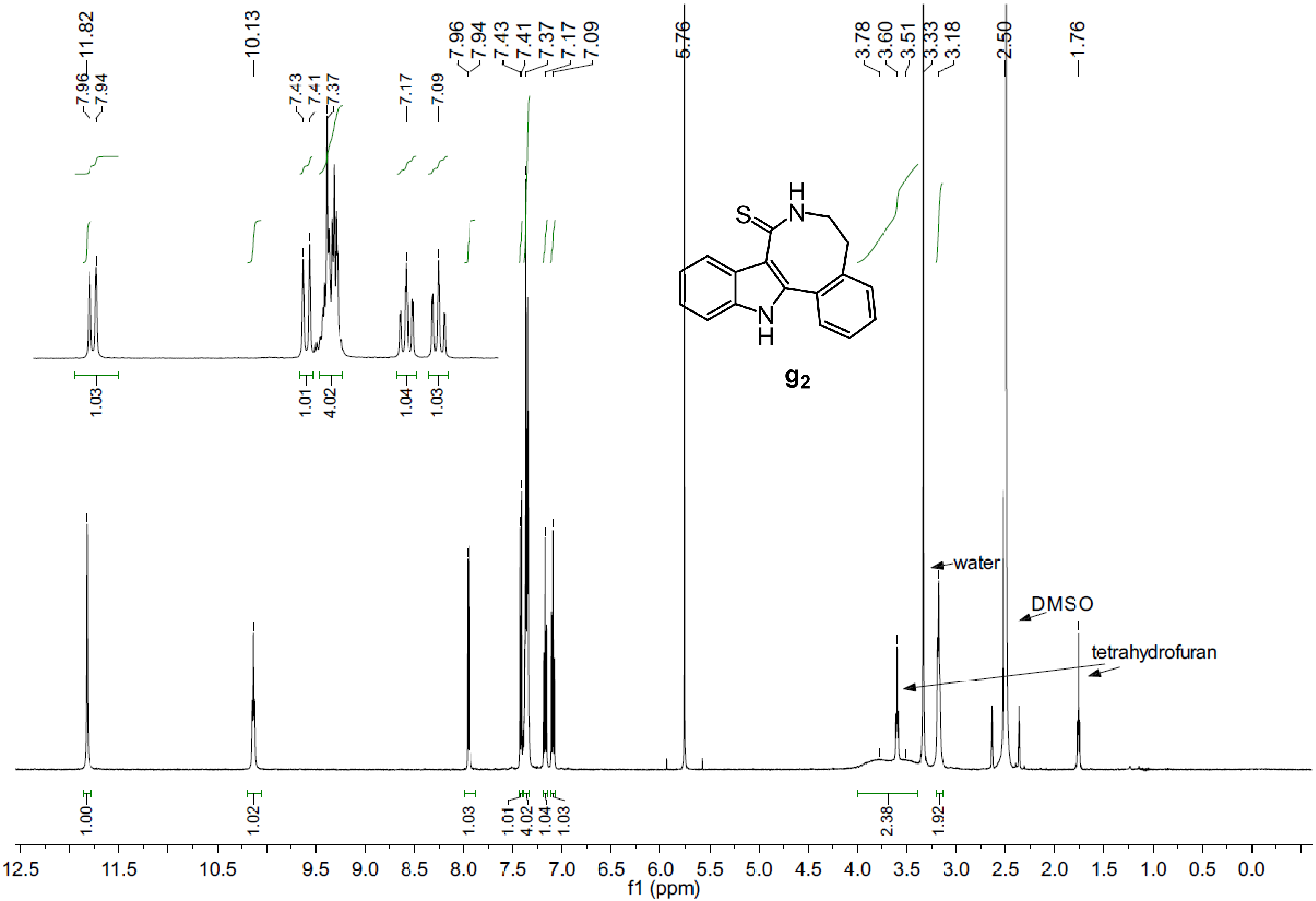

Figure 51 ^{1}H NMR spectrum of **g₂** in DMSO (500 MHz).

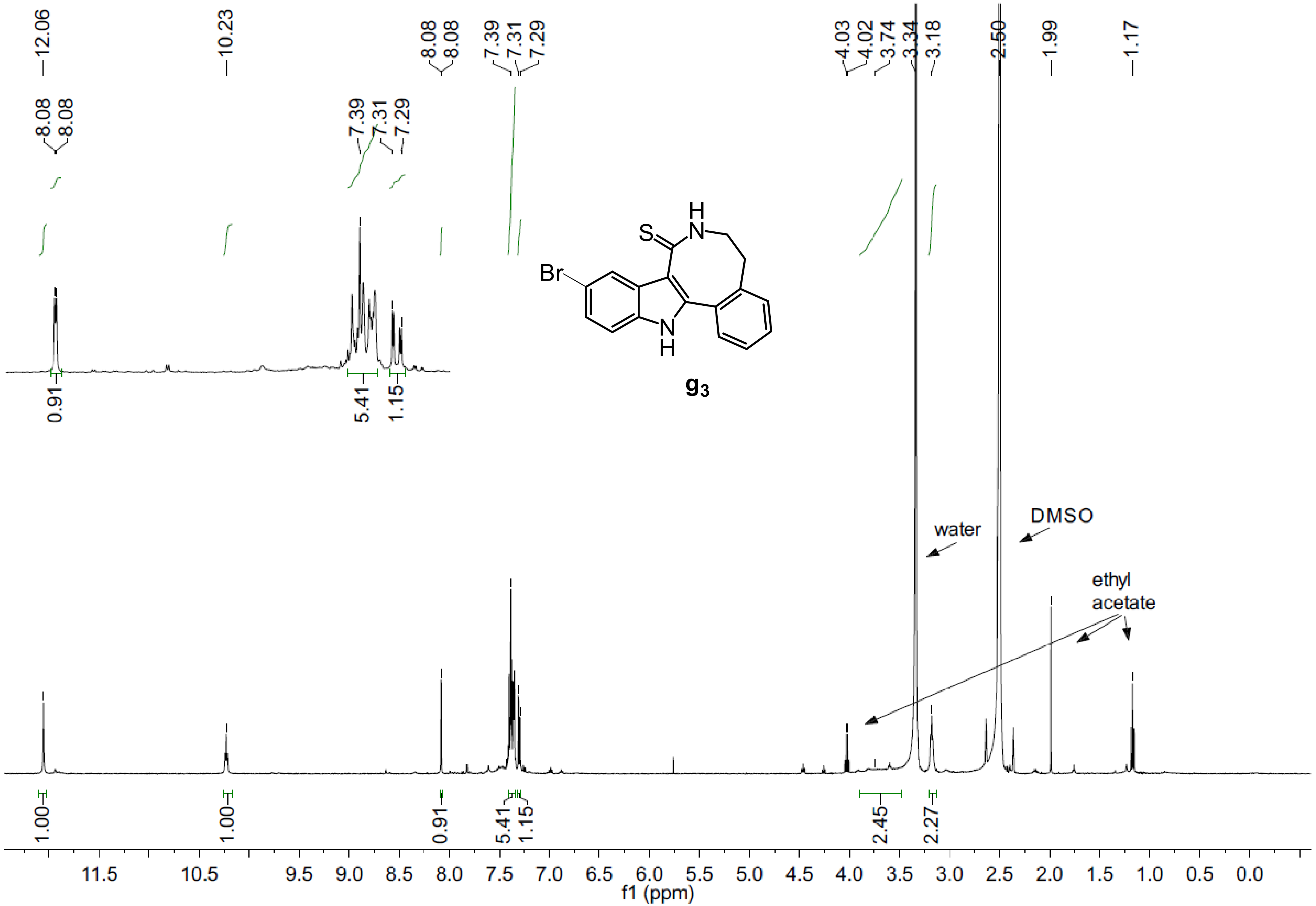

Figure S52. ^{1}H NMR spectrum of **g_3** in DMSO (500 MHz).

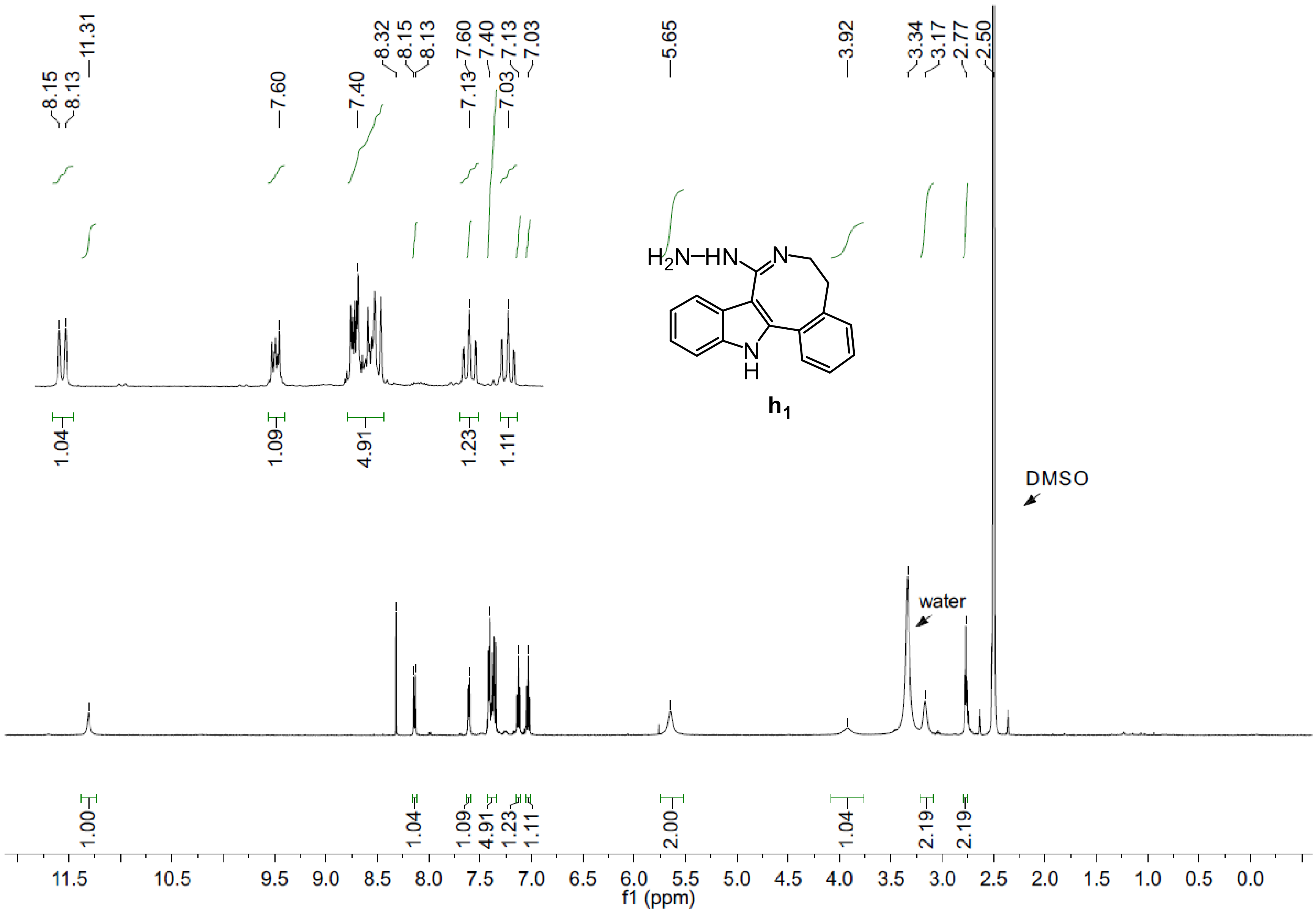

Figure S53. ^{1}H NMR spectrum of **h_1** in DMSO (500 MHz).

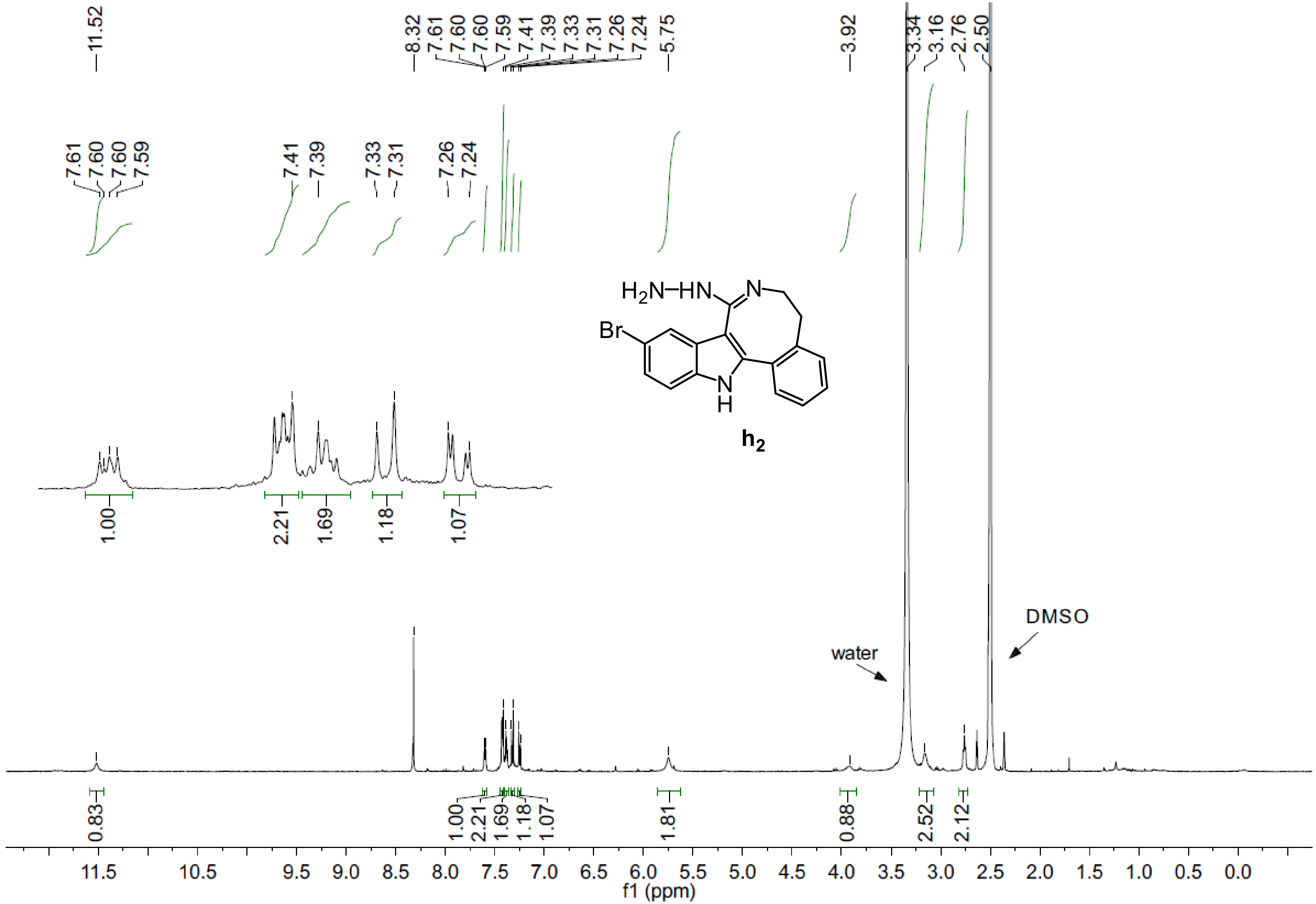

Figure S54. ^{1}H NMR spectrum of **h₂** in DMSO (500 MHz).

Generic Display Report

Analysis Info

Analysis Name	E:\Data\MS_MessService\74131000001.d	Acquisition Date	10/20/2020 3:08:25 PM
Method	tune_low_MS_Service_10_20.m	Operator	msc
Sample Name	IRKU357	Instrument	maXis
Comment	Kuznetcova/Anorg.Chem.<br>Ergebnis +/- 5ppm<br>ACN/MeOH + 1% H2O		

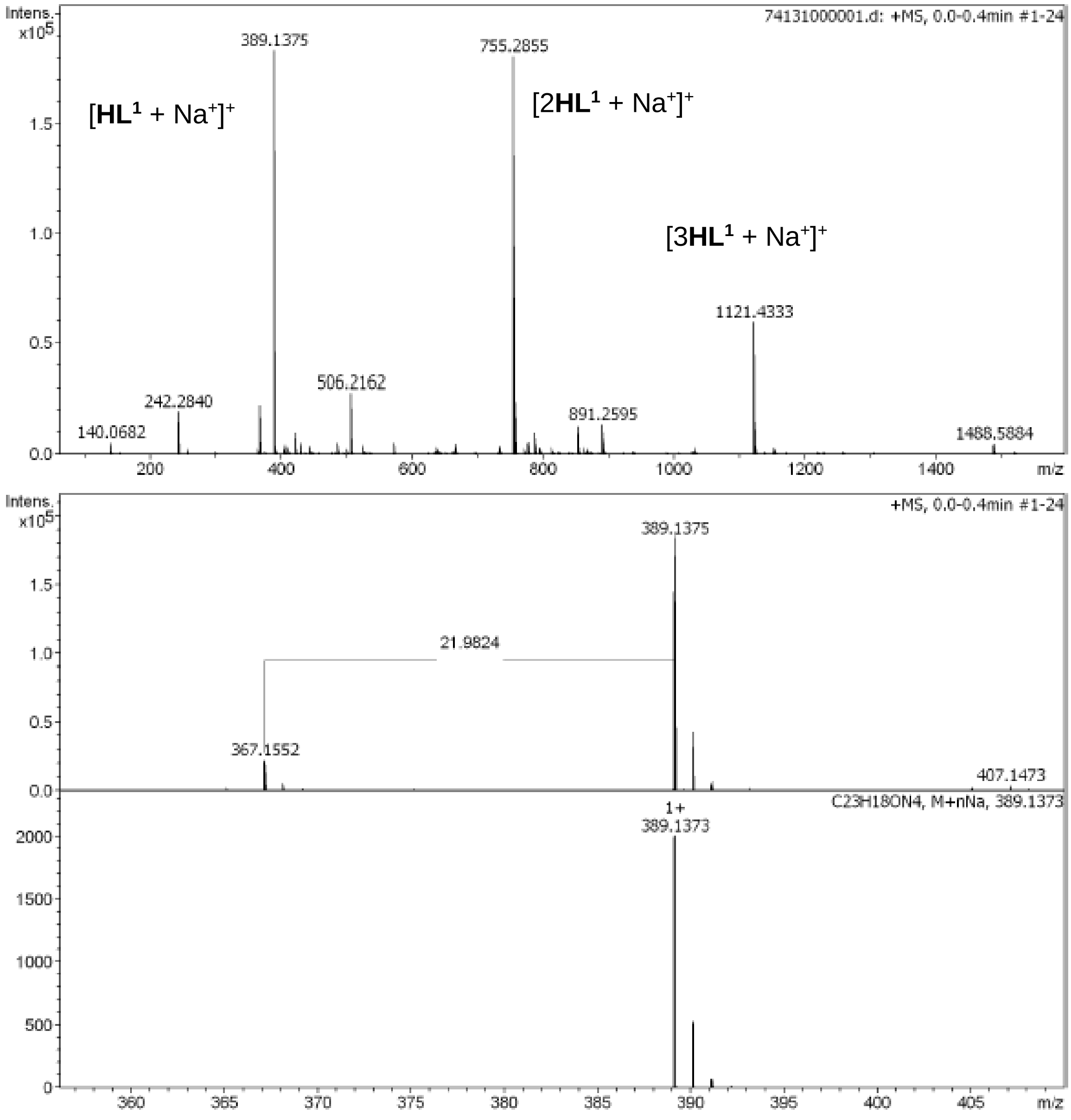

Bruker Compass DataAnalysis 4.1 printed: 10/20/2020 3:18:43 PM by: msc Page 1 of 1

Figure S55. High resolution ESI mass spectrum of HL1**.**

NMR numbering schemes

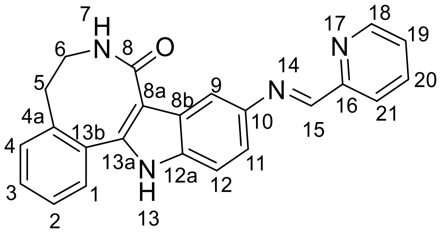

HL¹

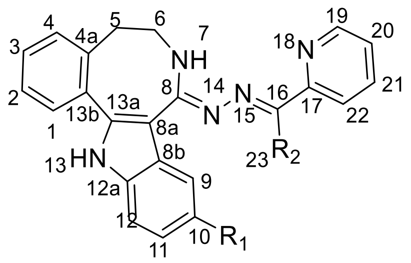

$R_1 = R_2 = H$ **HL²**

$R_1 = H, R_2 = Me$ **HL³**

$R_1 = Br, R_2 = H$ **HL⁴**

$R_1 = Br, R_2 = Me$ **HL⁵**

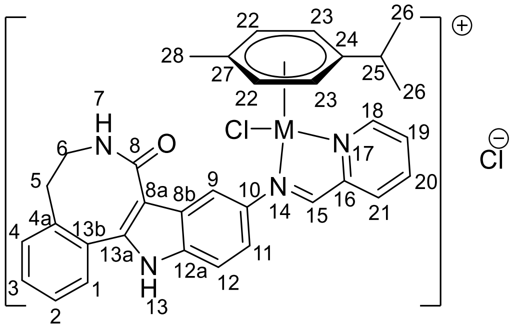

M = Ru^{II} **1a**

M = Os^{II} **1b**

Chart S1. Atom numbering schemes for assignment of resonances in ^{1}H and ^{13}C NMR spectra of **1a**, **1b** and **HL¹**–**HL⁵**.

Table S1. Crystal Data and Details of Data Collection for **f2**·CH_2Cl_2, **g2**·CH_2Cl_2 and **3**·3MeOH.

Compound	**f2**·CH_2Cl_2	**g2**·CH_2Cl_2	**3**·3MeOH
empirical formula	$C_{18}H_{16}Cl_2N_2O$	$C_{18}H_{16}Cl_2N_2S$	$C_{27}H_{33}Cl_2CuN_5O_3$
fw	347.23	363.29	610.02
space group	orthorhombic, *Pbca*	monoclinic, *I*2/*a*	monoclinic, $P2_1/c$
a, Å	17.5510(3)	21.566(4)	10.3799(6)
b, Å	9.6586(1)	7.4736(12)	35.2189(16)
c, Å	19.8163(4)	21.661(4)	7.5740(4)
α,°			
β, °		107.631(17)	98.3411(11)
γ, °			
V [Å^3]	3359.22(10)	3327.3(10)	2739.5(3)
Z	8	8	4
λ [Å]	0.71073	0.71073	0.71073
ρ_{calcd}, g cm^{-3}	1.373	1.450	1.479
cryst size, mm^3	0.28 × 0.23 × 0.19	0.20 × 0.18 × 0.14	0.20 × 0.15 × 0.05
T [K]	100(2)	100(2)	100(2)
μ, mm^{-1}	0.392	0.516	1.031
$R_1{}^a$	0.0452	0.0610	0.0370
$wR_2{}^b$	0.1379	0.1722	0.0943
GOF^c	1.080	0.971	1.052

^{a} $R_1 = \Sigma||F_o| - |F_c||/\Sigma|F_o|$. ^{b} $wR_2 = \{\Sigma[w(F_o^2 - F_c^2)^2]/\Sigma[w(F_o^2)^2]\}^{1/2}$. ^{c} GOF = $\{\Sigma[w(F_o^2 - F_c^2)^2]/(n - p)\}^{1/2}$, where *n* is the number of reflections and *p* is the total number of parameters refined.
